# Experimental Data-Mining Analyses Reveal New Roles of Low-Intensity Ultrasound in Differentiating Cell Death Regulatome in Cancer and Non-cancer Cells via Potential Modulation of Chromatin Long-Range Interactions

**DOI:** 10.3389/fonc.2019.00600

**Published:** 2019-07-12

**Authors:** Jiwei Wang, Bin Lai, Gayani Nanayakkara, Qian Yang, Yu Sun, Yifan Lu, Ying Shao, Daohai Yu, William Y. Yang, Ramon Cueto, Hangfei Fu, Huihong Zeng, Wen Shen, Susu Wu, Chunquan Zhang, Yanna Liu, Eric T. Choi, Hong Wang, Xiaofeng Yang

**Affiliations:** ^1^Department of Pharmacology, Centers for Metabolic Disease Research, Inflammation, Translational and Clinical Lung Research, Cardiovascular Research, Thrombosis Research, Philadelphia, PA, United States; ^2^Department of Microbiology, Centers for Metabolic Disease Research, Inflammation, Translational and Clinical Lung Research, Cardiovascular Research, Thrombosis Research, Philadelphia, PA, United States; ^3^Department of Immunology, Centers for Metabolic Disease Research, Inflammation, Translational and Clinical Lung Research, Cardiovascular Research, Thrombosis Research, Philadelphia, PA, United States; ^4^Department of Ultrasound, The Second Affiliated Hospital of Nanchang University, Nanchang, China; ^5^Department of Gastrointestinal Surgery, The Second Affiliated Hospital of Nanchang University, Nanchang, China; ^6^Department of Clinical Sciences, Lewis Katz School of Medicine at Temple University, Philadelphia, PA, United States; ^7^Division of Vascular and Endovascular Surgery, Department of Surgery, Lewis Katz School of Medicine at Temple University, Philadelphia, PA, United States

**Keywords:** ultrasound, cell death regulators, inflammatory pathways, cancer therapy, chromatin long-range interaction

## Abstract

**Background:** The mechanisms underlying low intensity ultrasound (LIUS) mediated suppression of inflammation and tumorigenesis remain poorly determined.

**Methods:** We used microarray datasets from NCBI GEO Dataset databases and conducted a comprehensive data mining analyses, where we studied the gene expression of 299 cell death regulators that regulate 13 different cell death types (cell death regulatome) in cells treated with LIUS.

**Results:** We made the following findings: (1) LIUS exerts a profound effect on the expression of cell death regulatome in cancer cells and non-cancer cells. Of note, LIUS has the tendency to downregulate the gene expression of cell death regulators in non-cancer cells. Most of the cell death regulator genes downregulated by LIUS in non-cancer cells are responsible for mediating inflammatory signaling pathways; (2) LIUS activates different cell death transcription factors in cancer and non-cancer cells. Transcription factors TP-53 and SRF- were induced by LIUS exposure in cancer cells and non-cancer cells, respectively; (3) As two well-accepted mechanisms of LIUS, mild hyperthermia and oscillatory shear stress induce changes in the expression of cell death regulators, therefore, may be responsible for inducing LIUS mediated changes in gene expression patterns of cell death regulators in cells; (4) LIUS exposure may change the redox status of the cells. LIUS may induce more of antioxidant effects in non-cancer cells compared to cancer cells; and (5) The genes modulated by LIUS in cancer cells have distinct chromatin long range interaction (CLRI) patterns to that of non-cancer cells.

**Conclusions:** Our analysis suggests novel molecular mechanisms that may be utilized by LIUS to induce tumor suppression and inflammation inhibition. Our findings may lead to development of new treatment protocols for cancers and chronic inflammation.

## Introduction

Ultrasound, alone or combined with contrast agent microbubbles, have numerous applications ranging from being a well-established diagnostic tool (1, 2) to a method of drug delivery (3). The application of microbubbles and ultrasound to deliver nanoparticle carriers for drug and gene delivery is a research area that has expanded greatly in recent years. Recent studies reported that utilization of ultrasound contrast microbubbles causes the so-called “sonoporation” effect (4, 5), which has been recognized as a significant factor in transient disruption of cell membrane permeability (6) that allows easier transport of extracellular compounds into the cytoplasm of viable cells (7). Ultrasound therapy now is widely used in clinical practice, and clinical/translational research in the treatment of various human malignancies and pathologies including breast cancer, leukemia, lymphoma, melanoma, pancreatic neuroendocrine tumors (8), hepatic cancer, nasopharyngeal cancers, glioma, ovarian cancer, colon cancer, gastric cancer (9), sarcoma (10–12), stroke (13), prostatic hyperplasia, renal masses (14), treatment of abdominal subcutaneous adipose tissue (15), bone repair (16), osteoarthritis (17), and carpal tunnel syndrome (18). So far, several therapeutic ultrasound formats have been developed including high intensity focused ultrasound (10) and low-intensity pulsed ultrasound (19). Recently, several clinical trials and experimental data verified the ability of ultrasound to elicit anti-inflammatory and tissue repair/regeneration responses (20, 21), suggesting the potential of using ultrasound as a novel therapeutic method (6, 22–25).

Cell death pathways have gained attention as novel therapeutic targets for treatment of cancers (26, 27) and inflammation (28, 29). We and others have reported that activated T cells survive by upregulating anti-cell death proteins such as Bcl-xγ (30), translationally controlled tumor protein (TCTP) (31) and inhibiting severe acute respiratory syndrome (SARS) virus E protein (32). Moreover, CD4+CD25+Foxp3+ regulatory T cells readily undergo apoptosis (33) by upregulating pro-apoptotic protein Bax (34) and downregulating anti-apoptotic regulator TCTP (35, 36). In addition, metabolic disease-risk factors such as hyperlipidemia (37–39), hyperhomocysteinemia (40) and chronic kidney disease (41) accelerate vascular diseases by inducing inflammatory cell death (pyroptosis) or pyrop-apoptosis (37, 40). Interestingly, LIUS was reported to induce apoptosis, autophagy or necrosis in cancer cells including nasopharyngeal cancer cells (42), laryngeal cancer cells (43), hepatocellular cancer cells (43, 44), leukemia cells (45), lymphoma cells (46), and osteosarcoma cells (47). In contrast, LIUS affects various non-cancer immune cells and other cells by regulation of inflammation. For example, ultrasound promotes vasodilation, enhances blood flow, promotes fibroblast and osteoblast proliferation, and increases other cellular components leading to wound healing (21). Moreover, LIUS was reported to suppress synovial cell proliferation (48), affect mesenchymal stem cell migration (49), enhance the regeneration of myofibers (50), reduce the expression of inflammatory mediators (25), promote skin fibroblast proliferation (51), and chondrocyte and osteoblast proliferation (52). However, it remains unclear how LIUS can distinguish cancer cells from non-cancer cells and induce differential biological responses. Further, molecular and cellular mechanisms underlying LIUS mediated inflammation inhibition and cancer suppression effects are not understood.

In order to broaden our understanding of LIUS-mediated effects in cellular context, we hypothesized that LIUS may induce differential gene expression patterns of cell death regulators in cancer cells and non-cancer cells. Therefore, in this study, we analyzed the expression pattern of 299 cell death regulators in LIUS-treated cancer cells and non-cancer cells. These genes were responsible for regulating 13 different types of cell death mechanisms including apoptosis, MPT-driven necrosis, necroptosis, ferroptosis, pyroptosis, parthanatos, entotic cell death, NETotic cell death, lysosomal dependent cell death (LCDC), autophagy dependent cell death (ADCD), immunogenic cell death (ICD), mitotoic death, and anoikis. Herein, we will refer to the 299 cell death regulators of the 13 different types of cell death pathways that we included in this study as the cell death regulatome. Our data indicated that LIUS exerts a differential gene expression pattern of the cell death regulatome in cancer cells and non-cancer cells. Furthermore, our data implies that thermal effects and osmotic shear stress (OSS) associated with LIUS may potentially play a role in inducing the differential gene expression patterns of the cell death regulatome that we observed. Also, we observed that LIUS has the tendency to induce antioxidant effects in non-cancer cells, which may also contribute to the differential gene expression patterns of the cell death regulatome in LIUS-treated cancer cells and non-cancer cells. Most interestingly, we observed that the cell death regulator genes modulated by LIUS in cancer cells and non-cancer cells have unique chromatin long range interaction (CLRI) sites. Chromatin looping enables CLRIs, that gives the opportunity to gene promoters to interact with distal regulatory elements (53). Rapid development of technologies such as chromosome conformation capture-sequencing (3C-seq) (54), circularized chromosome conformation capture-sequencing (4C-seq) (55, 56) and chromosome conformation capture carbon copy-sequencing (5C-seq) (57) that capture chromosome conformation allow determination of interactions between the target genes and CLRI sites. The CLRI may enhance and modulate the expression of genes of interest. Differences in chromatin long-range interaction patterns between genes have previously been hypothesized to influence alternative splicing (58) and the transcription of inflammatory genes such as cytokine (59), cytokine receptor (60) and cardiovascular disease-causative genes (61). Therefore, we suggest that the unique CLRI seen in genes modulated by LIUS in cancer cells and non-cancer cells may play a role in producing a differential response.

## Materials and Methods

### Expression Profile of Cell Death Genes in Ultrasound-Treated, Mild Hyperthermia-Treated, and Oscillatory Shear Stress-Treated Cells

Microarray datasets were collected from National Institutes of Health (NIH)-National Center for Biotechnology Information (NCBI) -GEO Dataset (https://www.ncbi.nlm.nih.gov/gds/) databases and analyzed with GEO2R (https://www.ncbi.nlm.nih.gov/geo/geo2r/). The numbers of GEO datasets that are as follows: GSE10212, GSE45487, GSE70662, GSE10043, GSE39178, GSE60152, GSE28546, GSE90, GSE3181. The detailed information of these GEO datasets was shown in Table 1.

**Table 1:** Nine microarray datasets were analyzed in this study.

**Treatment**	**Disease**	**GEO ID**	**Organism**	**Cell line**	**Method/Parameter**	**Time**	**PMID**
LIUS	Cancer	GSE10212	Homo sapiens	Lymphoma U937 cells	0.3 W/cm^2^, 1.0 MHz.	1 min	18571840
	Non-cancer	GSE45487	Mus musculus	MC3T3-E1 preosteoblast cells	0.03 W/cm^2^, 1.5 MHz.	20 min	24252911
		GSE70662	Rattus norvegicus	Bone marrow cells from femora	N/A	15 min/day × 7 day	N/A
Mild hyperthermia	Cancer	GSE10043	Homo sapiens	Lymphoma U937 cells	41°C	30 min	18608577
	Non-cancer	GSE39178	Homo sapiens	Fibroblast OUMS-36 cells	41°C	30 min	23311377
Oscillatory shear stress	Cancer	N/A	N/A	N/A	N/A	N/A	N/A
	Non-cancer	GSE60152	Homo sapiens	Human lymphatic endothelial cells	1 dyn/cm^2^,1/4 Hz	24 h	26389677
Others	Cancer	GSE90	Homo sapiens	Colorectal carcinoma-derived cell lines	p53 knock-out	N/A	12438652
	Non-cancer	GSE28546	Homo sapiens	Mesenchymal Stem Cells	p53 knock-down	N/A	N/A
		GSE3181	Mus musculus	Cardiomyocyte	SRF knock-out	N/A	16368687

*LIUS, Low-intensity ultrasound; PMID, PubMed ID number; p53, tumor protein p53; SRF, serum response factor*.

Two hundred and ninety nine cell death regulators that participate in 13 different types of cell death were analyzed in our study, and 91 cell death regulators regulate multiple cell death types. Of note, several cell death forms are reported recently, including MPT-driven necrosis, parthanatos, entotic cell death, NETotic cell death, lysosome dependent cell death, immunogenic cell death, mitotic death, and anoikis. All detailed information of these cell death regulators and 13 types of cell death was shown in Table 2.

**Table 2:** The gene list of cell death regulators.

**Cell death type**	**Related gene**	**Gene number**	**PMID**	**Gene detailed information**
Apoptosis	FASLG,FAS,TNF,TNFRSF1A,TNFSF12,TNFRSF25,TNFSF10,TNFRSF10A, TNFRSF10B,FADD,TRADD,RIPK1,AATF,CASP8,CFLAR,DIABLO,HTRA2, XIAP,BIRC2,BIRC3,BIRC5,BIRC7,BIRC6,APAF1,CASP9,AIFM1,CAD, BCL2,BCL2L1,MCL1,BCL2L2,BAG1,BAG2,BAG3,BAG4,BAG5,BAG6,BAK1, BAX,BLK,BCL2L11,BID,BIK,BBC3,PMAIP1,BCL10,BAD,BOK,YWHAZ, YWHAE,YWHAB,YWHAQ,YWHAG,YWHAH,AVEN,MYC,CASP3,CASP6, CASP7,CASP10,PARP1,NUMA1,DFFA,TP53,CDKN1A,CDKN1B,CDK1, E2F1,E2F2,E2F3,E2F4,E2F5,E2F6,E2F7,E2F8,RB1,CCND1,MAPK8,MAPK14, MAPK1,PIK3CA,PIK3CB,PIK3CG,PIK3CD,PIK3R1,PIK3R2,PIK3R3, PIK3R4, PIK3R5,PIK3R6,PIK3C2A,PIK3C2B,PIK3C2G,PIK3C3, AKT1, AKT2, AKT3, NFKB1, NFKB2, RELA, RELB, REL	102	17562483, 14763159	see Table S1
MPT-driven necrosis	SLC25A4, PPID, ATP5G1, ATP5G2, ATP5G3, SLC25A3, SPG7, VDAC1, VDAC2, VDAC3, BAD, BAK1, BAX, BCL2, BCL2L1, BID, CKMT1A, CKMT1B, GSK3B, HK1, HK2, TP53, TSPO, PRKCE, HMGB1	25	27161573	see Table S2
Necroptosis	FASG, FAS, TNF, TNFRSF1A, TNFSF12, TNFRSF25, TNFSF10, TNFRSF10A, TNFRSF10B, TLR3, TLR4, IFNAR1, IFNAR2, TRADD, RIPK1, RIPK3, MLKL, PGAM5, CYLD, BIRC2, BIRC3, CASP8, FADD, DNM1L, BCL2L1	25	27429198, 26968619	see Table S3
Ferroptosis	ROS1, TFRC, ACSF2, EMC2, RPL8, IREB2, SLC7A11, CS, ATP5G3, GPX4, GCLC, ACSL4, LPCAT3, CARS, SLC1A5, GLS2, GOT1, HSPB1, TP53, FDFT1, HSPA5, NFE2L2, MT1G, DPP4, FANCD2, CISD1, ROS1	26	27048822, 29362479	see Table S4
Pyroptosis	AIM2, MEFV, CASP1, CASP4, GSDMD, PYCARD, NLRC4, NLRP1, NLRP3, Nlrp1b, Naip5, IL18, IL1B, CASP3, CASP5, PANX1, P2RX7, PRKN, GSDME, IFNGR1, IFNAR1, TLR4, TLR7, CGAS, TMEM173, DDX58	26	27404251, 29362479	see Table S5
Parthanatos	PARP1, AIFM1, ADPRHL2, RNF146, MIF, HK1	6	29362479	see Table S6
Entotic cell death	CHD1, CTNNA1, RHOA, ROCK1, ROCK2, DIAPH1, MKL1, MKL2, SRF, EZR, KRAS, RAC1, MAP1LC3B, ATG5, ATG7, PIK3C3, PIKFYVE, ITGB1, ITGB3, ITGA5, ITGAV, ITGA1, ITGA6	23	29362479	see Table S7
NETotic cell death	RAF1, MAP2K1, MAP2K2, MAP2K3, MAP2K4, MAP2K5, MAP2K6, MAP2K7, MAPK1, ROS1, DECR1, ELANE, MPO, PADI4	14	29362479	see Table S8
LDCD	ROS1, DRAM1, STAT3, CTSB, CTSL, Serpina3g, BID, BAX, BCL2, XIAP, PRTN3, HSPA1A	12	29362479	see Table S9
ADCD	ULK1, ULK2, ATG3, ATG4A, ATG4B, ATG4C, ATG4D, ATG5, BECN1, ATG7, GABARAP, GABARAPL1, GABARAPL2, MAP1LC3A, MAP1LC3B, MAP1LC3B2, MAP1LC3C, ATG10, ATG12, ATG16L1, ATG16L2, PIK3C3, MAPK8, PIK3CA, PIK3CB, PIK3CG, RIPK1, MTOR, MTMR14, BCL2, NAF-1, CFLAR, RUBCN, TP53	34	15928714, 20865012	see Table S10
ICD	CALR, EIF2S1, EIF2AK3, BCAP31, BAK1, BAX, VAMP1, SNAP25, PDIA3, CD47, P2RY2, P2RX7, LAMP1, ROCK1, PANX1, ENTPD1, NT5E, TLR3, CGAS, IFNAR1, CXCL10, TREX1, HMGB1, ANXA1, TLR2, TLR4, AGER, FPR1, CASP3, CASP8	30	29362479	see Table S11
Mitotic death	TP53, BCL2, ATM, ATR, CHEK1, CHEK2, CDC25A, MAPKAPK2, WEE1, MYT1, CDC25B, CDC25C, CCNE1, CCNB1, CDC20, MAD1L1, MAD2L1, BUB1, BUB3, BUB1B, CENPE, PLK1, PLK2, PLK3, PLK4, PLK5, AURKA, AURKB, AURKC, TTK	30	26491220	see Table S12
Anoikis	ITGB1, ITGB3, ITGA5, ITGAV, ITGA1, ITGA6, PTK2, SRC, ILK, MAPK8, MAPK14, MAPK1, PIK3CA, PIK3CB, PIK3CG, PIK3CD, PIK3R1, PIK3R2, PIK3R3, PIK3R4, PIK3R5, PIK3R6, PIK3C2A, PIK3C2B, PIK3C2G, PIK3C3, AKT1, AKT2, AKT3, CAV1, EGFR, INSR, PDGFRA, PDGFRB, HGF, KDR, BCL2L11	37	23830918	see Table S13
	Total gene number	390–91 = 299		

*The latest definitions, morphological features, main molecular features, and detection methods of 13 different cell death types (see Tables S14, S15)*.

### Statistical Analysis of Microarray Data

As we reported (62, 63), we applied a statistical method similar to that meta-analysis and analyzed the expression of four house-keeping genes (CHMP2A, PSMB4, ACTB, and GAPDH) in all GEO datasets regardless of species that were chosen for this study. The house-keeping gene list was extracted from related report (64). Briefly, the variations between the expression of housekeeping genes between treatment and control groups vary from −1.27 to 1.28. As this variation was very narrow, we concluded that the datasets (Table 3) are of high quality. The target genes with expression change more than 1.5-fold were defined as the upregulated genes, while genes with their expression decreases more than 1.5-fold were defined as downregulated genes.

**Table 3:** The expression level of housekeeping genes in all the microarray datasets that were used for this study were not significantly changed.

**Housekeeping gene**	**UniGene ID**		**Fold Change**								
	**Hs**	**Mm**	**GSE10212**	**GSE45487**	**GSE70662**	**GSE10043**	**GSE39178**	**GSE60152**	**GSE90**	**GSE28546**	**GSE3181**
CHMP2A	12107	295670	1.076	−1.025	−1.151	1.183	1.160	−1.002	/	−1.141	−1.005
PSMB4	89545	368	−1.015	−1.019	−1.044	1.032	1.087	−1.087	−1.271	1.072	−1.071
ACTB	520640	391967	1.013	−1.002	−1.094	1.063	1.128	−1.015	1.037	−1.207	1.037
GAPDH	544577	304088	1.009	−1.005	1.240	1.139	1.284	−1.031	1.041	−1.215	−1.011

*These housekeeping genes were extracted from related report (PMID: 23810203). CHMP2A, charged multivesicular body protein 2A; PSMB4, proteasome subunit beta 4; ACTB, actin beta; GAPDH, glyceraldehyde-3-phosphate dehydrogenase*.

### Ingenuity Pathway Analysis

We utilized Ingenuity Pathway Analysis (IPA, Ingenuity Systems, http://pages.ingenuity.com/rs/ingenuity/images/IPA_data_sheet.pdf) to characterize clinical relevance, and molecular and cellular functions related to the identified genes in our microarray analysis. The differentially expressed genes were identified and uploaded into IPA for analysis. The core and pathways analysis was used to identify molecular and cellular pathways as we have previously reported (63, 65).

### Chromatin Long-Range Interaction Analysis

The chromatin long-range interaction data were collected from the Hi-C data deposited in the 4D Genome database (https://4dgenome.research.chop.edu) as a tabulated text file (66). As we reported (62, 67), we extracted the data related to interact gene and interaction sites interacting with LIUS regulated-cell death genes, then calculated the distances between the interaction sites and LIUS-modulated gene promoters. The resulting filtered data was imported into Microsoft Excel and raw interaction distances calculated as the differences between gene start coordinates. An AWK script was used to determine whether the LIUS-modulated gene promoters were downstream or upstream of its partner in each interaction pair; and to add this information to the data file. The signs of distance values were then updated, with downstream entries designated as positive; and upstream values designated as negative. Distance distributions for all upregulated and all downregulated LIUS-modulated genes were compared by groups overall, respectively.

## Results

### LIUS Change the Gene Expression of the Cell Death Regulatome in Cancer Cells and Induce Cell Death Regulators That Regulate Inflammation in Non-cancer Cells

As listed in Table 4, many publications have shown that LIUS induces cell death pathways in cancer cells. In contrast, LIUS exerts other therapeutic effects in non-cancer cells such as modulation of cell proliferation, regulation of cell migration, enhancement of regeneration etc. However, how cancer cells and non-cancer cells produce a differential response to LIUS remain unknown (68). A previous study reported a comparison of the effects of LIUS treatment in cancer cells including breast cancer melanoma, lung cancer with non-cancer cells such as foreskin fibroblasts, and amniotic fluid epithelial cells using cell death assay. However, as we pointed out in Table 5, this comparison gives limited information; and the mechanisms that induce differential signaling response in cancer cells and non-cancer cells were not examined in detail (68).

**Table 4:** An extensive literature survey confirmed that LIUS exerts cancer suppression effects via inducing cell death pathways in cancer cells, but manifests cyto-protective effects by modulating cell proliferation and anti-inflammatory effects in non-cancer cells.

**Main function**		**Ultrasound**			**Cell/Tissue**	**Mechanism**	**Possible involved pathway**	**PMID**
		**Intensity (W/cm^**2**^)**	**Frequency (MHz)**	**Exposure time**				
Cancer	Cancer suppression	1.35	1.7	24 h	Nasopharyngeal carcinoma cells	Induce cellular apoptosis and autophagy	Unclear	22977587
		1.75-2.5	1	0.5 min	Laryngeal carcinoma cells	Induce cellular apoptosis	Cav-1/STAT3 signaling pathway	27289429
		3	1.2	1 min	Hepatocellular carcinoma cells	Induce cellular apoptosis and necrosis	Mitochondrial pathway and oxidative stress pathway	20498470
		0.3	1	1 min	Leukemia cells	Induce cellular apoptosis	Unclear	15808400
		0.045 - 0.09	0.4–0.62	1.5–3 min	Lymphoma cells	Induce cellular apoptosis and lysis	Unclear	27635161
		2.0-3.0	1	7 min	Osteosarcoma cells	Induce cellular apoptosis	ROS-related mitochondrial pathway	26161801
Non-cancer	1) Bone fracture healing; 2) soft-tissue regeneration; 3) Inhibiting inflammation	0.03	3	5–15 min	Synovial cells	Suppresses synovial cell proliferation	Integrin/FAK/MAPK pathway	25096496
		0.03	1.5	20 min/day for 3 days	Mesenchymal Stem Cell	Affects mesenchymal stem cells migration	SDF-1/CXCR4 signaling	25181476
		0.03	1.5	20 min/day for 2–8 days	Gastrocnemius muscle laceration injury model/Myoblastic cell	Enhances the regeneration of myofibers	Unclear	20381949
		0.03-0.20	1	10 min/day for 5 days	Arthritis model (Freund's adjuvant injection)	Reduced the expression of inflammatory mediators	Unclear	22289897
		0.03	1.5	11 min	Human Skin Fibroblasts	Promotes cell proliferation	Rho/ROCK/Src/ERK signaling pathway	15485877
		0.03	1.5	20 min/d for 2–16 weeks	Partial patellectomy	Promotes chondrocytes and osteoblasts proliferation	Regulation of VEGF expression	18378382

**Table 5:** Significant novel findings of our study (highlighted in red).

	**Lejbkowicz and Salzberg (68)**		**Our study**	
Low intensity ultrasound	√	0.33 W/cm^2^, 2 MHz, 4 min	√	0.3 W/cm^2^,1.0 MHz,1 min; 0.03 W/cm^2^,1.5 MHz, 20 min; LIPUS,15 min/day × 7day
Cancer vs. non-cancer	√	Cancer cells (Breast carcinoma, Melanoma, Lung carcinoma) vs. Non-cancer cells Foreskin fibroblast, Amniotic fluid epithelial)	√	Cancer cells(Lymphoma) vs. Non-cancer cells (Preosteoblast cells, Bone marrow cells)
Cell death test	√	Trypan blue exclusion test and cell multiply ability	√	Analyze 299 cell death related gene expression from Microarray assay
Cell death type analysis	×	N/A	√	Analyze 13 types of cell death
				Includes Apoptosis, Mitochondrial permeability transition-driven necrosis, Necroptosis, Ferroptosis, Pyroptosis, Parthanatos, Entotic cell death, NETotic cell death, Lysosome dependent cell death, Autophagy dependent cell death, Immunogenic cell death, Mitotic death, and Anoikis
Mechanisms	×	N/A	√	IPA analyze cell death signal pathways
				Thermal and non-thermal effects
				Oxidative stress
				Chromosome long-range interaction

*A previous study reported the sensitivity differences of LIUS-induced cell death between cancer cells and non-cancer cells. However, this report did not describe any molecular mechanisms that may contribute to the differences reported*.

We hypothesized that LIUS induces differential responses in cell death pathways in cancer cells vs. non-cancer cells by modulating the gene expression of cell death regulators. To examine this hypothesis comprehensively, we collected 299 genes that regulate all the 13 cell death types including apoptosis, mitochondrial permeability transition (MPT)-driven necrosis, necroptosis, ferroptosis, pyroptosis, parthanatos, entotic cell death, NETotic cell death, lysosome dependent cell death (LDCD), autophagy dependent cell death (ADCD), immunogenic cell death (ICD), mitotic death, and anoikis (69) (Table 2). As mentioned above, all the cell death regulators that we included in this study are collectively referred to as cell death regulatome. Of note, among 299 genes, 91 genes regulate multiple types of cell death. In addition, we found three microarray datasets deposited in the NIH-NCBI GeoDatasets database, which were conducted on human lymphoma cells treated with LIUS and two non-cancer cells such as mouse MC3T3-E1 pro-osteoblast cell and rat bone marrow cell, treated with LIUS (Table 1).

**Figure 1:**
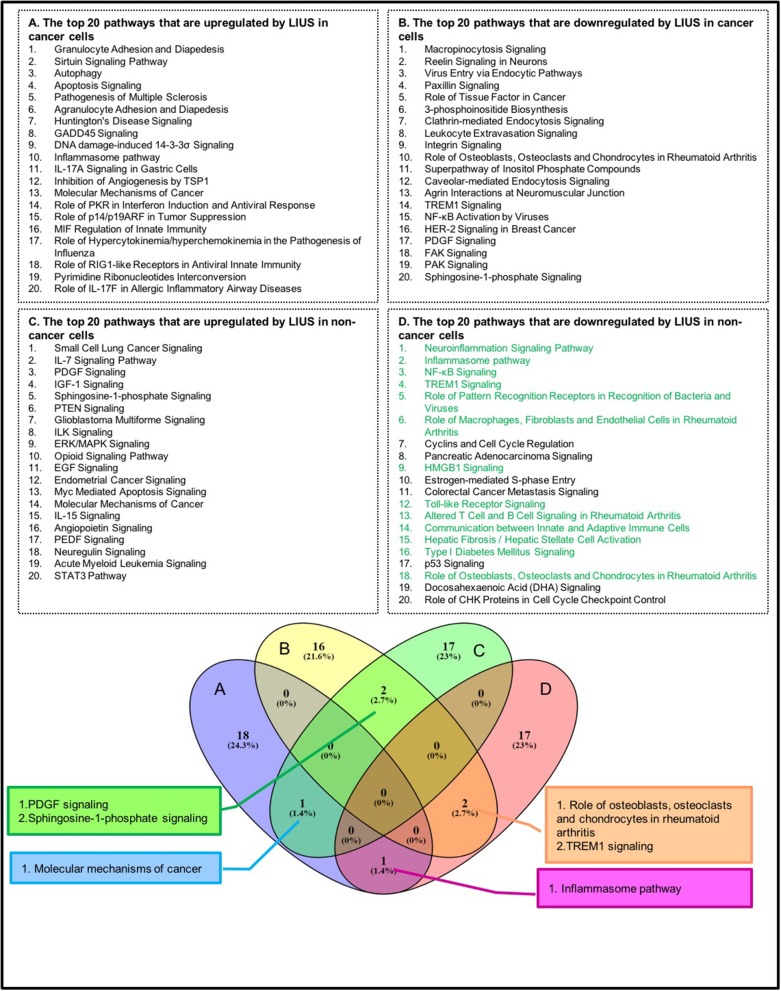
The top 20 pathways that are modulated by LIUS in cancer cells and non-cancer cells are not shared. Most of the cell death regulator genes downregulated by LIUS in non-cancer cells are responsible for mediating inflammatory signaling pathways. The green highlight pathways are pro-inflammatory pathways.

As shown in Table 6, among the 299 genes analyzed, LIUS induced upregulation of 13 genes in lymphoma cells including BOK, CASP10, CYLD, DPP4, EZR, ATG3, ATG16L1, VAMP1, CXCL10, ANXA1, FPR1, PANX1, and TP53; and downregulated 12 genes such as HK2, CASP5, TLR7, MAP2K5, CD47, ATM, CDC25C, TTK, SRC, PDGFRA, ITGB1, and ITGB3. When conducted ingenuity pathway analysis (IPA) on upregulated and downregulate genes by LIUS treatment in cancer cells, it revealed that none of the signaling pathways modulated by the two groups were shared (Figure 1). Therefore, this may suggest that the cell death regulators modulated by LIUS treatment activate intricate signaling mechanisms that may cause an impact on cell death in cancer cells.

**Table 6:** LIUS exerts a profound effect on the expression of cell death regulators in cancer cells and non-cancer cells.

	**LIUS in cancer cells**			**LIUS in non-cancer cells**		
	**Gene**	**Fold change**	**Cell death type**	**Gene**	**Fold change**	**Cell death type**
Upregulate	BOK	1.609	Apoptosis	MYC	1.519	Apoptosis
	CASP10	1.747	Apoptosis	SRF	1.528	Entotic cell death
	CYLD	1.599	Necroptosis	NT5E	1.657	ICD
	DPP4	7.195	Ferroptosis	BAG6	1.592	Apoptosis
	EZR	1.672	Entotic cell death	NUMA1	1.866	Apoptosis
	ATG3	3.273	ADCD	IREB2	1.857	Ferroptosis
	ATG16L1	1.706	ADCD	PTK2	1.549	Anoikis
	VAMP1	1.561	ICD	PDGFRA	1.812	Anoikis
	CXCL10	1.564	ICD	AKT3	2.670	Apoptosis, Anoikis
	ANXA1	4.294	ICD			
	FPR1	4.701	ICD			
	PANX1	1.913	Pyroptosis, ICD			
	TP53	2.723	Apoptosis, MPT-driven necrosis, Ferroptosis, ADCD, Mitotic death			
	Gene number	13	Gene number	9		
Downregulate	HK2	−1.860	MPT-driven necrosis	BIRC5	−2.642	Apoptosis
	CASP5	−3.734	Pyroptosis	BIK	−2.882	Apoptosis
	TLR7	−5.535	Pyroptosis	CDK1	−1.806	Apoptosis
	MAP2K5	−3.208	NETotic cell death	E2F1	−2.946	Apoptosis
	CD47	−15.995	ICD	E2F8	−2.603	Apoptosis
	ATM	−2.035	Mitotic death	CCND1	−1.945	Apoptosis
	CDC25C	−1.933	Mitotic death	GCLC	−1.513	Ferroptosis
	TTK	−1.713	Mitotic death	CASP1	−1.537	Pyroptosis
	SRC	−1.985	Anoikis	CASP4	−2.122	Pyroptosis
	PDGFRA	−3.633	Anoikis	PYCARD	−2.779	Pyroptosis
	ITGB1	−7.891	Anoikis, Entotic cell death	NLRP3	−2.402	Pyroptosis
	ITGB3	−8.536	Anoikis, Entotic cell death	IL18	−2.292	Pyroptosis
				IL1B	−2.162	Pyroptosis
				IFNGR1	−1.595	Pyroptosis
				TLR7	−2.854	Pyroptosis
				MAP2K7	−1.622	NETotic cell death
				ATG10	−2.234	ADCD
				CD47	−1.797	ICD
				ENTPD1	−3.351	ICD
				TLR2	−4.067	ICD
				ATM	−3.890	Mitotic death
				MYT1	−2.969	Mitotic death
				BUB1	−2.000	Mitotic death
				BUB1B	−2.395	Mitotic death
				TTK	−1.832	Mitotic death
				HGF	−2.136	Anoikis
				KDR	−1.947	Anoikis
				ITGA6	−2.168	Anoikis, Entotic cell death
				TLR4	−3.319	Necroptosis, Pyroptosis, ICD
				TRADD	−1.607	Apoptosis,Necroptosis
				BCL2	−10.126	Apoptosis, MPT-driven necrosis, LDCD, ADCD, Mitotic death
	Gene number		12	Gene number		31

*Of note, LIUS has the tendency to downregulate cell death regulators in non-cancer cells*.

LIUS induced the expression of 9 cell death regulator genes in non-cancer cells including MYC, SRF, NT5E, BAG6, NUMA1, IREB2, PTK2, PDGFRA, and AKT3; and downregulated 31 genes among 299 genes examined (Table 6). Similar to the observation seen in cancer cells, the IPA analysis demonstrated that LIUS induced upregulated and downregulated cell death regulators in non-cancer cells activate distinct cell signaling pathways. Of note, the 31 cell death regulators that were downregulated by LIUS treatment seem to attenuate several pro-inflammatory pathways including neuroinflammation signaling pathway, inflammasome pathway, NF-κB signaling, TREM1 signaling, role of pattern recognition receptors in recognition of bacteria and viruses, role of macrophages, fibroblasts and endothelial cells in rheumatoid arthritis, HMGB1 signaling, Toll-like receptor signaling, altered T cell and B cell signaling in rheumatoid arthritis, communication between innate and adaptive immune cells, hepatic fibrosis/hepatic stellate cell activation, type I diabetes mellitus signaling, and role of osteoblasts, osteoclasts and chondrocytes in rheumatoid arthritis (Figure 1). Therefore, this data suggests that LIUS treatment may significantly impact the inflammatory status of non-cancer cells.

Taken together, these results suggest that *first*, LIUS exerts a profound effect on cell the gene expression of the cell death regulatome in cancer cells and non-cancer cells; *second*, LIUS treatment has the tendency to reduce the expression of cell death regulators than activating them in non-cancer cells; *third*, in non-cancer cells, the death regulators that are downregulated by LIUS attenuate several inflammatory signaling pathways. These findings are well-correlated with our previous publication where we reported that LIUS mediated upregulation of anti-inflammatory regulators (2). Furthermore, this data suggest that the majority of LIUS-downregulated cell death regulators in non-cancer cells are functional as inflammatory regulators, which confirmed our updated understanding that inflammation and cell death pathways are often inter-connected (70). For example, the binary classification of mammalian cell death regulators, such as caspases, as either apoptotic or inflammatory (71) is now obsolete. Emerging data indicate that all mammalian caspases are intricately involved in the regulation of inflammation and immunity (72).

### LIUS Inhibits the Expression of a List of Inflammation-Related Cell Death Regulators, Potentially via Transcription Factors TP53-, and SRF-Mediated Pathways in Cancer Cells and Non-cancer Cells Respectively

As mentioned above, we observed that cell death regulatome is differentially regulated in cancer cells and non-cancer cells when exposed to LIUS treatment. In order to explain this observation, we hypothesized that LIUS may modulate the expression of cell death-related transcription factors in both cancer and non-cancer cell groups. We found that tumor suppressor gene TP53 (73) was upregulated by LIUS treatment in cancer cells; and that serum response factor (SRF) was upregulated by LIUS in non-cancer cells (Table 6). To determine whether LIUS-induced TP53 plays roles in regulating the cell death regulator genes modulated by LIUS, we examined the expression of cell death regulators in TP53 gene deficiency cell microarray datasets. As shown in Table 7, we found that a list of LIUS-downregulated cell death regulators such as CD47, protein kinase ataxia-telangiectasia mutated (ATM) (74), caspase-1 (CASP1) (71), CASP4, CD47, and BCL2 were upregulated in human TP53 knock-down (KD) mesenchymal stem cell datasets. These results suggest that LIUS has the potential to regulate cell death regulator expression in cancer cells partially via inducing tumor suppressor transcription factor TP53 as we reported previously (34). Moreover, we found that SRF deficient cardiomyocytes express higher levels of hepatocyte growth factor (HGF), which was attenuated by LIUS treatment in non-cancer cells. HGF plays a central role in metabolic disorders such as insulin resistance and in diabetes pathophysiology (75). Therefore, upregulation of SRF may serve as the suppressive mechanism underlying attenuation of HGF seen in LIUS treated non-cancer cells. Taken together, our results have demonstrated that modulation of transcription factors such as TP-53 and SRF-1 may play a role in altered cell death regulator gene expression seen in LIUS-treated cancer cells and non-cancer cells, respectively.

**Table 7:** LIUS modulate the expression of cell death regulators potentially via transcription factors TP53-, and SRF-, mediated pathways in cancer cells and non-cancer cells respectively (Increased or decreased fold changes which <1.5 was not indicated).

		**Non-carcinoma**	**Carcinoma**	**Non-carcinoma**
	**GEO ID**	**GSE28546**	**GSE90**	**GSE3181**
	**Organism**	**Homo sapiens**	**Homo sapiens**	**Mus musculus**
	**Cell/Tissue**	**Mesenchymal Stem Cells**	**Colorectal carcinoma-derived cell lines**	**Cardiomyocyte**
	**Treatment**	**p53 knock-down**	**p53 knock-out**	**SRF knockout**
LIUS-upregulated cell death regulators in cancer cells (13)	BOK, CASP10, CYLD, DPP4, EZR, ATG3, ATG16L1, VAMP1, CXCL10, ANXA1, FPR1, PANX1			
	**TP53**	−21.752	−2.941	
LIUS-downregulated gene in carcinoma cells (12)	HK2, CASP5, TLR7, MAP2K5, CDC25C, TTK, SRC, PDGFRA, ITGB1, ITGB3			
	CD47	3.660		
	ATM	4.252		
LIUS-upregulated gene in non-carcinoma cells (9)	MYC, NT5E, BAG6, NUMA1, IREB2, PTK2, PDGFRA, AKT3			
	SRF			−5.315
LIUS-downregulated gene in non-carcinoma cells (31)	BIRC5, BIK, CDK1, E2F1, E2F8, CCND1, GCLC, PYCARD, NLRP3, IL18, IL1B, IFNGR1, TLR7, MAP2K7, ATG10, ENTPD1, TLR2, MYT1, BUB1, BUB1B, TTK, KDR, ITGA6, TLR4, TRADD			
	CASP1	7.056		
	CASP4	3.559		
	CD47	3.660		
	ATM	4.252		
	HGF			1.678
	BCL2	3.793		

*The red highlights indicate increased fold changes, and green highlights indicate decreased fold changes*.

### Thermal Effects and Osmotic Shear Stress Associated With LIUS May Promote Gene Expression Changes of the Cell Death Regulatome in Cancer Cells and Non-cancer Cells

**Figure 2:**
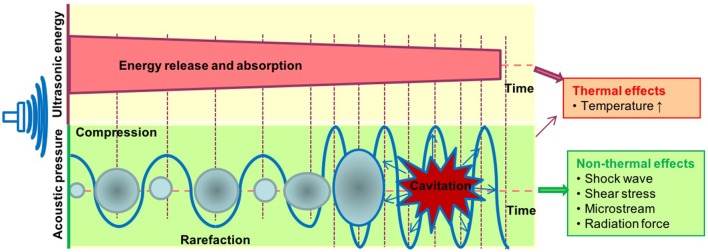
The biological effects exerted by ultrasound therapy includes thermal and non-thermal effects. The thermal effects of ultrasound that arise from the absorption of ultrasonic energy; and creation of heat depend on ultrasound exposure parameters, tissue properties, and beam configuration. Cavitation, acoustic radiation force, radiation torque, acoustic streaming, shock wave, and shear stress are considered non-thermal effects of ultrasound. Cavitation is perhaps the most widely studied biological effect and is described as the formation and oscillation of a gas bubble. In addition, the oscillation of the bubble can also result in heat generation.

It is well-accepted that the therapeutic applications of ultrasound depend on the propagation of ultrasound waves through tissues to produce biological effects (Figure 2). The biological effects of ultrasound are separated into thermal and non-thermal effects. The thermal effects of ultrasound that arise from the absorption of ultrasonic energy and creation of heat depend on ultrasound exposure parameters, tissue properties and beam configuration. Cavitation, acoustic radiation force, radiation torque, acoustic streaming, shock wave and shear stress are considered non-thermal effects of ultrasound (7) although there are some different opinions in classification of cavitation (7, 76, 77). LIUS is a form of ultrasound that delivered at a much lower intensity (<3 W/cm^2^) than high intensity focus ultrasound, and it has been considered as removed thermal component or minimal thermal effects due to its low intensity mode (78, 79). However, some publications have reported an increment of temperature by approximately 3 to 4°C after LIUS treatment (80), meaning that thermal effect is inevitable during LIUS treatment. We also examined the expressions of heat shock proteins in the three LIUS-treated microarrays and found that DNAJ (HSP40) heat shock protein (GAK) is upregulated (6.78-folds, *p* = 0.02) in one of the microarray datasets (human lymphoma, GSE10212) but is not significantly modulated in other two LIUS-treated microarray datasets. These results suggest that LIUS treatment induces the upregulation of heat shock protein gene and potential thermal stress; and that the differences in the expressions of GAK in three microarrays may be due to the potential differences of used LIUS methods and parameters as well as cell types (Table 1). We then hypothesized that thermal effects partially underlie the LIUS induced modulation of the cell death regulatome. To examine this hypothesis, we analyzed the microarray datasets conducted on cancer cells and non-cancer cells treated with mild hyperthermia (Table 1).

As shown in Table 8, mild hyperthermia induced upregulation of 27 out of 299 cell death regulators and downregulated 15 out of 299 cell death regulators in cancer cells. In addition, mild hyperthermia induced upregulation of 18 out of 299 cell death regulators and downregulated 7 out of 299 cell death regulators in non-cancer cells (Table 8). Interestingly, we found that similar to our observation in LIUS treated cancer cells, mild hyperthermia induced the expression of transcription factor TP-53 in cancer cells. Additionally, we observed that the expression of cell death regulators such as CYLD, EZR, VAMP1, ANAX1, and FPR1 was increased in mild hyperthermia treated cancer cells, which was similar to LIUS treated cancer cells. Further, we observed that expression of TLR7, MAP2K7, and SRC were downregulated in both data sets. Therefore, there is a possibility that mild hyperthermia associated with LIUS may regulate the expression of a few cell death regulators in cancer cells. Also, upregulation of the transcription factor TP-53 may occur due to mild hyperthermia associated with LIUS in cancer cells. Similarly, we observed that the expression of GCLC, NLRP3 and CD47 are downregulated in both groups of non-cancer cells treated with either LIUS or mild hyperthermia.

**Table 8:** Thermal effects and osmotic shear stress associated with LIUS may promote cell death regulator gene expression changes in cancer cells and non-cancer cells.

	**Mild hyperthermia in cancer cells**		**Mild hyperthermia in non-cancer cells**		**Oscillatory shear stress in non-cancer cells**	
	**Gene**	**Fold change**	**Gene**	**Fold change**	**Gene**	**Fold change**
Upregulate	BAG3	21.791	BAG6	8.983	PMAIP1	1.913
	PMAIP1	1.95	E2F5	14.03	TFRC	2.22
	CASP7	1.607	MAPK14	2.556	ENTPD1	1.568
	CDKN1A	3.834	YWHAZ	1.901	PIK3R1	2.099
	E2F5	1.999	GLS2	2.307		
	SLC25A4	2.011	KRAS	6.129		
	CYLD	1.708	MAP2K6	16.263		
	HSPB1	4.717	BECN1	6.343		
	CASP1	2.183	ENTPD1	24.619		
	IL1B	1.919	VAMP1	2.751		
	EZR	2.265	MYT1	7.391		
	MPO	9.341	PIK3CD	6.599		
	VAMP1	4.001	FASLG	3.046		
	SNAP25	1.797	TNFRSF25	6.836		
	NT5E	8.598	TLR3	27.497		
	ANXA1	1.655	ATG7	1.799		
	FPR1	9.078	MAP1LC3B2	3.834		
	CDC25A	1.534	BCL2	9.434		
	BUB1	2.325				
	PLK2	10.193				
	AURKC	2.783				
	EGFR	1.77				
	ITGB1	10.408				
	ITGB3	3.54				
	FAS	2.16				
	ATG5	3.488				
	TP53	2.79				
	Gene number	27	Gene number	18	Gene number	4
Downregulate	CAD	−1.729	CASP9	−7.155	BIRC5	−2.915
	TFRC	−1.747	GCLC	−3.16	CDK1	−2.364
	NLRP1	−3.732	NLRC4	−4.517	E2F8	−1.663
	IFNGR1	−1.948	NLRP3	−6.068	DPP4	−4.136
	TLR7	−5.953	MKL2	−7.689	FANCD2	−2.138
	MKL2	−1.704	CD47	−7.587	CASP1	−1.838
	KRAS	−1.742	P2RX7	−3.698	DDX58	−2.423
	MAP2K7	−3.807			DRAM1	−1.509
	MTOR	−1.616			ULK2	−1.561
	ENTPD1	−3.162			CXCL10	−2.419
	CCNE1	−1.91			AURKA	−2.02
	BUB1B	−1.741			BUB1	−3.444
	SRC	−1.916			BUB1B	−2.9
	HGF	−8.403			CDC20	−2.144
	BCL2	−1.536			CDC25B	−1.713
					CDC25C	−1.514
					CENPE	−2.47
					PLK1	−2.588
					TTK	−2.641
					BCL2L11	−1.728
					ITGB3	−2.154
					TLR3	−2.346
	Gene number	15	Gene number	7	Gene number	22

**Figure 3:**
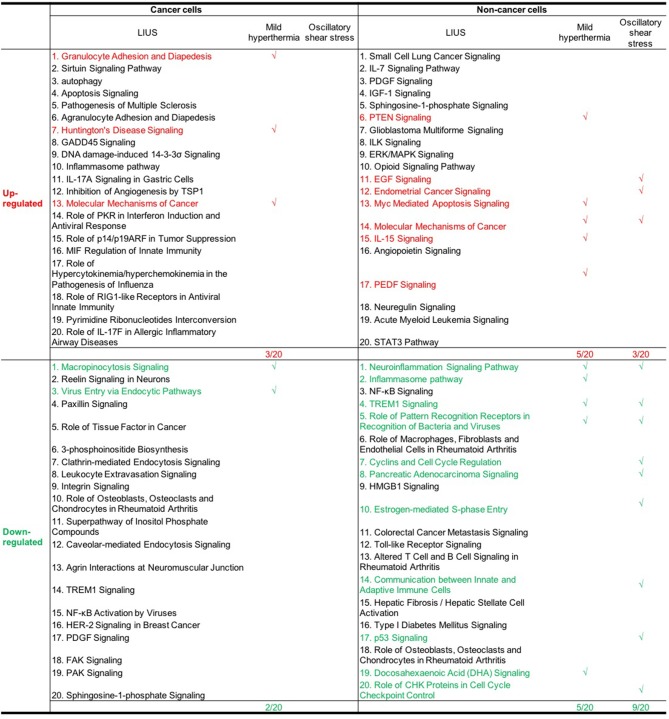
The common signaling pathways that were shared by cell death regulators that were modulated by mild hyperthermia, OSS, and LIUS. The top 20 pathways that are modulated by mild hyperthermia and oscillatory shear stress in cancer cells and non-cancer cells were shown in Figures S1A–F.

Furthermore, we analyzed whether there are similarities between the signaling pathways that are regulated by genes that were modulated by LIUS and mild hyperthermia. Our analysis revealed that there are three common signaling pathways that were shared by cell death regulator genes that were upregulated by LIUS and mild hyperthermia in cancer cells (Figure 3). These cell signaling pathways are granulocyte adhesion and diapedesis, Huntington's disease signaling, and molecular mechanisms of cancer. In addition, the down regulated cell death regulators in cancer cells treated by LIUS or mild hyperthermia shared 2 signaling pathways such as macropinocytosis signaling and virus entry via endocytotic pathway (Figure 3). Despite the fact that there are no common upregulated genes attributed to both groups, in non-cancer cells cell death regulators that are modulated by LIUS and mild hyperthermia shared five signaling pathways. These are PTEN signaling, Myc mediated apoptosis signaling, molecular mechanisms of cancer, IL-15 signaling, and PEDF signaling. Furthermore, cell death regulators that were downregulated by LIUS treatment or mild hyperthermia in non-cancer cells affected four signaling pathways including neuroinflammation signaling pathway, inflammasome pathway, TREM1 signaling, role of pattern recognition receptors in recognition of bacteria and viruses, and DHA signaling regulation. Taken together, these results suggest that unlike what was reported previously, thermal effect may play an indispensable role for the LIUS induced modulation of cell death regulators.

We also hypothesized that non-thermal effects such as oscillatory shear stress (OSS) partially underlie LIUS induced modulation of cell death regulators. To examine this hypothesis, we utilized microarray datasets performed on non-cancer cells treated with OSS (Table 1). Of note, we did not find any microarray datasets that were conducted on cancer cells treated with OSS. OSS-induced upregulation of 4 out of 299 cell death regulators involved in four cell death types such as apoptosis, ferroptosis, ICD and anoikis and downregulated 22 out of 299 cell death regulators in non-cancer cells involved in ten out of 13 cell death types (Table 8). These results suggest that similar to LIUS, OSS has the tendency to downregulate the expression of cell death regulators in non-cancer cells. Nevertheless, we could not find a similarity between the genes that were upregulated by OSS and LIUS treatment in non-cancer cells. However, 5 genes including BIRC5, CDK1, E2F8, CASP1, and BUB1 were downregulated in both datasets.

Similar to LIUS treatment, we observed that OSS treatment did not affect the expression of cell death regulators involved in three cell death types such as MPT-driven necrosis, parthanatos, and NETotic cell death. Despite the fact the OSS and LIUS treatment did not modulate similar set of genes in non-cancer cells, we found that the cell death regulator genes that were upregulated by both treatments share three signaling pathways (Figure 3). These three pathways are EGF signaling, endometrial cancer signaling, and molecular mechanisms of cancer. Interestingly, the cell death regulators, that are down-regulated by LIUS or OSS treatment in non-cancer cells, downregulate nine common pathways. These signaling pathways are TREM1 signaling, role of pattern recognition receptors in recognition of bacteria and viruses, cyclins and cell cycle regulation, pancreatic adenocancer signaling, estrogen-mediated S-phase entry, communication between innate and adaptive immune cells, p53 signaling, and role of CHK proteins in cell cycle checkpoint control. Taken together, these results suggest that non-thermal effects of LIUS such as OSS may be responsible for inducing biological effects observed in non-cancer cells.

### LIUS May Cause Changes in the Oxidative Environment of the Cancer and Non-cancer Cells by Regulating Genes That Are Responsible for Transcribing ROS Generating and Antioxidant Enzymes

Oxidative stress is well-known to be involved in a wide variety of human diseases including cardiovascular diseases, inflammatory disorders, immune system dysfunction, diabetes, cancer, aging and neurodegenerative disorders (81–84). Low grade reactive oxygen species (ROS) generation helps to mediate signaling that maintain the homeostasis of the cells, however, excess production of ROS can be detrimental to the cells by activating cell death pathways. Previously, it was shown that LIUS exerts cytoprotective effects against oxidative injury in human aortic endothelial cells and retinal pigment epithelium cells (85, 86). In contrast, LIUS exerts cancer suppressing effects by inducing ROS generation, which result in mitochondrial damage and subsequent cell death (44, 47, 87, 88). Therefore, we analyzed whether LIUS treatment exerts any expression changes of genes in our datasets that has the potential to regulate oxidative stress (Table 9). Interestingly, we observed that LIUS treatment in cancer cells induced the expression of genes that regulate ROS generation while downregulating the expression of GPX3 that has the potential to exert antioxidant effects. Therefore, this suggests that LIUS may cause changes in the redox status in cancer cells. Further, we observed that LIUS has the tendency to induce antioxidant effects in non-cancer cells by attenuating the gene expression of NOS2 and NOS3, which promote ROS generation, while promoting the expression of antioxidant genes GPX3 and GPX7. It is well-known that ROS can indeed impact the cell viability by affecting activity of caspases, mitochondrial function and activating apoptotic pathways depending on the cellular contexts (89). Therefore, it is a possibility that LIUS-mediated changes in the redox status of the cancer and non-cancer cells may have an impact on cell death regulator gene expression leading to activation of various cell death pathways.

**Table 9:** LIUS promotes the expression of reactive oxygen species (ROS) generating enzymes in cancer cells, and enhances the expression of antioxidant enzymes in non-cancer cells (Increased or decreased fold changes which <1.5 was not indicated).

	**Treatment**	**LIUS**			**Mild hyperthermia**		**Oscillatory**
	**Disease**	**Cancer**	**Non-cancer**		**Cancer**	**Non-cancer**	**Non-cancer**
	**Cell**	**Lymphoma U937 cells**	**MC3T3-E1 preosteoblast cells**	**Bone marrow cells**	**Lymphoma U937 cells**	**Fibroblast OUMS-36 cells**	**Human lymphatic endothelial cells**
	**GEO ID**	**GSE10212**	**GSE45487**	**GSE70662**	**GSE10043**	**GSE39178**	**GSE60512**
ROS generating enzyme	XDH						
	NOX1	3.047			2.194	2.792	
	NOX3	1.697					
	NOX4						
	NOX5					2.340	
	NOS1				−1.824		
	NOS2	4.370		−1.640	−3.747	3.146	
	NOS3			−2.150			1.988
	MPO				9.341		
Antioxidant enzymes	GPX1						
	GPX2						
	GPX3	3.317		1.996			2.768
	GPX4						
	GPX5				1.553		
	GPX6						
	GPX7			1.614	−3.435		1.545
	GPX8						
	GSR						1.854
	CAT				−8.137		
	SOD1						
	SOD2						−3.878
	SOD3						

*The gene list of chromosome conformation regulators was extracted from related report (PMID: 28698768), detailed information (see Table S16). The red highlights indicate increased fold changes, and green highlights indicate decreased fold changes*.

Further, we analyzed whether mild hyperthermia and OSS has the potential to change the redox status by regulating ROS generating and antioxidant genes. We observed that there were changes in genes that regulate ROS generation and exert antioxidant effects in cancer cells; and to our surprise, we observed an induction of ROS generating genes in non-cancer cells with treated with mild hyperthermia. However, our data revealed that OSS has the tendency to exert anti-inflammatory effects in non-cancer cells, similar to our observation in LIUS treated non-cancer cells. Therefore, it can be postulated that OSS may impact the LIUS-mediated redox status in non-cancer cells.

### LIUS May Modulate Chromatin Long Range Interactions to Regulate Gene Expression in Cancer Cells and Non-cancer Cells

The results from this study, our previous study (2) and others' reports indicated that LIUS regulates gene expression presumably at transcription levels. Our recent further reports showed that histone modification enzymes are significantly modulated in response to disease risk factor stimulations (90); that IL-35 suppresses endothelial cell activation by inhibiting mitochondrial reactive oxygen species-mediated site specific acetylation of histone 3 lysine 14 (91); and that DNA damage factors and DNA repair factors serve as an integrated sensor and cell fate determining machinery for all the intracellular stresses and dangers (92). Our reports suggest that various nuclear programs control gene expression responses to endogenous and exogenous DAMPs and other stimuli including LIUS.

We hypothesized that newly characterized chromatin long range interactions (CLRI) differentially regulate the gene promoters to differentiate LIUS-modulated gene expression in cancer cells vs. non-cancer cells. To test this hypothesis with respect to LIUS effects in modulating chromatin remodeling, we examined the expression changes of chromatin insulator-binding factors such as CTCF and RAD21 and other promoter-binding factors and non-promoter binding factors in LIUS-treated cancer cells and non-cancer cells (93). As shown in Table 10, LIUS did not change the expression of two insulator-binding factors, 16 promoter-binding factors but changed the expression of one of six non-promoter binding factors in cancer cells. In addition, LIUS changed the expressions of two out of 16 promoter binding factors in non-cancer cells.

**Table 10:** LIUS modulated the expression of chromosome conformation regulators in carcinoma cells and non-carcinoma cells.

	**Treatment**	**LIUS**		
	**GEO ID**	**GSE10212**	**GSE45487**	**GSE70662**
**Protein types**	**Cell types**	**Carcinoma**	**Non-carcinoma**	**Non-carcinoma**
Insulator-binding factors	CTCF			
	RAD21			
Promoter-binding factors	POLR3A			
	POLR3B			
	POLR3C			
	POLR3D			2.054
	POLR3E			
	POLR3F			2.178
	POLR3G			
	POLR3H			
	GTF3C1			
	GTF3C2			
	GTF3C3			
	GTF3C4			
	GTF3C5			
	BRF1			
	BDP1			
	E2F4			
Non-promoter-binding factors	JUN			
	GATA1			
	GATA2	1.627		
	SMARCB1			
	SMARCA4			
	SIRT6			
	Up	1/24	0/24	2/24
	Down	0/24	0/24	0/24

*LIUS upregulated the non-promoter-binding factors in carcinoma cells, but upregulated the promoter-binding factors in non-carcinoma cells. The gene list of chromosome conformation regulators was extracted from related report (PMID: 22675074), detailed information (see Table S17)*.

**Figure 4:**
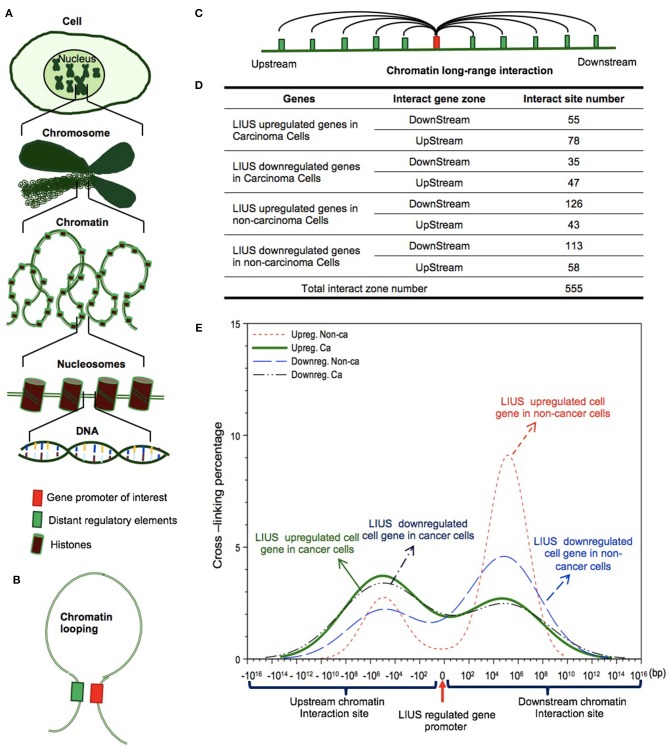
LIUS may modulate chromatin long-range interactions to regulate gene expression in cancer cells and non-cancer cells. **(A)** Chromatin is a whole structure of complex DNA and proteins, it forms the chromosomes of eukaryotic organisms and is packaged inside the nucleus. Nucleosome is a basic unit of chromatin, consisting of a length of DNA coiled around a core of histones. **(B)** Chromatin looping makes gene promoter and distal regulatory elements are in close proximity, and possibly interact to each other. **(C)** Long-range interactions allow communication between promoters and different distant regulatory elements. **(D)** The interacting sites mostly located in upstream zone of promoters in cancer cells, but higher percentages located in downstream zone of promoters in non-cancer cells. **(E)** The long-range interaction sites of LIUS regulated genes in cancer cells mostly located between −10^7^ and −10^5^ upstream, but in non-cancer cells, the long-range interaction sites of LIUS regulated genes were concentrated between 10^5^ and 10^7^ bp downstream.

We further hypothesized that the differential gene expression seen between LIUS treated cancer cells and non-cancer cells were due to differences in the CLRIs of the modulated genes Figures 4A–C. Therefore, to analyze this, we obtained chromatin long-range interaction data for all the significantly modulated cell death regulator genes from the 4DGenome database. This is a well-accepted database, which contains information on a huge collection of 4,433,071 experimentally-derived chromatin long-range interactions (66). We then calculated the distances between 555 interacting sites (Figure 4D) with respect to LIUS-modulated gene promoters. If the LIUS-modulated gene promoter was located downstream of its long-range interaction partner, we designated the CLRI as negative. If the CLRI site is located downstream of the target gene promoter, we designated the interaction as positive. The two-sample Kolmogorov-Smirnov test of the chromatin long-range interaction distances between gene promoters corresponding to LIUS downregulated and upregulated genes indicated some significant differences between the two distance distributions (*p* < 0.001) (Figure 4E).

Our data indicated that the majority of CLRI sites for cell death regulator genes that were modulated by LIUS treatment were concentrated between −10^7^ and −10^5^ base pairs (bp) upstream or 10^5^ and 10^7^ bp downstream in cancer cells. Interestingly, we also found that the CLRI sites of the genes that were upregulated by LIUS were spanned between a narrower range compared to that of the downregulated genes in cancer cells (Figure 4E). However, in non-cancer cells, most of the CLRI sites of cell death regulator genes modulated by LIUS were located downstream of the target genes. Similar to the observation seen in genes modulated by LIUS in cancer cells, the CLRIs of upregulated cell death regulators spanned across a comparatively narrower region than that of downregulated genes in LIUS treated non-cancer cells. Of note, LIUS induced upregulation of RNA polymerase III subunit D (POLR3D) and RNA polymerase III subunit F (POLR3F), two out of 16 promoter binding factors in non-cancer cells in Table 10, are associated LIUS-induced higher concentrations of CLRISs. Of note, a common feature of the CLRISs with LIUS modulated genes in cancer cells is associated with LIUS upregulation of GATA2 in Table 10.

Future experiments will be needed to verify these interesting associations between CLRI sites and the genes that were modulated by LIUS treatment in both cancer and non-cancer cells. Since the 4DGenome database contains the experimental data derived from human non-aortic endothelial cells (66), the future work will be needed to use circular chromosome conformation capture sequencing (4C-Seq) to examine LIUS-treated cancer cells and non-cancer cells to map the specific upstream interaction sites for modulation of cell death regulator expression in cancer cells and non-cancer cells. Taken together, our results have demonstrated for the first time that, LIUS induced a differential gene expression pattern in the cell death regulatome in cancer cells and non-cancer cells, and that these genes have unique CLRI sites. Therefore, our results may suggest that optimal CLRISs may serve as new therapeutic targets in the future to enhance LIUS-mediated cancer cell suppression and LIUS anti-inflammatory functions in non-cancer cells.

## Discussion

Therapeutic applications of ultrasound in addition to its use in diagnosis, have been accepted to be clinically beneficial. These benefits include that LIUS functions in suppressing cancers via inducing cell death pathways (Table 4). As we pointed out previously (2), the anti-inflammatory effects are responsible for inducing the clinical benefits mediated by LIUS in non-cancer cells (94–96). However, the molecular mechanisms underlying the anti-cancer cell functions and anti-inflammatory effects of LIUS remain poorly defined. Determination of the novel molecular mechanisms underlying the anti-cancer cell functions and anti-inflammatory properties of LIUS in non-cancer cells would significantly improve our understanding on this important issue, and allow for the improvement of LIUS-based therapeutics.

To fill in this important knowledge gap, in this study, we used cutting-edged molecular database mining approaches that we pioneered in 2004 (90, 97–99). Our data analyses have made for the first time the following significant findings: (1) LIUS differentially regulates cell death regulatome gene expression in cancer cells and in non-cancer cells. However, in non-cancer cells, LIUS treatment has the tendency to downregulate the expression of cell death regulators more. Most of the cell death regulator genes downregulated by LIUS in non-cancer cells are responsible for mediating inflammatory signaling pathways; (2) LIUS inhibits the expression of several inflammatory cell death regulators potentially via TP53-, and SRF-, mediated pathways in cancer cells and non-cancer cells, respectively; (3) Thermal effects and osmotic shear stress associated with LIUS may play a role in altering the cell death regulator expression patterns; (4) LIUS has the tendency to induce antioxidant effects specifically in non-cancer cells; and (5) The genes that were modulated in cancer cells by LIUS have unique CLRI patterns, different from that of non-cancer cells.

It is not clear how LIUS exposure may transmit signals to the nucleus to modulate the gene expression in both cancer and non-cancer cells. Previously, it was shown that LIUS can overstretch the cell membrane and cause reparable submicron pore formation (100). This phenomenon is called sonoporation. Such effects may lead to disruption of cytoskeleton in tandem because this network of subcellular filaments is physically interconnected with the plasma membrane (101). Therefore, sonoporation associated with LIUS may be responsible for inducing important biological effects in cells.

In addition, ultrasound at low diagnostic power can cause stable oscillations of the microbubbles, resulting in a transient increase in membrane permeability for Ca^2+^ (102, 103). We previously reported that LIUS may make use of natural membrane vesicles as small as exosomes which are derived from immunosuppressor cells to fulfill its anti-inflammatory effects by upregulating the expression of extracellular vesicle/exosome biogenesis mediators and docking mediators (2). Taken together, all these factors may profoundly affect cellular sensors that can activate various downstream signaling pathways in tissues exposed to LIUS. However, our findings suggest that cancer cells and non-cancer cells may use distinct signaling mechanisms to activate downstream targets when exposed to LIUS. Our analysis revealed that LIUS can activate more of antioxidant effects in non-cancer cells compared to cancer cells. Such changes in redox status of the cellular environment may lead to activation of sensors that may produce distinct gene expression patterns in non-cancer cells relative to cancer cells exposed to LIUS. Our data indicated that different cell death regulatory transcription factors are induced in cancer cells and non-cancer cells treated with LIUS. For an example, we observed that TP53 and SRF-1 genes were induced in LIUS treated cancer cells and non-cancer cells, respectively. Therefore, it can be hypothesized that LIUS produces differential biological responses in different cellular contexts by activating distinct transcription factors, thus activating specific gene expression patterns. Most interestingly, we observed unique patterns in CLRI sites in genes modulated by LIUS in cancer cells and non-cancer cells. Therefore, LIUS may have the ability to modulate CLRI sites in cancer cells and non-cancer cells, leading to distinct gene expression patterns. Also, mild hyperthermia and OSS associated with LIUS may also play a role in generating distinct transcriptome profiles depending on the cellular context.

**Figure 5:**
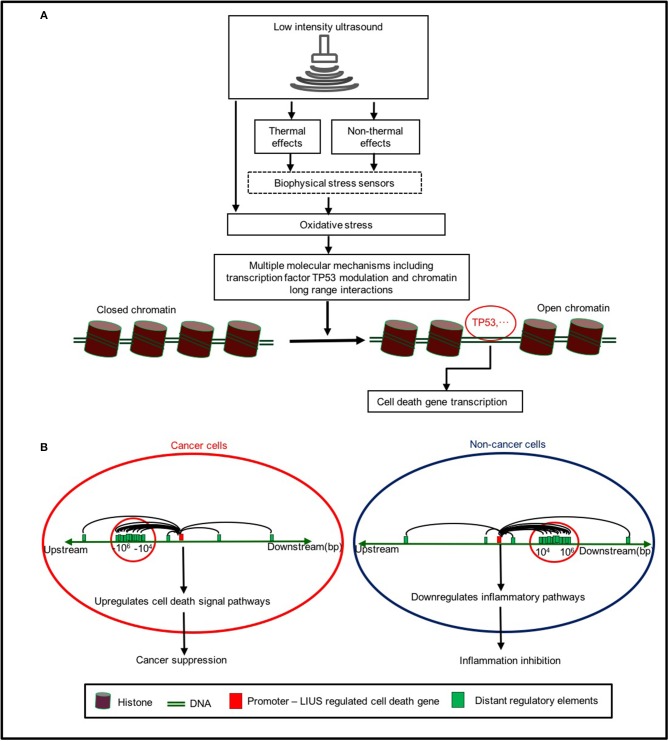
A new working model on LIUS mediated cancer-suppressing and anti-inflammatory mechanisms. **(A)** LIUS produces thermal and non-thermal effects which induce cell death gene expression potentially via transcription factors TP53-, and SRF-, mediated pathways. **(B)** LIUS may modulate chromatin long range interactions to differentially regulate cell death gene expression in cancer cells and non-cancer cells. Upstream chromatin long-range interaction sites (CLRISs) are more favorable than downstream CLRISs for LIUS modulation of cell death regulator expressions in cancer cells; and in contrast, downstream CLRISs play more important roles than upstream CLRISs for LIUS downregulation of inflammatory pathways in non-cancer cells.

Based on our findings, we propose a new working model on LIUS-mediated cancer-suppressing and anti-inflammatory mechanisms as shown in Figure 5. Our new model integrates the follow findings: *First*, LIUS induces cell death gene expression potentially via transcription factors TP53-, and SRF-, mediated pathways. *Second*, the therapeutic applications of LIUS may depend on the propagation of ultrasound waves through tissues to produce thermal and non-thermal mechanic effects. *Third*, LIUS may modulate chromatin long-range interactions to differentially regulate the cell death regulatome gene expression in cancer cells and non-cancer cells. Upstream chromatin long-range interaction sites (CLRISs) are more favorable than downstream CLRISs for LIUS modulation of cell death regulator expression in cancer cells; and in contrast, downstream CLRISs play more important roles than upstream CLRISs for LIUS downregulation of inflammatory pathways in non-cancer cells.

One limitation of the current study is the unavailability of biological data obtained from LIUS-treated patients' biopsies. We acknowledge that carefully designed *in-vitro* and *in-vivo* experimental models will be needed to further verify the LIUS mediated cancer-suppressing and anti-inflammatory mechanisms we report here. These experimental models will enable to consolidate the efficacy of LIUS in various pathological conditions as well. However, our analyses provide a significant insight in to LIUS-mediated modulation of the cell death regulatome via newly-defined nuclear programs to induce cell death in cancer cells and downregulate more inflammatory pathways in non-cancer cells. Once again (2), our findings provide molecular readouts that can be used to determine optimal ultrasound intensity and duration, and will provide guidance for the development of the future LIUS therapeutics for cancers, inflammations, tissue regeneration, and tissue repair.

## Conclusions

Our report allows us to propose a new molecular working model for LIUS therapies for the treatment of cancers and inflammation: *First*, LIUS differentially upregulates cell death regulators in cancer cells, and downregulates inflammatory pathways in non-cancer cells potentially via transcription factors TP53-, and SRF-, mediated pathways; *Second*, the therapeutic applications of LIUS may depend on the propagation of ultrasound waves through tissues to produce thermal and non-thermal mechanic effects; *Third*, LIUS may modulate chromatin long-range interactions to differentially regulate cell death gene expressions in cancer cells and non-cancer cells. Our findings provide a significant insight in LIUS-mediated modulation of the expressions of cell death regulators via newly-defined nuclear programs to induce more cell death in cancer cells and downregulate more inflammatory pathways in non-cancer cells. Also, our findings provide molecular readouts that can be used to determine optimal ultrasound intensity and duration; and will provide guidance for the development of the future LIUS therapeutics for cancers, inflammation, tissue regeneration, and tissue repair.

## Author Contributions

JW and BL carried out the data gathering, data analysis, and prepared tables and figures. GN, QY, YSu, YL, YSh, DY, WY, RC, HF, HZ, WS, SW, CZ, YL, EC, and HW aided with analysis of the data. XY supervised the experimental design, data analysis, and manuscript writing. All authors read and approved the final manuscript.

### Conflict of Interest Statement

The authors declare that the research was conducted in the absence of any commercial or financial relationships that could be construed as a potential conflict of interest.

## References

[1] de Castro CC Gondim Gomes CB Martins MR de Sousa JC Magalhaes SM Pinheiro RF. Tissue doppler echocardiography detects preclinical markers of cardiac lesion in MDS patients. J Hematol Oncol. (2012) 5:30. 10.1186/1756-8722-5-30 22709732 PMC3418154

[2] Yang Q Nanayakkara GK Drummer C Sun Y Johnson C Cueto R. Low-intensity ultrasound-induced anti-inflammatory effects are mediated by several new mechanisms including gene induction, immunosuppressor cell promotion, and enhancement of exosome biogenesis and docking. Front Physiol. (2017) 8:818. 10.3389/fphys.2017.00818 29109687 PMC5660123

[3] Schroeder A Kost J Barenholz Y. Ultrasound, liposomes, and drug delivery: principles for using ultrasound to control the release of drugs from liposomes. Chem Phys Lipids. (2009) 162:1–16. 10.1016/j.chemphyslip.2009.08.003 19703435

[4] Sheikov N McDannold N Vykhodtseva N Jolesz F Hynynen K. Cellular mechanisms of the blood-brain barrier opening induced by ultrasound in presence of microbubbles. Ultrasound Med Biol. (2004) 30:979–89. 10.1016/j.ultrasmedbio.2004.04.010 15313330

[5] Forbes MM O'Brien WD Jr. Development of a theoretical model describing sonoporation activity of cells exposed to ultrasound in the presence of contrast agents. J Acoust Soc Am. (2012) 131:2723–9. 10.1121/1.3687535 22501051 PMC3339499

[6] Kravchenko IA Kobernik AA Aleksandrova AI Prystupa BV Lepikh Ia I shegur PA. [Anti-inflammatory effect of therapeutic and low-frequency ultrasound on a rat model of inflammatory process]. Biofizika. (2013) 58:540–6. 10.1134/S0006350913030081 24159825

[7] Izadifar Z Babyn P Chapman D. Mechanical and biological effects of ultrasound: a review of present knowledge. Ultrasound Med Biol. (2017) 43:1085–104. 10.1016/j.ultrasmedbio.2017.01.023 28342566

[8] Kulke MH Bendell J Kvols L Picus J Pommier R Yao J. Evolving diagnostic and treatment strategies for pancreatic neuroendocrine tumors. J Hematol Oncol. (2011) 4:29. 10.1186/1756-8722-4-29 21672194 PMC3128039

[9] Wang J Saukel GW Garberoglio CA Srikureja W Hsueh CT. Pathological complete response after neoadjuvant chemotherapy with trastuzumab-containing regimen in gastric cancer: a case report. J Hematol Oncol. (2010) 3:31. 10.1186/1756-8722-3-31 20828403 PMC2944145

[10] Copelan A Hartman J Chehab M Venkatesan AM. High-intensity focused ultrasound: current status for image-guided therapy. Semin Interv Radiol. (2015) 32:398–415. 10.1055/s-0035-1564793 26622104 PMC4640913

[11] Wood AK Sehgal CM. A review of low-intensity ultrasound for cancer therapy. Ultrasound Med Biol. (2015) 41:905–28. 10.1016/j.ultrasmedbio.2014.11.019 25728459 PMC4362523

[12] McHale AP Callan JF Nomikou N Fowley C Callan B. Sonodynamic therapy: concept, mechanism and application to cancer treatment. Adv Exp Med Biol. (2016) 880:429–50. 10.1007/978-3-319-22536-4_22 26486350

[13] Mijajlovic MD Pavlovic AM Covickovic-Sternic N. Is sonothrombolysis an effective stroke treatment? J Ultrasound Med. (2013) 32:1117–23. 10.7863/ultra.32.7.1117 23804334

[14] Roberts WW. Development and translation of histotripsy: current status and future directions. Curr Opin Urol. (2014) 24:104–10. 10.1097/MOU.0000000000000001 24231530 PMC3974592

[15] Friedmann DP. A review of the aesthetic treatment of abdominal subcutaneous adipose tissue: background, implications, and therapeutic options. Dermatol Surg. (2015) 41:18–34. 10.1097/DSS.0000000000000209 25521101

[16] Padilla F Puts R Vico L Raum K. Stimulation of bone repair with ultrasound: a review of the possible mechanic effects. Ultrasonics. (2014) 54:1125–45. 10.1016/j.ultras.2014.01.004 24507669

[17] Rutjes AW Nuesch E Sterchi R Juni P Therapeutic ultrasound for osteoarthritis of the knee or hip. Cochrane Database Syst Rev. (2010) 20:CD003132 10.1002/14651858.CD003132.pub2 20091539

[18] Page MJ O'Connor D Pitt V Massy-Westropp N. Therapeutic ultrasound for carpal tunnel syndrome. Cochrane Database Syst Rev. (2012) 1:CD009601. 10.1002/14651858.CD009601 22259004

[19] Sato M Kuroda S Mansjur KQ Khaliunaa G Nagata K Horiuchi S. Low-intensity pulsed ultrasound rescues insufficient salivary secretion in autoimmune sialadenitis. Arthritis Res Ther. (2015) 17:278. 10.1186/s13075-015-0798-8 26445930 PMC4596462

[20] Mele L Vitiello PP Tirino V Paino F De Rosa A Liccardo D. Changing paradigms in cranio-facial regeneration: current and new strategies for the activation of endogenous stem cells. Front Physiol. (2016) 7:62. 10.3389/fphys.2016.00062 26941656 PMC4764712

[21] Johns LD. Nonthermal effects of therapeutic ultrasound: the frequency resonance hypothesis. J Athl Train. (2002) 37:293–9. 16558674 PMC164359

[22] Nagao M Tanabe N Manaka S Naito M Sekino J Takayama T. LIPUS suppressed LPS-induced IL-1alpha through the inhibition of NF-kappaB nuclear translocation via AT1-PLCbeta pathway in MC3T3-E1 cells. J Cell Physiol. (2017) 232:3337–46. 10.1002/jcp.25777 28063227

[23] Hashish I Harvey W Harris M. Anti-inflammatory effects of ultrasound therapy: evidence for a major placebo effect. Br J Rheumatol. (1986) 25:77–81. 10.1093/rheumatology/25.1.77 2417648

[24] ElHag M Coghlan K Christmas P Harvey W Harris M. The anti-inflammatory effects of dexamethasone and therapeutic ultrasound in oral surgery. Br J Oral Maxillofac Surg. (1985) 23:17–23. 10.1016/0266-4356(85)90074-9 3156621

[25] Chung JI Barua S Choi BH Min BH Han HC Baik EJ. Anti-inflammatory effect of low intensity ultrasound (LIUS) on complete Freund's adjuvant-induced arthritis synovium. Osteoarthritis Cartilage. (2012) 20:314–22. 10.1016/j.joca.2012.01.005 22289897

[26] Bykov VJN Eriksson SE Bianchi J Wiman KG. Targeting mutant p53 for efficient cancer therapy. Nat Rev Cancer. (2018) 18:89–102. 10.1038/nrc.2017.109 29242642

[27] Soteriou D Fuchs Y. A matter of life and death: stem cell survival in tissue regeneration and tumour formation. Nat Rev Cancer. (2018) 18:187–201. 10.1038/nrc.2017.122 29348578

[28] Linkermann A Stockwell BR Krautwald S Anders HJ. Regulated cell death and inflammation: an auto-amplification loop causes organ failure. Nat Rev Immunol. (2014) 14:759–67. 10.1038/nri3743 25324125

[29] Croft M Siegel RM. Beyond TNF: TNF superfamily cytokines as targets for the treatment of rheumatic diseases. Nat Rev Rheumatol. (2017) 13:217–33. 10.1038/nrrheum.2017.22 28275260 PMC5486401

[30] Yang XF Weber GF Cantor H. A novel Bcl-x isoform connected to the T cell receptor regulates apoptosis in T cells. Immunity. (1997) 7:629–39. 10.1016/S1074-7613(00)80384-2 9390687 PMC3908546

[31] Yang Y Yang F Xiong Z Yan Y Wang X Nishino M. An N-terminal region of translationally controlled tumor protein is required for its antiapoptotic activity. Oncogene. (2005) 24:4778–88. 10.1038/sj.onc.1208666 15870695 PMC3901995

[32] Yang Y Xiong Z Zhang S Yan Y Nguyen J Ng B. Bcl-xL inhibits T-cell apoptosis induced by expression of SARS coronavirus E protein in the absence of growth factors. Biochem J. (2005) 392(Pt 1):135–43. 10.1042/BJ20050698 16048439 PMC1317672

[33] Yang XF. Factors regulating apoptosis and homeostasis of CD4+ CD25(high) FOXP3+ regulatory T cells are new therapeutic targets. Front Biosci. (2008) 13:1472–99. 10.2741/2775 17981643

[34] Xiong Z Song J Yan Y Huang Y Cowan A Wang H. Higher expression of Bax in regulatory T cells increases vascular inflammation. Front Biosci. (2008) 13:7143–55. 10.2741/3217 18508723 PMC2915779

[35] Xiong Z Yan Y Song J Fang P Yin Y Yang Y. Expression of TCTP antisense in CD25(high) regulatory T cells aggravates cuff-injured vascular inflammation. Atherosclerosis. (2009) 203:401–8. 10.1016/j.atherosclerosis.2008.07.041 18789801 PMC2695670

[36] Yan Y Xiong Z Zhang S Song J Huang Y Thornton AM. CD25high T cells with a prolonged survival inhibit development of diabetes. Int J Immunopathol Pharmacol. (2008) 21:767–80. 10.1177/039463200802100401 19144262 PMC3050009

[37] Yin Y Li X Sha X Xi H Li YF Shao Y. Early hyperlipidemia promotes endothelial activation via a caspase-1-sirtuin 1 pathway. Arterioscler Thromb Vasc Biol. (2015) 35:804–16. 10.1161/ATVBAHA.115.305282 25705917 PMC4376583

[38] Lopez-Pastrana J Ferrer LM Li YF Xiong X Xi H Cueto R. Inhibition of caspase-1 activation in endothelial cells improves angiogenesis: a novel therapeutic potential for ischemia. J Biol Chem. (2015) 290:17485–94. 10.1074/jbc.M115.641191 26037927 PMC4498083

[39] Li YF Huang X Li X Gong R Yin Y Nelson J. Caspase-1 mediates hyperlipidemia-weakened progenitor cell vessel repair. Front Biosci. (2016) 21:178–91. 10.2741/4383 26709768 PMC4693615

[40] Xi H Zhang Y Xu Y Yang WY Jiang X Sha X. Caspase-1 inflammasome activation mediates homocysteine-induced pyrop-apoptosis in endothelial cells. Circ Res. (2016) 118:1525–39. 10.1161/CIRCRESAHA.116.308501 27006445 PMC4867131

[41] Ferrer LM Monroy AM Lopez-Pastrana J Nanayakkara G Cueto R Li YF. Caspase-1 plays a critical role in accelerating chronic kidney disease-promoted neointimal hyperplasia in the carotid artery. J Cardiovasc Transl Res. (2016) 9:135–44. 10.1007/s12265-016-9683-3 26928596 PMC5131710

[42] Wang P Leung AW Xu C. Low-intensity ultrasound-induced cellular destruction and autophagy of nasopharyngeal carcinoma cells. Exp Ther Med. (2011) 2:849–52. 10.3892/etm.2011.317 22977587 PMC3440834

[43] Ye Q Meng C Shen Y Ji J Wang X Zhou S. Caveolin-1 mediates low-intensity ultrasound-induced apoptosis via downregulation of signal transducer and activator of transcription 3 phosphorylation in laryngeal carcinoma cells. Ultrasound Med Biol. (2016) 42:2253–60. 10.1016/j.ultrasmedbio.2016.04.017 27289429

[44] Feng Y Tian Z Wan M. Bioeffects of low-intensity ultrasound *in vitro*: apoptosis, protein profile alteration, and potential molecular mechanism. J Ultrasound Med. (2010) 29:963–74. 10.7863/jum.2010.29.6.963 20498470

[45] Feril LB Jr Kondo T Cui ZG Tabuchi Y Zhao QL Ando H. Apoptosis induced by the sonomechanical effects of low intensity pulsed ultrasound in a human leukemia cell line. Cancer Lett. (2005) 221:145–52. 10.1016/j.canlet.2004.08.034 15808400

[46] Ivone M Pappalettere C Watanabe A Tachibana K. Study of cellular response induced by low intensity ultrasound frequency sweep pattern on myelomonocytic lymphoma U937 cells. J Ultrasound. (2016) 19:167–74. 10.1007/s40477-016-0199-0 27635161 PMC5005207

[47] Li Y Zhou Q Hu Z Yang B Li Q Wang J. 5-Aminolevulinic acid-based sonodynamic therapy induces the apoptosis of osteosarcoma in mice. PloS ONE. (2015) 10:e0132074. 10.1371/journal.pone.0132074 26161801 PMC4498784

[48] Sato M Nagata K Kuroda S Horiuchi S Nakamura T Karima M. Low-intensity pulsed ultrasound activates integrin-mediated mechanotransduction pathway in synovial cells. Ann Biomed Eng. (2014) 42:2156–63. 10.1007/s10439-014-1081-x 25096496

[49] Wei FY Leung KS Li G Qin J Chow SK Huang S. Low intensity pulsed ultrasound enhanced mesenchymal stem cell recruitment through stromal derived factor-1 signaling in fracture healing. PLoS ONE. (2014) 9:e106722. 10.1371/journal.pone.0106722 25181476 PMC4152330

[50] Chan YS Hsu KY Kuo CH Lee SD Chen SC Chen WJ. Using low-intensity pulsed ultrasound to improve muscle healing after laceration injury: an *in vitro* and *in vivo* study. Ultrasound Med Biol. (2010) 36:743–51. 10.1016/j.ultrasmedbio.2010.02.010 20381949

[51] Zhou S Schmelz A Seufferlein T Li Y Zhao J Bachem MG. Molecular mechanisms of low intensity pulsed ultrasound in human skin fibroblasts. J Biol Chem. (2004) 279:54463–9. 10.1074/jbc.M404786200 15485877

[52] Lu H Qin L Cheung W Lee K Wong W Leung K. Low-intensity pulsed ultrasound accelerated bone-tendon junction healing through regulation of vascular endothelial growth factor expression and cartilage formation. Ultrasound Med Biol. (2008) 34:1248–60. 10.1016/j.ultrasmedbio.2008.01.009 18378382

[53] Sexton T Bantignies F Cavalli G. Genomic interactions: chromatin loops and gene meeting points in transcriptional regulation. Semin Cell Dev Biol. (2009) 20:849–55. 10.1016/j.semcdb.2009.06.004 19559093

[54] Dekker J Rippe K Dekker M Kleckner N. Capturing chromosome conformation. Science. (2002) 295:1306–11. 10.1126/science.1067799 11847345

[55] Splinter E de Wit E Nora EP Klous P van de Werken HJ Zhu Y. The inactive X chromosome adopts a unique three-dimensional conformation that is dependent on Xist RNA. Genes Dev. (2011) 25:1371–83. 10.1101/gad.633311 21690198 PMC3134081

[56] Stadhouders R Kolovos P Brouwer R Zuin J van den Heuvel A Kockx C. Multiplexed chromosome conformation capture sequencing for rapid genome-scale high-resolution detection of long-range chromatin interactions. Nat Protoc. (2013) 8:509–24. 10.1038/nprot.2013.018 23411633

[57] Dostie J Richmond TA Arnaout RA Selzer RR Lee WL Honan TA. Chromosome Conformation Capture Carbon Copy (5C): a massively parallel solution for mapping interactions between genomic elements. Genome Res. (2006) 16:1299–309. 10.1101/gr.5571506 16954542 PMC1581439

[58] Acuña LIG Kornblihtt AR. Long range chromatin organization. Transcription. (2014) 5:e28726. 10.4161/trns.28726 25764333 PMC4574877

[59] Park JH Choi Y Song MJ Park K Lee JJ Kim HP. Dynamic long-range chromatin interaction controls expression of IL-21 in CD4+ T cells. J Immunol. (2016) 196:4378–89. 10.4049/jimmunol.1500636 27067007

[60] Deligianni C Spilianakis CG. Long-range genomic interactions epigenetically regulate the expression of a cytokine receptor. EMBO Rep. (2012) 13:819–26. 10.1038/embor.2012.112 22836578 PMC3432804

[61] Montefiori LE Sobreira DR Sakabe NJ Aneas I Joslin AC Hansen GT. A promoter interaction map for cardiovascular disease genetics. Elife. (2018) 7:e35788. 10.7554/eLife.35788 29988018 PMC6053306

[62] Lu Y Sun Y Drummer Ct Nanayakkara GK Shao Y Saaoud F. Increased acetylation of H3K14 in the genomic regions that encode trained immunity enzymes in lysophosphatidylcholine-activated human aortic endothelial cells - novel qualification markers for chronic disease risk factors and conditional DAMPs. Redox Biol. (2019) 24:101221. 10.1016/j.redox.2019.101221 31153039 PMC6543097

[63] Li YF Nanayakkara G Sun Y Li X Wang L Cueto R. Analyses of caspase-1-regulated transcriptomes in various tissues lead to identification of novel IL-1beta-, IL-18- and sirtuin-1-independent pathways. J Hematol Oncol. (2017) 10:40. 10.1186/s13045-017-0406-2 28153032 PMC5290602

[64] Eisenberg E Levanon EY. Human housekeeping genes, revisited. Trends Genet. (2013) 29:569–74. 10.1016/j.tig.2013.05.010 23810203

[65] Wang L Fu H Nanayakkara G Li Y Shao Y Johnson C. Novel extracellular and nuclear caspase-1 and inflammasomes propagate inflammation and regulate gene expression: a comprehensive database mining study. J Hematol Oncol. (2016) 9:122. 10.1186/s13045-016-0351-5 27842563 PMC5109738

[66] Teng L He B Wang J Tan K. 4DGenome: a comprehensive database of chromatin interactions. Bioinformatics. (2015) 31:2560–4. 10.1093/bioinformatics/btv158 25788621 PMC4514924

[67] Li A Sun Y Drummer Ct Lu Y Yu D Zhou Y. Increasing upstream chromatin long-range interactions may favor induction of circular RNAs in LysoPC-activated human aortic endothelial cells. Front Physiol. (2019) 10:433. 10.3389/fphys.2019.00433 31057422 PMC6482593

[68] Lejbkowicz F Salzberg S. Distinct sensitivity of normal and malignant cells to ultrasound *in vitro*. Environ Health Perspect. (1997) 105(Suppl. 6):1575–8. 10.1289/ehp.97105s61575 9467085 PMC1469937

[69] Galluzzi L Vitale I Aaronson SA Abrams JM Adam D Agostinis P. Molecular mechanisms of cell death: recommendations of the Nomenclature Committee on Cell Death 2018. Cell Death Differ. (2018) 25:486–541. 10.1038/s41418-018-0102-y 29362479 PMC5864239

[70] Witt A Vucic D. Diverse ubiquitin linkages regulate RIP kinases-mediated inflammatory and cell death signaling. Cell Death Differ. (2017) 24:1160–71. 10.1038/cdd.2017.33 28475174 PMC5520166

[71] Yin Y Pastrana JL Li X Huang X Mallilankaraman K Choi ET. Inflammasomes: sensors of metabolic stresses for vascular inflammation. Front Biosci. (2013) 18:638–49. 10.2741/4127 23276949 PMC3590813

[72] Songane M Khair M Saleh M. An updated view on the functions of caspases in inflammation and immunity. Semin Cell Dev Biol. (2018) 82:137–49. 10.1016/j.semcdb.2018.01.001 29366812

[73] Sabapathy K Lane DP. Therapeutic targeting of p53: all mutants are equal, but some mutants are more equal than others. Nat Rev Clin Oncol. (2018) 15:13–30. 10.1038/nrclinonc.2017.151 28948977

[74] Shiloh Y Ziv Y The ATM protein kinase: regulating the cellular response to genotoxic stress, and more. Nat Rev Mol Cell Biol. (2013) 14:197–210. 10.1038/nrm3546 23847781

[75] Oliveira AG Araujo TG Carvalho BM Rocha GZ Santos A Saad MJA. The role of Hepatocyte Growth Factor (HGF) in insulin resistance and diabetes. Front Endocrinol. (2018) 9:503. 10.3389/fendo.2018.00503 30214428 PMC6125308

[76] Baker KG Robertson VJ Duck FA. A review of therapeutic ultrasound: biophysical effects. Phys Ther. (2001) 81:1351–8. 10.1093/ptj/81.7.1351 11444998

[77] Jain A Tiwari A Verma A Jain SK. Ultrasound-based triggered drug delivery to tumors. Drug Deliv Transl Res. (2018) 8:150–64. 10.1007/s13346-017-0448-6 29204925

[78] Khanna A Nelmes RTC Gougoulias N Maffulli N Gray J. The effects of LIPUS on soft-tissue healing: a review of literature. Br Med Bull. (2009) 89:169–82. 10.1093/bmb/ldn040 19011263

[79] Xin ZC Lin GT Lei HG Lue TF Guo YL. Clinical applications of low-intensity pulsed ultrasound and its potential role in urology. Transl Androl Urol. (2016) 5:255–66. 10.21037/tau.2016.02.04 27141455 PMC4837316

[80] Rigby JH Taggart RM Stratton KL Lewis GK Draper DO. Intramuscular heating characteristics of multihour low-intensity therapeutic ultrasound. J Athletic Train. (2015) 50:1158–64. 10.4085/1062-6050-50.11.03 26509683 PMC4732395

[81] Siti HN Kamisah Y Kamsiah J. The role of oxidative stress, antioxidants and vascular inflammation in cardiovascular disease (a review). Vascul Pharmacol. (2015) 71:40–56. 10.1016/j.vph.2015.03.005 25869516

[82] Sosa V Moline T Somoza R Paciucci R Kondoh H ME LL. Oxidative stress and cancer: an overview. Ageing Res Rev. (2013) 12:376–90. 10.1016/j.arr.2012.10.004 23123177

[83] Akman T Akarsu M Akpinar H Resmi H Taylan E. Erythrocyte deformability and oxidative stress in inflammatory bowel disease. Dig Dis Sci. (2012) 57:458–64. 10.1007/s10620-011-1882-9 21901259

[84] Radi E Formichi P Battisti C Federico A. Apoptosis and oxidative stress in neurodegenerative diseases. J Alzheimers Dis. (2014) 42(Suppl. 3):S125–52. 10.3233/JAD-132738 25056458

[85] Li J Zhang Q Ren C Wu X Zhang Y Bai X. Low-intensity pulsed ultrasound prevents the oxidative stress induced endothelial-mesenchymal transition in human aortic endothelial cells. Cell Physiol Biochem. (2018) 45:1350–65. 10.1159/000487561 29462805

[86] Kim NK Kim CY Choi MJ Park SR Choi BH. Effects of low-intensity ultrasound on oxidative damage in retinal pigment epithelial cells *in vitro*. Ultrasound Med Biol. (2015) 41:1363–71. 10.1016/j.ultrasmedbio.2014.12.665 25722027

[87] Li JH Chen ZQ Huang Z Zhan Q Ren FB Liu JY. *In vitro* study of low intensity ultrasound combined with different doses of PDT: effects on C6 glioma cells. Oncol Lett. (2013) 5:702–6. 10.3892/ol.2012.1060 23420417 PMC3573141

[88] Shimamura Y Tamatani D Kuniyasu S Mizuki Y Suzuki T Katsura H. 5-Aminolevulinic acid enhances ultrasound-mediated antitumor activity via mitochondrial oxidative damage in breast cancer. Anticancer Res. (2016) 36:3607–12. 27354630

[89] Ryter SW Kim HP Hoetzel A Park JW Nakahira K Wang X. Mechanisms of cell death in oxidative stress. Antioxid Redox Signal. (2007) 9:49–89. 10.1089/ars.2007.9.49 17115887

[90] Shao Y Chernaya V Johnson C Yang WY Cueto R Sha X Metabolic diseases downregulate the majority of histone modification enzymes, making a few upregulated enzymes novel therapeutic targets–“sand out and gold stays”. J Cardiovasc Transl Res. (2016) 9:49–66. 10.1007/s12265-015-9664-y 26746407 PMC4767600

[91] Li X Shao Y Sha X Fang P Kuo YM Andrews AJ. IL-35 (Interleukin-35) suppresses endothelial cell activation by inhibiting mitochondrial reactive oxygen species-mediated site-specific acetylation of H3K14 (Histone 3 Lysine 14). Arterioscler Thromb Vasc Biol. (2018) 38:599–609. 10.1161/ATVBAHA.117.310626 29371247 PMC5823772

[92] Zeng H Nanayakkara GK Shao Y Fu H Sun Y Cueto R. DNA checkpoint and repair factors are nuclear sensors for intracellular organelle stresses-inflammations and cancers can have high genomic risks. Front Physiol. (2018) 9:516. 10.3389/fphys.2018.00516 29867559 PMC5958474

[93] Lan X Witt H Katsumura K Ye Z Wang Q Bresnick EH. Integration of Hi-C and ChIP-seq data reveals distinct types of chromatin linkages. Nucleic Acids Res. (2012) 40:7690–704. 10.1093/nar/gks501 22675074 PMC3439894

[94] Lu H Qin L Lee K Cheung W Chan K Leung K. Identification of genes responsive to low-intensity pulsed ultrasound stimulations. Biochem Biophys Res Commun. (2009) 378:569–73. 10.1016/j.bbrc.2008.11.074 19056340

[95] Hundt W Yuh EL Steinbach S Bednarski MD Guccione S. Comparison of continuous vs. pulsed focused ultrasound in treated muscle tissue as evaluated by magnetic resonance imaging, histological analysis, and microarray analysis. Eur Radiol. (2008) 18:993–1004. 10.1007/s00330-007-0848-y 18205005

[96] Tabuchi Y Ando H Takasaki I Feril LB Jr Zhao QL Ogawa R. Identification of genes responsive to low intensity pulsed ultrasound in a human leukemia cell line Molt-4. Cancer Lett. (2007) 246:149–56. 10.1016/j.canlet.2006.02.011 16678341

[97] Ng B Yang F Huston DP Yan Y Yang Y Xiong Z. Increased noncanonical splicing of autoantigen transcripts provides the structural basis for expression of untolerized epitopes. J Allergy Clin Immunol. (2004) 114:1463–70. 10.1016/j.jaci.2004.09.006 15577853 PMC3902068

[98] Yin Y Yan Y Jiang X Mai J Chen NC Wang H. Inflammasomes are differentially expressed in cardiovascular and other tissues. Int J Immunopathol Pharmacol. (2009) 22:311–22. 10.1177/039463200902200208 19505385 PMC2847797

[99] Li X Mai J Virtue A Yin Y Gong R Sha X. IL-35 is a novel responsive anti-inflammatory cytokine–a new system of categorizing anti-inflammatory cytokines. PLoS ONE. (2012) 7:e33628. 10.1371/journal.pone.0033628 22438968 PMC3306427

[100] Nejad SM Hosseini H Akiyama H Tachibana K. Reparable cell sonoporation in suspension: theranostic potential of microbubble. Theranostics. (2016) 6:446–55. 10.7150/thno.13518 26941839 PMC4775856

[101] Schiffer JT Mayer BT Fong Y Swan DA Wald A. Herpes simplex virus-2 transmission probability estimates based on quantity of viral shedding. J R Soc Interface. (2014) 11:20140160. 10.1098/rsif.2014.0160 24671939 PMC4006256

[102] Juffermans LJ Dijkmans PA Musters RJ Visser CA Kamp O. Transient permeabilization of cell membranes by ultrasound-exposed microbubbles is related to formation of hydrogen peroxide. Am J Physiol Heart Circ Physiol. (2006) 291:H1595–601. 10.1152/ajpheart.01120.2005 16632548

[103] Park J Fan Z Kumon RE El-Sayed ME Deng CX. Modulation of intracellular Ca2+ concentration in brain microvascular endothelial cells *in vitro* by acoustic cavitation. Ultrasound Med Biol. (2010) 36:1176–87. 10.1016/j.ultrasmedbio.2010.04.006 20620704 PMC3139909

