# Supplementary material for: Experimental Data-Mining Analyses Reveal New Roles of Low-Intensity Ultrasound in Differentiating Cell Death Regulatome in Cancer and Non-cancer Cells via Potential Modulation of Chromatin Long-Range Interactions
Source: Front Oncol. 2019 Jul 12;9:600. doi: 10.3389/fonc.2019.00600 (PMC6640725; doi:10.3389/fonc.2019.00600)

## Slide 1
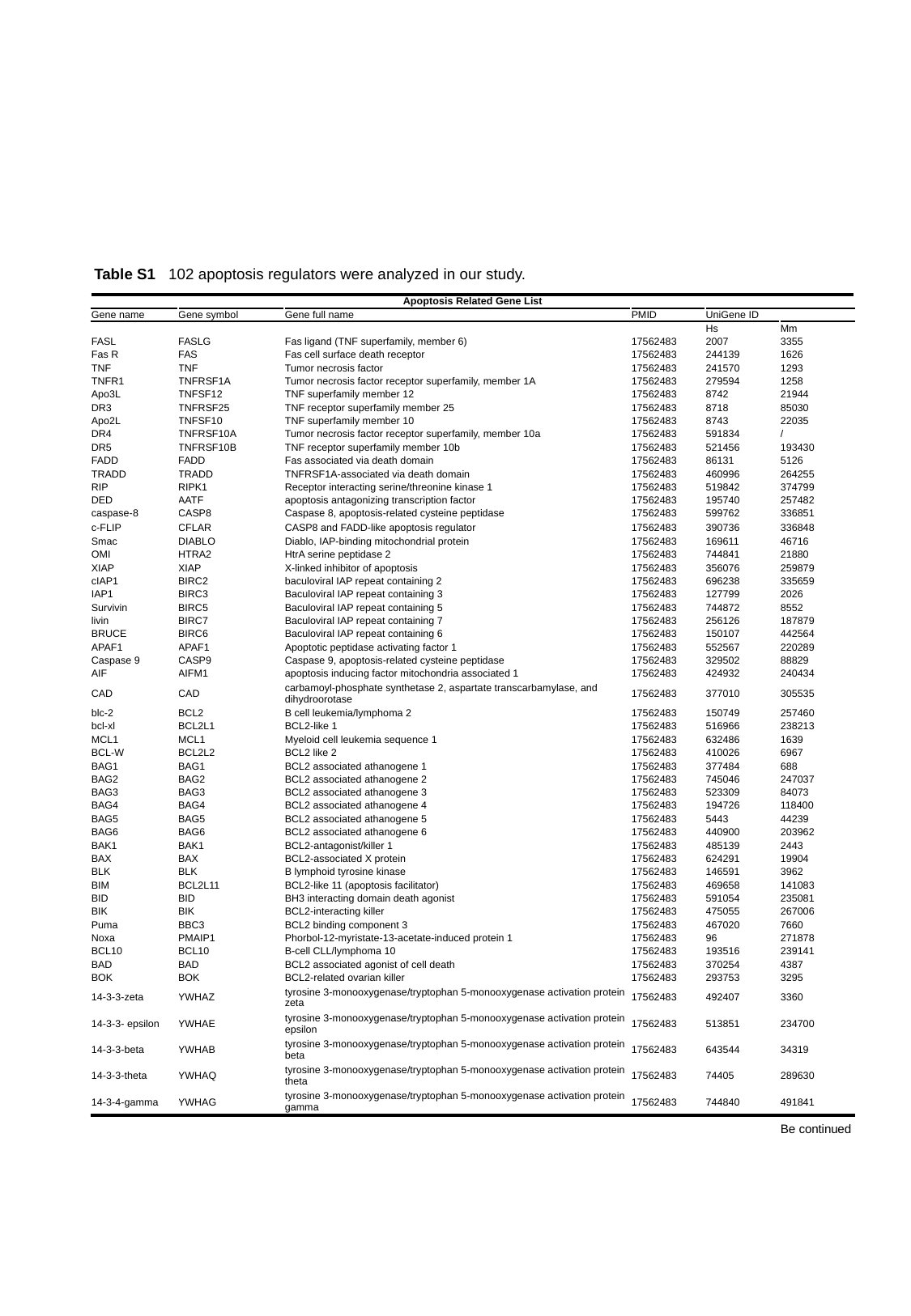

Table S1 102 apoptosis regulators were analyzed in our study.
| Apoptosis Related Gene List | | | | | |
| --- | --- | --- | --- | --- | --- |
| Gene name | Gene symbol | Gene full name | PMID | UniGene ID | |
| | | | | Hs | Mm |
| FASL | FASLG | Fas ligand (TNF superfamily, member 6) | 17562483 | 2007 | 3355 |
| Fas R | FAS | Fas cell surface death receptor | 17562483 | 244139 | 1626 |
| TNF | TNF | Tumor necrosis factor | 17562483 | 241570 | 1293 |
| TNFR1 | TNFRSF1A | Tumor necrosis factor receptor superfamily, member 1A | 17562483 | 279594 | 1258 |
| Apo3L | TNFSF12 | TNF superfamily member 12 | 17562483 | 8742 | 21944 |
| DR3 | TNFRSF25 | TNF receptor superfamily member 25 | 17562483 | 8718 | 85030 |
| Apo2L | TNFSF10 | TNF superfamily member 10 | 17562483 | 8743 | 22035 |
| DR4 | TNFRSF10A | Tumor necrosis factor receptor superfamily, member 10a | 17562483 | 591834 | / |
| DR5 | TNFRSF10B | TNF receptor superfamily member 10b | 17562483 | 521456 | 193430 |
| FADD | FADD | Fas associated via death domain | 17562483 | 86131 | 5126 |
| TRADD | TRADD | TNFRSF1A-associated via death domain | 17562483 | 460996 | 264255 |
| RIP | RIPK1 | Receptor interacting serine/threonine kinase 1 | 17562483 | 519842 | 374799 |
| DED | AATF | apoptosis antagonizing transcription factor | 17562483 | 195740 | 257482 |
| caspase-8 | CASP8 | Caspase 8, apoptosis-related cysteine peptidase | 17562483 | 599762 | 336851 |
| c-FLIP | CFLAR | CASP8 and FADD-like apoptosis regulator | 17562483 | 390736 | 336848 |
| Smac | DIABLO | Diablo, IAP-binding mitochondrial protein | 17562483 | 169611 | 46716 |
| OMI | HTRA2 | HtrA serine peptidase 2 | 17562483 | 744841 | 21880 |
| XIAP | XIAP | X-linked inhibitor of apoptosis | 17562483 | 356076 | 259879 |
| cIAP1 | BIRC2 | baculoviral IAP repeat containing 2 | 17562483 | 696238 | 335659 |
| IAP1 | BIRC3 | Baculoviral IAP repeat containing 3 | 17562483 | 127799 | 2026 |
| Survivin | BIRC5 | Baculoviral IAP repeat containing 5 | 17562483 | 744872 | 8552 |
| livin | BIRC7 | Baculoviral IAP repeat containing 7 | 17562483 | 256126 | 187879 |
| BRUCE | BIRC6 | Baculoviral IAP repeat containing 6 | 17562483 | 150107 | 442564 |
| APAF1 | APAF1 | Apoptotic peptidase activating factor 1 | 17562483 | 552567 | 220289 |
| Caspase 9 | CASP9 | Caspase 9, apoptosis-related cysteine peptidase | 17562483 | 329502 | 88829 |
| AIF | AIFM1 | apoptosis inducing factor mitochondria associated 1 | 17562483 | 424932 | 240434 |
| CAD | CAD | carbamoyl-phosphate synthetase 2, aspartate transcarbamylase, and dihydroorotase | 17562483 | 377010 | 305535 |
| blc-2 | BCL2 | B cell leukemia/lymphoma 2 | 17562483 | 150749 | 257460 |
| bcl-xl | BCL2L1 | BCL2-like 1 | 17562483 | 516966 | 238213 |
| MCL1 | MCL1 | Myeloid cell leukemia sequence 1 | 17562483 | 632486 | 1639 |
| BCL-W | BCL2L2 | BCL2 like 2 | 17562483 | 410026 | 6967 |
| BAG1 | BAG1 | BCL2 associated athanogene 1 | 17562483 | 377484 | 688 |
| BAG2 | BAG2 | BCL2 associated athanogene 2 | 17562483 | 745046 | 247037 |
| BAG3 | BAG3 | BCL2 associated athanogene 3 | 17562483 | 523309 | 84073 |
| BAG4 | BAG4 | BCL2 associated athanogene 4 | 17562483 | 194726 | 118400 |
| BAG5 | BAG5 | BCL2 associated athanogene 5 | 17562483 | 5443 | 44239 |
| BAG6 | BAG6 | BCL2 associated athanogene 6 | 17562483 | 440900 | 203962 |
| BAK1 | BAK1 | BCL2-antagonist/killer 1 | 17562483 | 485139 | 2443 |
| BAX | BAX | BCL2-associated X protein | 17562483 | 624291 | 19904 |
| BLK | BLK | B lymphoid tyrosine kinase | 17562483 | 146591 | 3962 |
| BIM | BCL2L11 | BCL2-like 11 (apoptosis facilitator) | 17562483 | 469658 | 141083 |
| BID | BID | BH3 interacting domain death agonist | 17562483 | 591054 | 235081 |
| BIK | BIK | BCL2-interacting killer | 17562483 | 475055 | 267006 |
| Puma | BBC3 | BCL2 binding component 3 | 17562483 | 467020 | 7660 |
| Noxa | PMAIP1 | Phorbol-12-myristate-13-acetate-induced protein 1 | 17562483 | 96 | 271878 |
| BCL10 | BCL10 | B-cell CLL/lymphoma 10 | 17562483 | 193516 | 239141 |
| BAD | BAD | BCL2 associated agonist of cell death | 17562483 | 370254 | 4387 |
| BOK | BOK | BCL2-related ovarian killer | 17562483 | 293753 | 3295 |
| 14-3-3-zeta | YWHAZ | tyrosine 3-monooxygenase/tryptophan 5-monooxygenase activation protein zeta | 17562483 | 492407 | 3360 |
| 14-3-3- epsilon | YWHAE | tyrosine 3-monooxygenase/tryptophan 5-monooxygenase activation protein epsilon | 17562483 | 513851 | 234700 |
| 14-3-3-beta | YWHAB | tyrosine 3-monooxygenase/tryptophan 5-monooxygenase activation protein beta | 17562483 | 643544 | 34319 |
| 14-3-3-theta | YWHAQ | tyrosine 3-monooxygenase/tryptophan 5-monooxygenase activation protein theta | 17562483 | 74405 | 289630 |
| 14-3-4-gamma | YWHAG | tyrosine 3-monooxygenase/tryptophan 5-monooxygenase activation protein gamma | 17562483 | 744840 | 491841 |
Be continued

## Slide 2
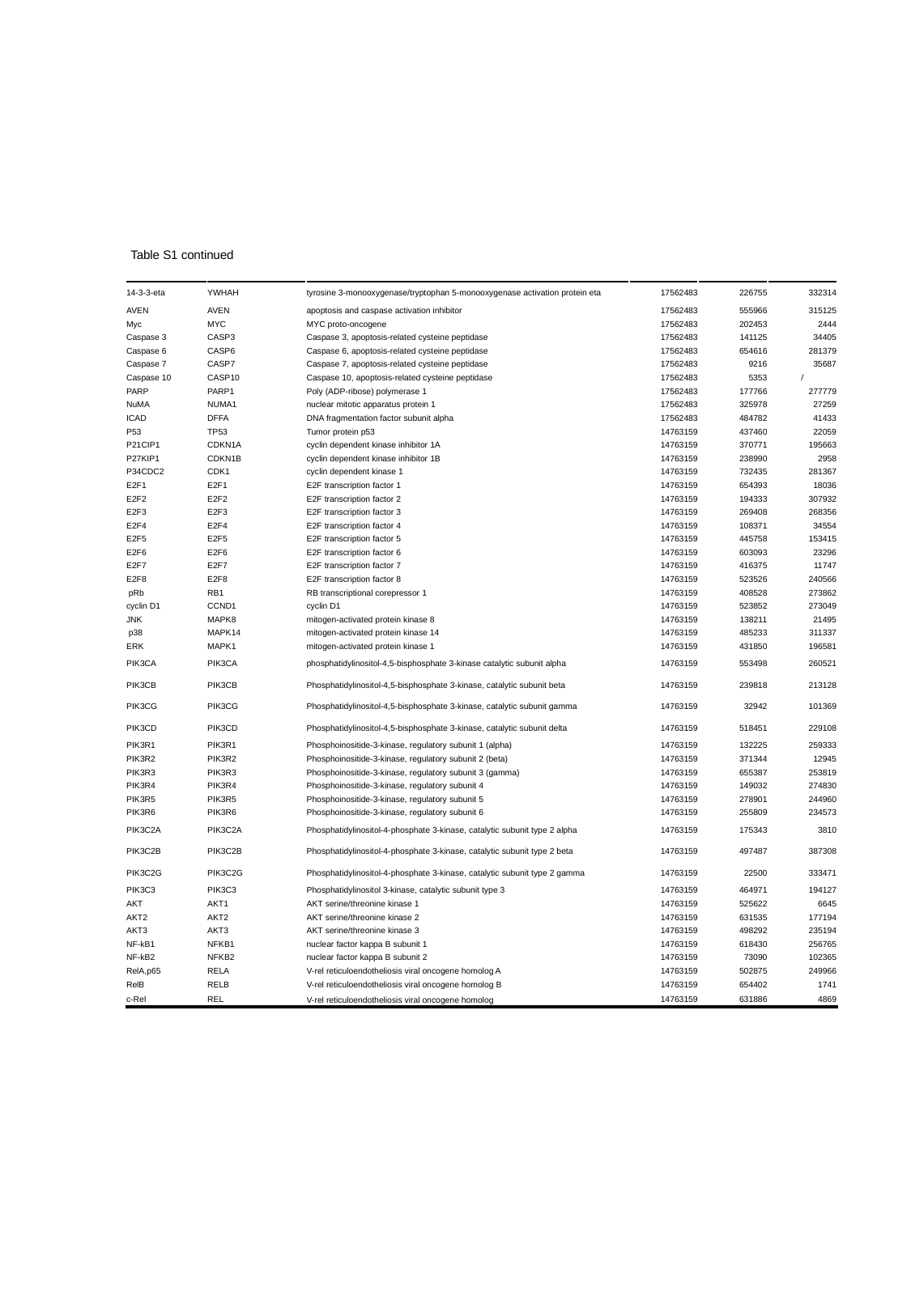

Table S1 continued
| 14-3-3-eta | YWHAH | tyrosine 3-monooxygenase/tryptophan 5-monooxygenase activation protein eta | 17562483 | 226755 | 332314 |
| --- | --- | --- | --- | --- | --- |
| AVEN | AVEN | apoptosis and caspase activation inhibitor | 17562483 | 555966 | 315125 |
| Myc | MYC | MYC proto-oncogene | 17562483 | 202453 | 2444 |
| Caspase 3 | CASP3 | Caspase 3, apoptosis-related cysteine peptidase | 17562483 | 141125 | 34405 |
| Caspase 6 | CASP6 | Caspase 6, apoptosis-related cysteine peptidase | 17562483 | 654616 | 281379 |
| Caspase 7 | CASP7 | Caspase 7, apoptosis-related cysteine peptidase | 17562483 | 9216 | 35687 |
| Caspase 10 | CASP10 | Caspase 10, apoptosis-related cysteine peptidase | 17562483 | 5353 | / |
| PARP | PARP1 | Poly (ADP-ribose) polymerase 1 | 17562483 | 177766 | 277779 |
| NuMA | NUMA1 | nuclear mitotic apparatus protein 1 | 17562483 | 325978 | 27259 |
| ICAD | DFFA | DNA fragmentation factor subunit alpha | 17562483 | 484782 | 41433 |
| P53 | TP53 | Tumor protein p53 | 14763159 | 437460 | 22059 |
| P21CIP1 | CDKN1A | cyclin dependent kinase inhibitor 1A | 14763159 | 370771 | 195663 |
| P27KIP1 | CDKN1B | cyclin dependent kinase inhibitor 1B | 14763159 | 238990 | 2958 |
| P34CDC2 | CDK1 | cyclin dependent kinase 1 | 14763159 | 732435 | 281367 |
| E2F1 | E2F1 | E2F transcription factor 1 | 14763159 | 654393 | 18036 |
| E2F2 | E2F2 | E2F transcription factor 2 | 14763159 | 194333 | 307932 |
| E2F3 | E2F3 | E2F transcription factor 3 | 14763159 | 269408 | 268356 |
| E2F4 | E2F4 | E2F transcription factor 4 | 14763159 | 108371 | 34554 |
| E2F5 | E2F5 | E2F transcription factor 5 | 14763159 | 445758 | 153415 |
| E2F6 | E2F6 | E2F transcription factor 6 | 14763159 | 603093 | 23296 |
| E2F7 | E2F7 | E2F transcription factor 7 | 14763159 | 416375 | 11747 |
| E2F8 | E2F8 | E2F transcription factor 8 | 14763159 | 523526 | 240566 |
| pRb | RB1 | RB transcriptional corepressor 1 | 14763159 | 408528 | 273862 |
| cyclin D1 | CCND1 | cyclin D1 | 14763159 | 523852 | 273049 |
| JNK | MAPK8 | mitogen-activated protein kinase 8 | 14763159 | 138211 | 21495 |
| p38 | MAPK14 | mitogen-activated protein kinase 14 | 14763159 | 485233 | 311337 |
| ERK | MAPK1 | mitogen-activated protein kinase 1 | 14763159 | 431850 | 196581 |
| PIK3CA | PIK3CA | phosphatidylinositol-4,5-bisphosphate 3-kinase catalytic subunit alpha | 14763159 | 553498 | 260521 |
| PIK3CB | PIK3CB | Phosphatidylinositol-4,5-bisphosphate 3-kinase, catalytic subunit beta | 14763159 | 239818 | 213128 |
| PIK3CG | PIK3CG | Phosphatidylinositol-4,5-bisphosphate 3-kinase, catalytic subunit gamma | 14763159 | 32942 | 101369 |
| PIK3CD | PIK3CD | Phosphatidylinositol-4,5-bisphosphate 3-kinase, catalytic subunit delta | 14763159 | 518451 | 229108 |
| PIK3R1 | PIK3R1 | Phosphoinositide-3-kinase, regulatory subunit 1 (alpha) | 14763159 | 132225 | 259333 |
| PIK3R2 | PIK3R2 | Phosphoinositide-3-kinase, regulatory subunit 2 (beta) | 14763159 | 371344 | 12945 |
| PIK3R3 | PIK3R3 | Phosphoinositide-3-kinase, regulatory subunit 3 (gamma) | 14763159 | 655387 | 253819 |
| PIK3R4 | PIK3R4 | Phosphoinositide-3-kinase, regulatory subunit 4 | 14763159 | 149032 | 274830 |
| PIK3R5 | PIK3R5 | Phosphoinositide-3-kinase, regulatory subunit 5 | 14763159 | 278901 | 244960 |
| PIK3R6 | PIK3R6 | Phosphoinositide-3-kinase, regulatory subunit 6 | 14763159 | 255809 | 234573 |
| PIK3C2A | PIK3C2A | Phosphatidylinositol-4-phosphate 3-kinase, catalytic subunit type 2 alpha | 14763159 | 175343 | 3810 |
| PIK3C2B | PIK3C2B | Phosphatidylinositol-4-phosphate 3-kinase, catalytic subunit type 2 beta | 14763159 | 497487 | 387308 |
| PIK3C2G | PIK3C2G | Phosphatidylinositol-4-phosphate 3-kinase, catalytic subunit type 2 gamma | 14763159 | 22500 | 333471 |
| PIK3C3 | PIK3C3 | Phosphatidylinositol 3-kinase, catalytic subunit type 3 | 14763159 | 464971 | 194127 |
| AKT | AKT1 | AKT serine/threonine kinase 1 | 14763159 | 525622 | 6645 |
| AKT2 | AKT2 | AKT serine/threonine kinase 2 | 14763159 | 631535 | 177194 |
| AKT3 | AKT3 | AKT serine/threonine kinase 3 | 14763159 | 498292 | 235194 |
| NF-kB1 | NFKB1 | nuclear factor kappa B subunit 1 | 14763159 | 618430 | 256765 |
| NF-kB2 | NFKB2 | nuclear factor kappa B subunit 2 | 14763159 | 73090 | 102365 |
| RelA,p65 | RELA | V-rel reticuloendotheliosis viral oncogene homolog A | 14763159 | 502875 | 249966 |
| RelB | RELB | V-rel reticuloendotheliosis viral oncogene homolog B | 14763159 | 654402 | 1741 |
| c-Rel | REL | V-rel reticuloendotheliosis viral oncogene homolog | 14763159 | 631886 | 4869 |

## Slide 3
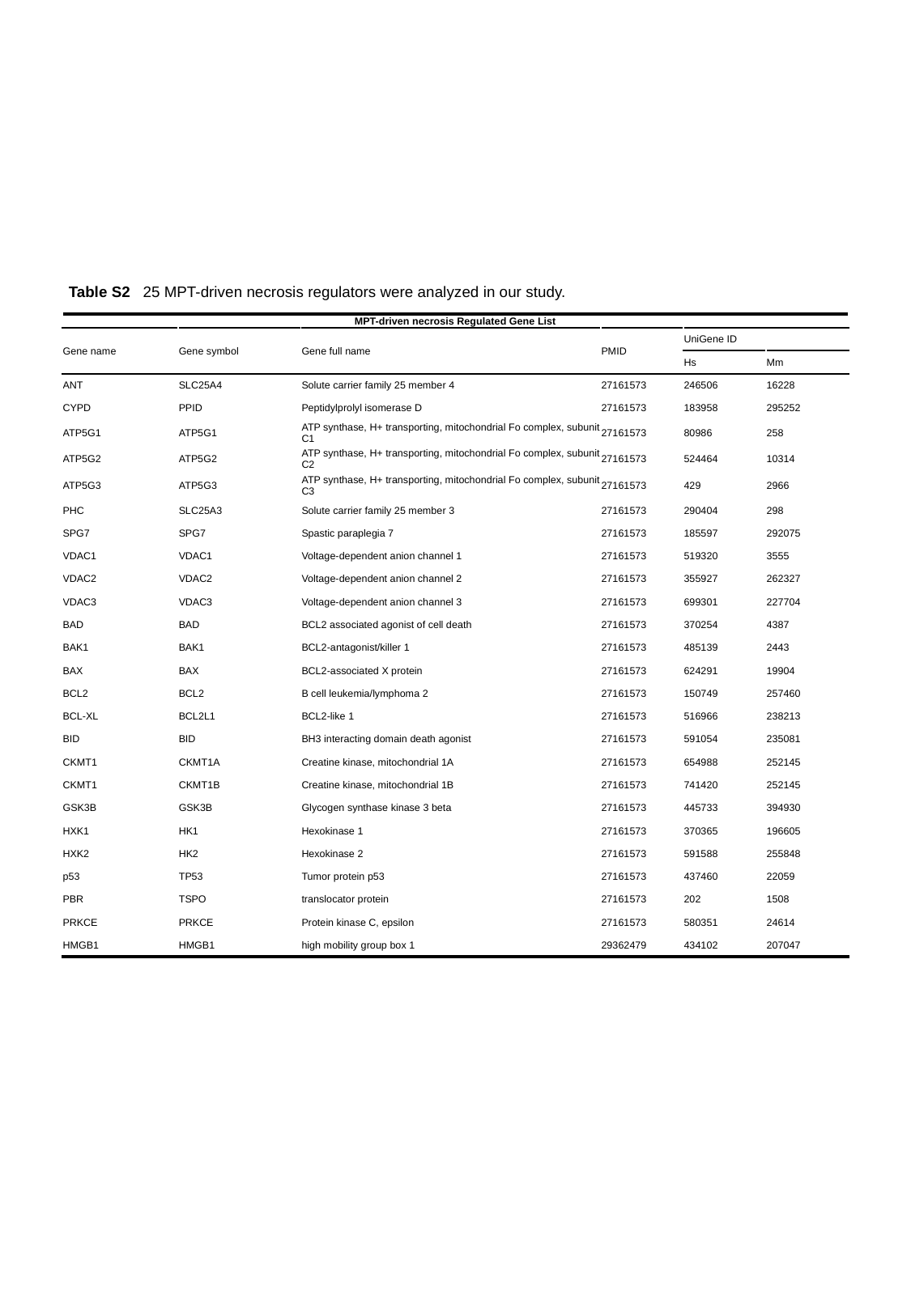

Table S2 25 MPT-driven necrosis regulators were analyzed in our study.
| MPT-driven necrosis Regulated Gene List | | | | | |
| --- | --- | --- | --- | --- | --- |
| Gene name | Gene symbol | Gene full name | PMID | UniGene ID | |
| | | | | Hs | Mm |
| ANT | SLC25A4 | Solute carrier family 25 member 4 | 27161573 | 246506 | 16228 |
| CYPD | PPID | Peptidylprolyl isomerase D | 27161573 | 183958 | 295252 |
| ATP5G1 | ATP5G1 | ATP synthase, H+ transporting, mitochondrial Fo complex, subunit C1 | 27161573 | 80986 | 258 |
| ATP5G2 | ATP5G2 | ATP synthase, H+ transporting, mitochondrial Fo complex, subunit C2 | 27161573 | 524464 | 10314 |
| ATP5G3 | ATP5G3 | ATP synthase, H+ transporting, mitochondrial Fo complex, subunit C3 | 27161573 | 429 | 2966 |
| PHC | SLC25A3 | Solute carrier family 25 member 3 | 27161573 | 290404 | 298 |
| SPG7 | SPG7 | Spastic paraplegia 7 | 27161573 | 185597 | 292075 |
| VDAC1 | VDAC1 | Voltage-dependent anion channel 1 | 27161573 | 519320 | 3555 |
| VDAC2 | VDAC2 | Voltage-dependent anion channel 2 | 27161573 | 355927 | 262327 |
| VDAC3 | VDAC3 | Voltage-dependent anion channel 3 | 27161573 | 699301 | 227704 |
| BAD | BAD | BCL2 associated agonist of cell death | 27161573 | 370254 | 4387 |
| BAK1 | BAK1 | BCL2-antagonist/killer 1 | 27161573 | 485139 | 2443 |
| BAX | BAX | BCL2-associated X protein | 27161573 | 624291 | 19904 |
| BCL2 | BCL2 | B cell leukemia/lymphoma 2 | 27161573 | 150749 | 257460 |
| BCL-XL | BCL2L1 | BCL2-like 1 | 27161573 | 516966 | 238213 |
| BID | BID | BH3 interacting domain death agonist | 27161573 | 591054 | 235081 |
| CKMT1 | CKMT1A | Creatine kinase, mitochondrial 1A | 27161573 | 654988 | 252145 |
| CKMT1 | CKMT1B | Creatine kinase, mitochondrial 1B | 27161573 | 741420 | 252145 |
| GSK3B | GSK3B | Glycogen synthase kinase 3 beta | 27161573 | 445733 | 394930 |
| HXK1 | HK1 | Hexokinase 1 | 27161573 | 370365 | 196605 |
| HXK2 | HK2 | Hexokinase 2 | 27161573 | 591588 | 255848 |
| p53 | TP53 | Tumor protein p53 | 27161573 | 437460 | 22059 |
| PBR | TSPO | translocator protein | 27161573 | 202 | 1508 |
| PRKCE | PRKCE | Protein kinase C, epsilon | 27161573 | 580351 | 24614 |
| HMGB1 | HMGB1 | high mobility group box 1 | 29362479 | 434102 | 207047 |

## Slide 4
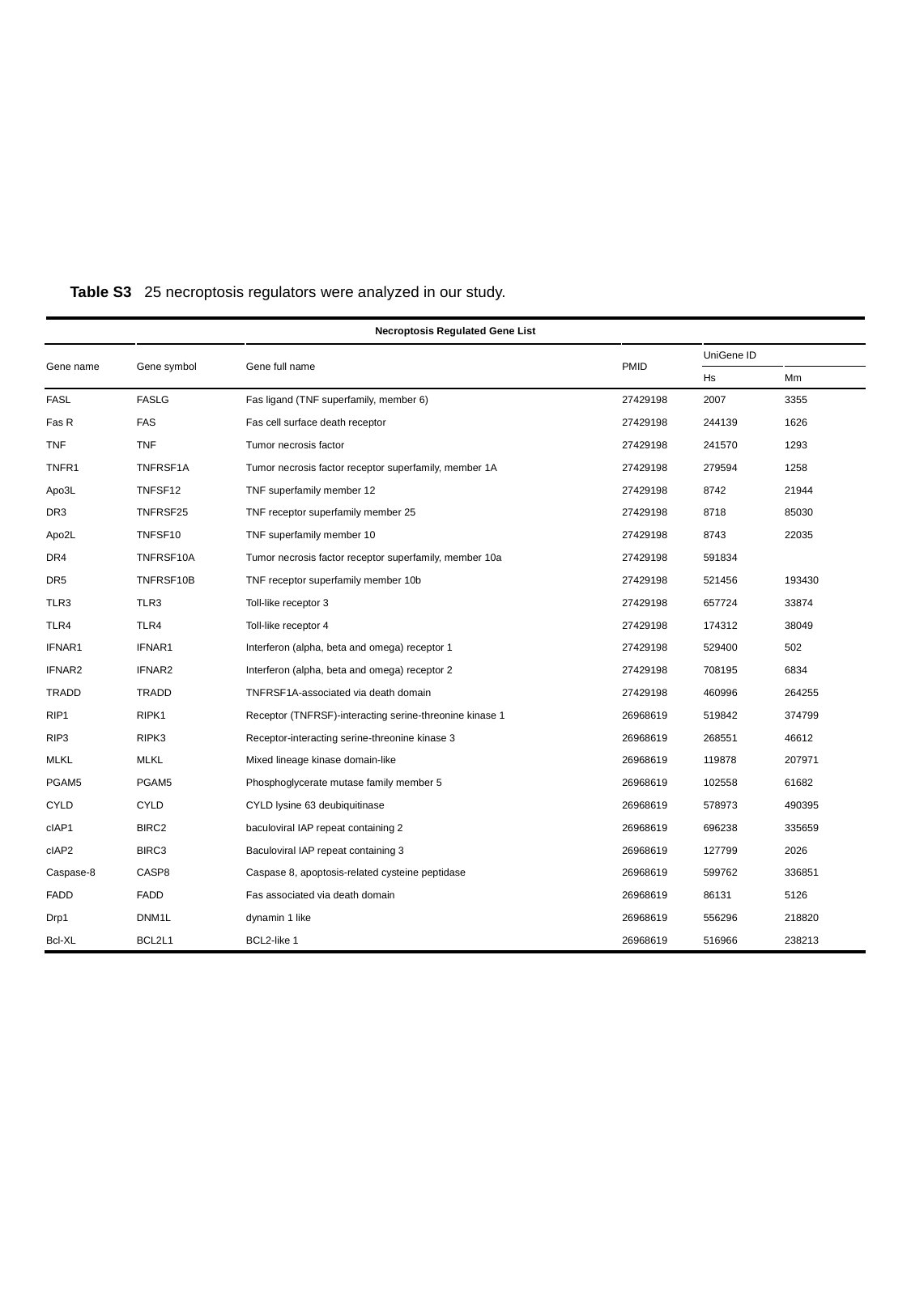

Table S3 25 necroptosis regulators were analyzed in our study.
| Necroptosis Regulated Gene List | | | | | |
| --- | --- | --- | --- | --- | --- |
| Gene name | Gene symbol | Gene full name | PMID | UniGene ID | |
| | | | | Hs | Mm |
| FASL | FASLG | Fas ligand (TNF superfamily, member 6) | 27429198 | 2007 | 3355 |
| Fas R | FAS | Fas cell surface death receptor | 27429198 | 244139 | 1626 |
| TNF | TNF | Tumor necrosis factor | 27429198 | 241570 | 1293 |
| TNFR1 | TNFRSF1A | Tumor necrosis factor receptor superfamily, member 1A | 27429198 | 279594 | 1258 |
| Apo3L | TNFSF12 | TNF superfamily member 12 | 27429198 | 8742 | 21944 |
| DR3 | TNFRSF25 | TNF receptor superfamily member 25 | 27429198 | 8718 | 85030 |
| Apo2L | TNFSF10 | TNF superfamily member 10 | 27429198 | 8743 | 22035 |
| DR4 | TNFRSF10A | Tumor necrosis factor receptor superfamily, member 10a | 27429198 | 591834 | |
| DR5 | TNFRSF10B | TNF receptor superfamily member 10b | 27429198 | 521456 | 193430 |
| TLR3 | TLR3 | Toll-like receptor 3 | 27429198 | 657724 | 33874 |
| TLR4 | TLR4 | Toll-like receptor 4 | 27429198 | 174312 | 38049 |
| IFNAR1 | IFNAR1 | Interferon (alpha, beta and omega) receptor 1 | 27429198 | 529400 | 502 |
| IFNAR2 | IFNAR2 | Interferon (alpha, beta and omega) receptor 2 | 27429198 | 708195 | 6834 |
| TRADD | TRADD | TNFRSF1A-associated via death domain | 27429198 | 460996 | 264255 |
| RIP1 | RIPK1 | Receptor (TNFRSF)-interacting serine-threonine kinase 1 | 26968619 | 519842 | 374799 |
| RIP3 | RIPK3 | Receptor-interacting serine-threonine kinase 3 | 26968619 | 268551 | 46612 |
| MLKL | MLKL | Mixed lineage kinase domain-like | 26968619 | 119878 | 207971 |
| PGAM5 | PGAM5 | Phosphoglycerate mutase family member 5 | 26968619 | 102558 | 61682 |
| CYLD | CYLD | CYLD lysine 63 deubiquitinase | 26968619 | 578973 | 490395 |
| cIAP1 | BIRC2 | baculoviral IAP repeat containing 2 | 26968619 | 696238 | 335659 |
| cIAP2 | BIRC3 | Baculoviral IAP repeat containing 3 | 26968619 | 127799 | 2026 |
| Caspase-8 | CASP8 | Caspase 8, apoptosis-related cysteine peptidase | 26968619 | 599762 | 336851 |
| FADD | FADD | Fas associated via death domain | 26968619 | 86131 | 5126 |
| Drp1 | DNM1L | dynamin 1 like | 26968619 | 556296 | 218820 |
| Bcl-XL | BCL2L1 | BCL2-like 1 | 26968619 | 516966 | 238213 |

## Slide 5
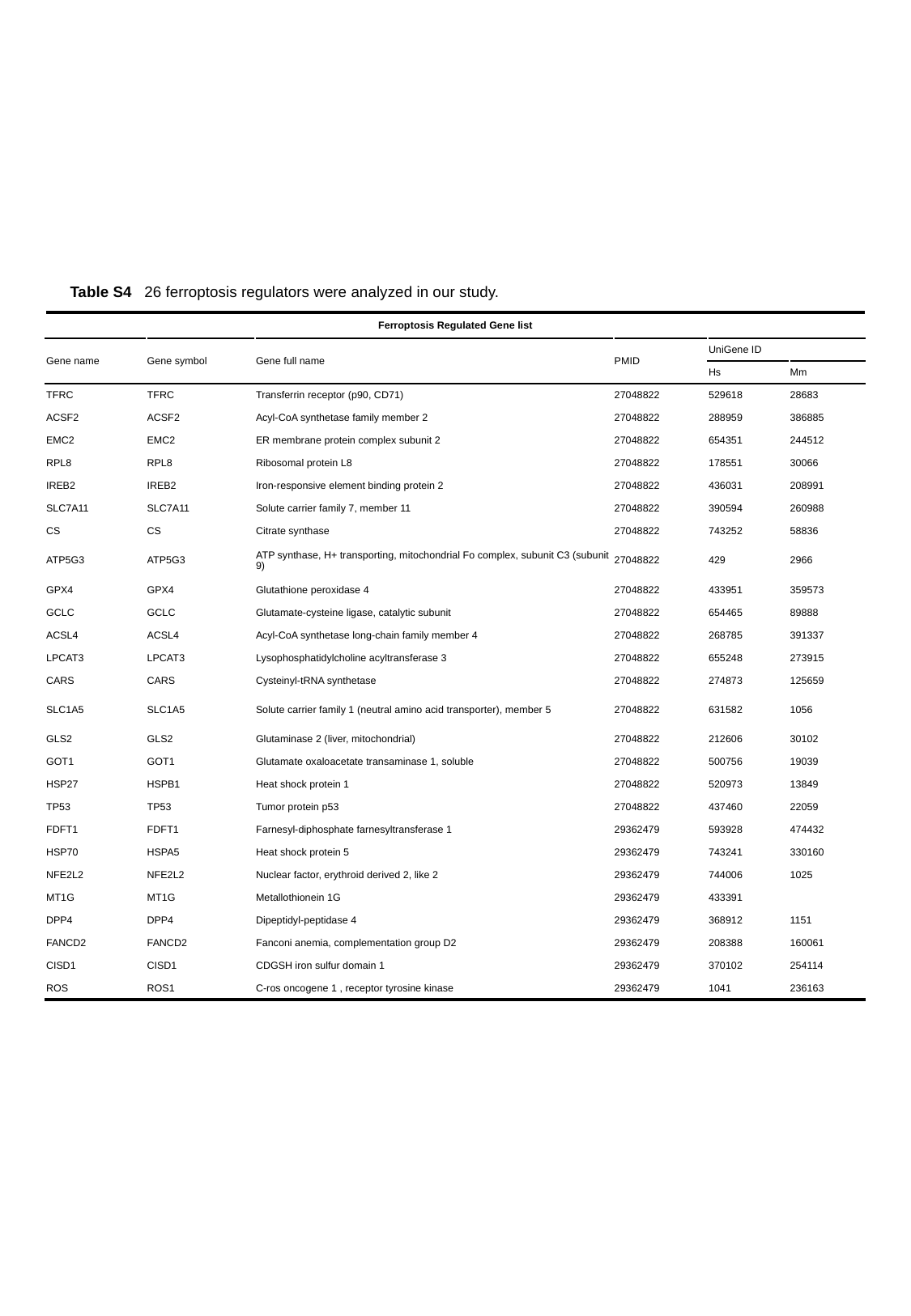

Table S4 26 ferroptosis regulators were analyzed in our study.
| Ferroptosis Regulated Gene list | | | | | |
| --- | --- | --- | --- | --- | --- |
| Gene name | Gene symbol | Gene full name | PMID | UniGene ID | |
| | | | | Hs | Mm |
| TFRC | TFRC | Transferrin receptor (p90, CD71) | 27048822 | 529618 | 28683 |
| ACSF2 | ACSF2 | Acyl-CoA synthetase family member 2 | 27048822 | 288959 | 386885 |
| EMC2 | EMC2 | ER membrane protein complex subunit 2 | 27048822 | 654351 | 244512 |
| RPL8 | RPL8 | Ribosomal protein L8 | 27048822 | 178551 | 30066 |
| IREB2 | IREB2 | Iron-responsive element binding protein 2 | 27048822 | 436031 | 208991 |
| SLC7A11 | SLC7A11 | Solute carrier family 7, member 11 | 27048822 | 390594 | 260988 |
| CS | CS | Citrate synthase | 27048822 | 743252 | 58836 |
| ATP5G3 | ATP5G3 | ATP synthase, H+ transporting, mitochondrial Fo complex, subunit C3 (subunit 9) | 27048822 | 429 | 2966 |
| GPX4 | GPX4 | Glutathione peroxidase 4 | 27048822 | 433951 | 359573 |
| GCLC | GCLC | Glutamate-cysteine ligase, catalytic subunit | 27048822 | 654465 | 89888 |
| ACSL4 | ACSL4 | Acyl-CoA synthetase long-chain family member 4 | 27048822 | 268785 | 391337 |
| LPCAT3 | LPCAT3 | Lysophosphatidylcholine acyltransferase 3 | 27048822 | 655248 | 273915 |
| CARS | CARS | Cysteinyl-tRNA synthetase | 27048822 | 274873 | 125659 |
| SLC1A5 | SLC1A5 | Solute carrier family 1 (neutral amino acid transporter), member 5 | 27048822 | 631582 | 1056 |
| GLS2 | GLS2 | Glutaminase 2 (liver, mitochondrial) | 27048822 | 212606 | 30102 |
| GOT1 | GOT1 | Glutamate oxaloacetate transaminase 1, soluble | 27048822 | 500756 | 19039 |
| HSP27 | HSPB1 | Heat shock protein 1 | 27048822 | 520973 | 13849 |
| TP53 | TP53 | Tumor protein p53 | 27048822 | 437460 | 22059 |
| FDFT1 | FDFT1 | Farnesyl-diphosphate farnesyltransferase 1 | 29362479 | 593928 | 474432 |
| HSP70 | HSPA5 | Heat shock protein 5 | 29362479 | 743241 | 330160 |
| NFE2L2 | NFE2L2 | Nuclear factor, erythroid derived 2, like 2 | 29362479 | 744006 | 1025 |
| MT1G | MT1G | Metallothionein 1G | 29362479 | 433391 | |
| DPP4 | DPP4 | Dipeptidyl-peptidase 4 | 29362479 | 368912 | 1151 |
| FANCD2 | FANCD2 | Fanconi anemia, complementation group D2 | 29362479 | 208388 | 160061 |
| CISD1 | CISD1 | CDGSH iron sulfur domain 1 | 29362479 | 370102 | 254114 |
| ROS | ROS1 | C-ros oncogene 1 , receptor tyrosine kinase | 29362479 | 1041 | 236163 |

## Slide 6
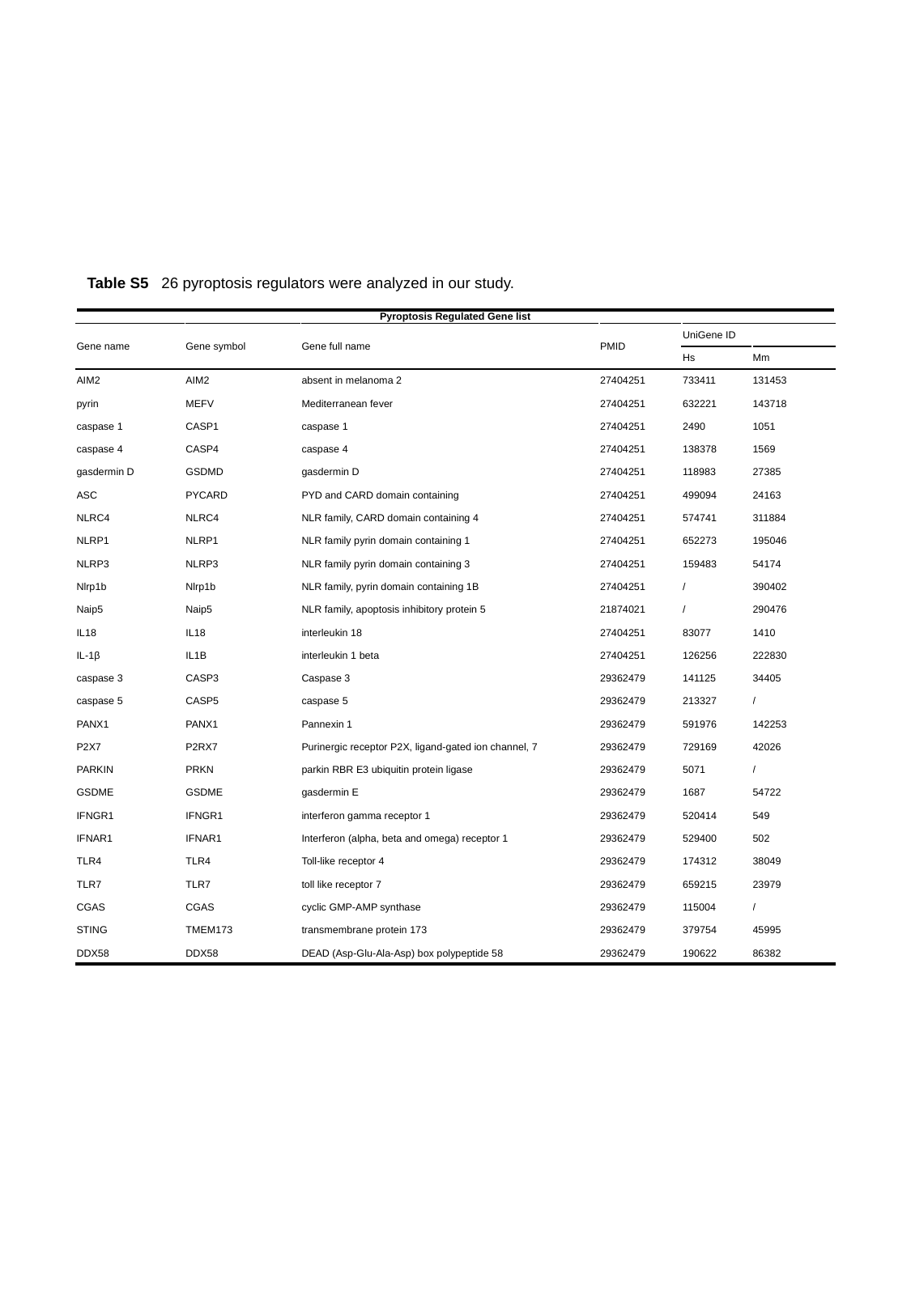

Table S5 26 pyroptosis regulators were analyzed in our study.
| Pyroptosis Regulated Gene list | | | | | |
| --- | --- | --- | --- | --- | --- |
| Gene name | Gene symbol | Gene full name | PMID | UniGene ID | |
| | | | | Hs | Mm |
| AIM2 | AIM2 | absent in melanoma 2 | 27404251 | 733411 | 131453 |
| pyrin | MEFV | Mediterranean fever | 27404251 | 632221 | 143718 |
| caspase 1 | CASP1 | caspase 1 | 27404251 | 2490 | 1051 |
| caspase 4 | CASP4 | caspase 4 | 27404251 | 138378 | 1569 |
| gasdermin D | GSDMD | gasdermin D | 27404251 | 118983 | 27385 |
| ASC | PYCARD | PYD and CARD domain containing | 27404251 | 499094 | 24163 |
| NLRC4 | NLRC4 | NLR family, CARD domain containing 4 | 27404251 | 574741 | 311884 |
| NLRP1 | NLRP1 | NLR family pyrin domain containing 1 | 27404251 | 652273 | 195046 |
| NLRP3 | NLRP3 | NLR family pyrin domain containing 3 | 27404251 | 159483 | 54174 |
| Nlrp1b | Nlrp1b | NLR family, pyrin domain containing 1B | 27404251 | / | 390402 |
| Naip5 | Naip5 | NLR family, apoptosis inhibitory protein 5 | 21874021 | / | 290476 |
| IL18 | IL18 | interleukin 18 | 27404251 | 83077 | 1410 |
| IL-1β | IL1B | interleukin 1 beta | 27404251 | 126256 | 222830 |
| caspase 3 | CASP3 | Caspase 3 | 29362479 | 141125 | 34405 |
| caspase 5 | CASP5 | caspase 5 | 29362479 | 213327 | / |
| PANX1 | PANX1 | Pannexin 1 | 29362479 | 591976 | 142253 |
| P2X7 | P2RX7 | Purinergic receptor P2X, ligand-gated ion channel, 7 | 29362479 | 729169 | 42026 |
| PARKIN | PRKN | parkin RBR E3 ubiquitin protein ligase | 29362479 | 5071 | / |
| GSDME | GSDME | gasdermin E | 29362479 | 1687 | 54722 |
| IFNGR1 | IFNGR1 | interferon gamma receptor 1 | 29362479 | 520414 | 549 |
| IFNAR1 | IFNAR1 | Interferon (alpha, beta and omega) receptor 1 | 29362479 | 529400 | 502 |
| TLR4 | TLR4 | Toll-like receptor 4 | 29362479 | 174312 | 38049 |
| TLR7 | TLR7 | toll like receptor 7 | 29362479 | 659215 | 23979 |
| CGAS | CGAS | cyclic GMP-AMP synthase | 29362479 | 115004 | / |
| STING | TMEM173 | transmembrane protein 173 | 29362479 | 379754 | 45995 |
| DDX58 | DDX58 | DEAD (Asp-Glu-Ala-Asp) box polypeptide 58 | 29362479 | 190622 | 86382 |

## Slide 7
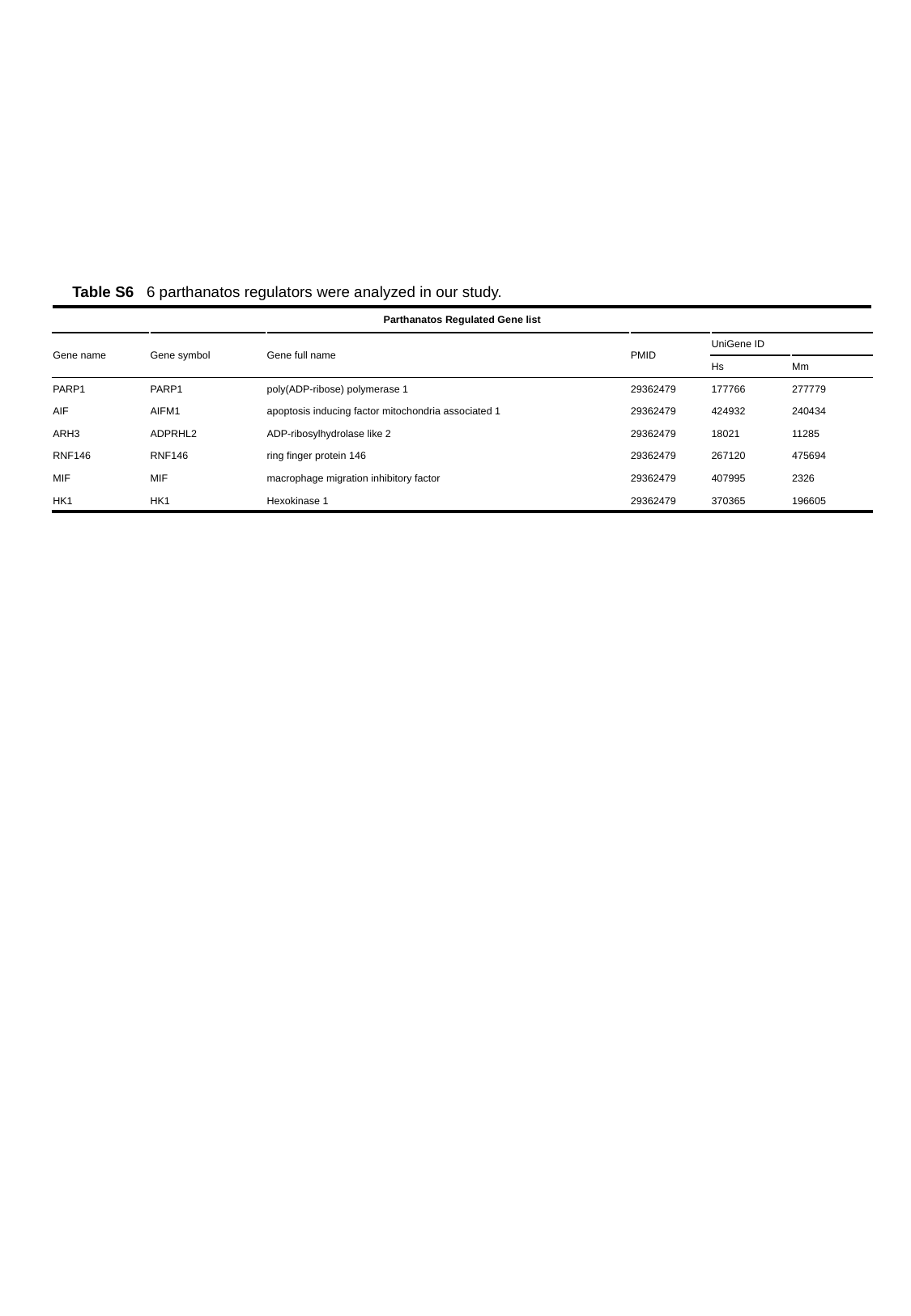

Table S6 6 parthanatos regulators were analyzed in our study.
| Parthanatos Regulated Gene list | | | | | |
| --- | --- | --- | --- | --- | --- |
| Gene name | Gene symbol | Gene full name | PMID | UniGene ID | |
| | | | | Hs | Mm |
| PARP1 | PARP1 | poly(ADP-ribose) polymerase 1 | 29362479 | 177766 | 277779 |
| AIF | AIFM1 | apoptosis inducing factor mitochondria associated 1 | 29362479 | 424932 | 240434 |
| ARH3 | ADPRHL2 | ADP-ribosylhydrolase like 2 | 29362479 | 18021 | 11285 |
| RNF146 | RNF146 | ring finger protein 146 | 29362479 | 267120 | 475694 |
| MIF | MIF | macrophage migration inhibitory factor | 29362479 | 407995 | 2326 |
| HK1 | HK1 | Hexokinase 1 | 29362479 | 370365 | 196605 |

## Slide 8
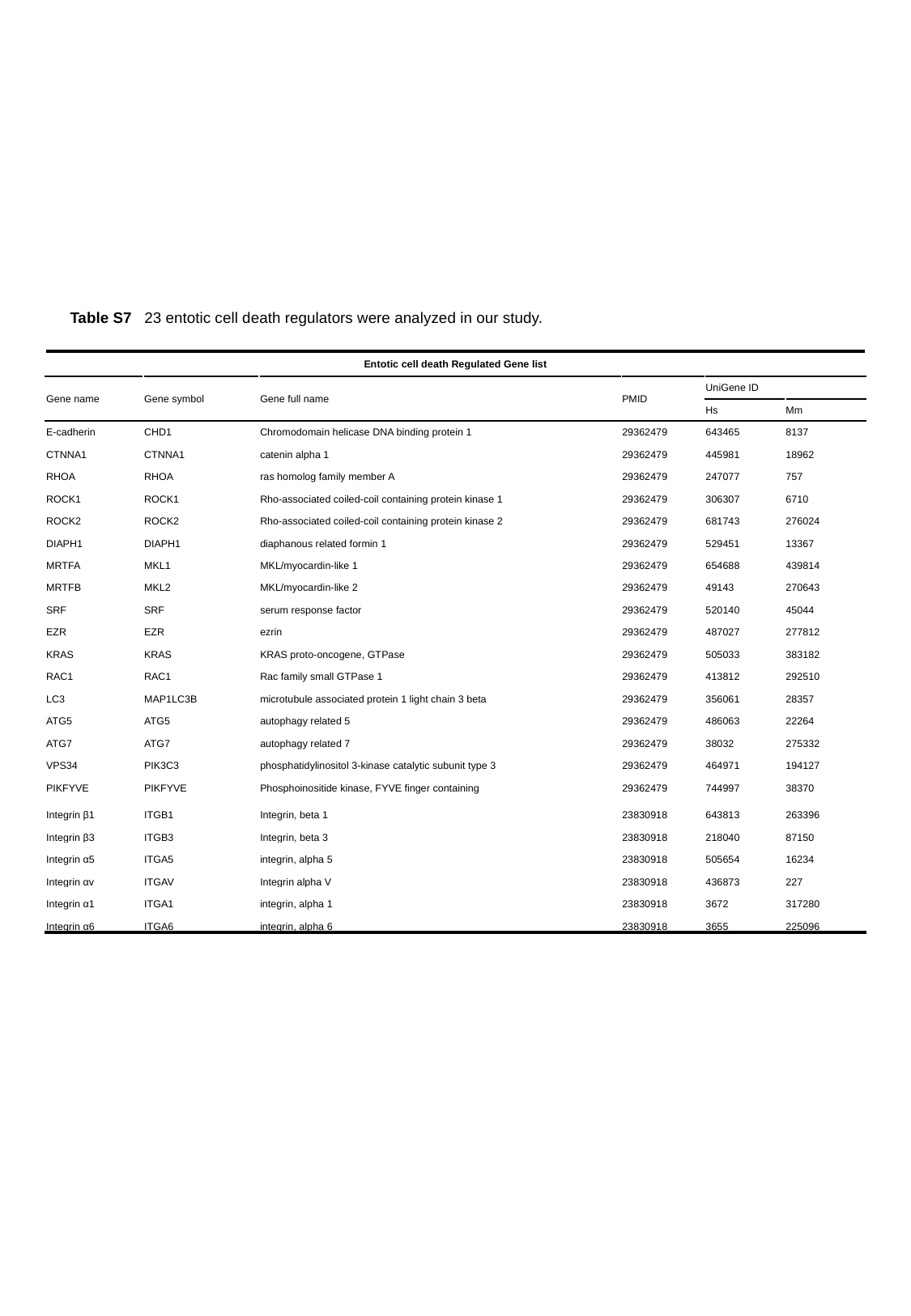

Table S7 23 entotic cell death regulators were analyzed in our study.
| Entotic cell death Regulated Gene list | | | | | |
| --- | --- | --- | --- | --- | --- |
| Gene name | Gene symbol | Gene full name | PMID | UniGene ID | |
| | | | | Hs | Mm |
| E-cadherin | CHD1 | Chromodomain helicase DNA binding protein 1 | 29362479 | 643465 | 8137 |
| CTNNA1 | CTNNA1 | catenin alpha 1 | 29362479 | 445981 | 18962 |
| RHOA | RHOA | ras homolog family member A | 29362479 | 247077 | 757 |
| ROCK1 | ROCK1 | Rho-associated coiled-coil containing protein kinase 1 | 29362479 | 306307 | 6710 |
| ROCK2 | ROCK2 | Rho-associated coiled-coil containing protein kinase 2 | 29362479 | 681743 | 276024 |
| DIAPH1 | DIAPH1 | diaphanous related formin 1 | 29362479 | 529451 | 13367 |
| MRTFA | MKL1 | MKL/myocardin-like 1 | 29362479 | 654688 | 439814 |
| MRTFB | MKL2 | MKL/myocardin-like 2 | 29362479 | 49143 | 270643 |
| SRF | SRF | serum response factor | 29362479 | 520140 | 45044 |
| EZR | EZR | ezrin | 29362479 | 487027 | 277812 |
| KRAS | KRAS | KRAS proto-oncogene, GTPase | 29362479 | 505033 | 383182 |
| RAC1 | RAC1 | Rac family small GTPase 1 | 29362479 | 413812 | 292510 |
| LC3 | MAP1LC3B | microtubule associated protein 1 light chain 3 beta | 29362479 | 356061 | 28357 |
| ATG5 | ATG5 | autophagy related 5 | 29362479 | 486063 | 22264 |
| ATG7 | ATG7 | autophagy related 7 | 29362479 | 38032 | 275332 |
| VPS34 | PIK3C3 | phosphatidylinositol 3-kinase catalytic subunit type 3 | 29362479 | 464971 | 194127 |
| PIKFYVE | PIKFYVE | Phosphoinositide kinase, FYVE finger containing | 29362479 | 744997 | 38370 |
| Integrin β1 | ITGB1 | Integrin, beta 1 | 23830918 | 643813 | 263396 |
| Integrin β3 | ITGB3 | Integrin, beta 3 | 23830918 | 218040 | 87150 |
| Integrin α5 | ITGA5 | integrin, alpha 5 | 23830918 | 505654 | 16234 |
| Integrin αv | ITGAV | Integrin alpha V | 23830918 | 436873 | 227 |
| Integrin α1 | ITGA1 | integrin, alpha 1 | 23830918 | 3672 | 317280 |
| Integrin α6 | ITGA6 | integrin, alpha 6 | 23830918 | 3655 | 225096 |

## Slide 9
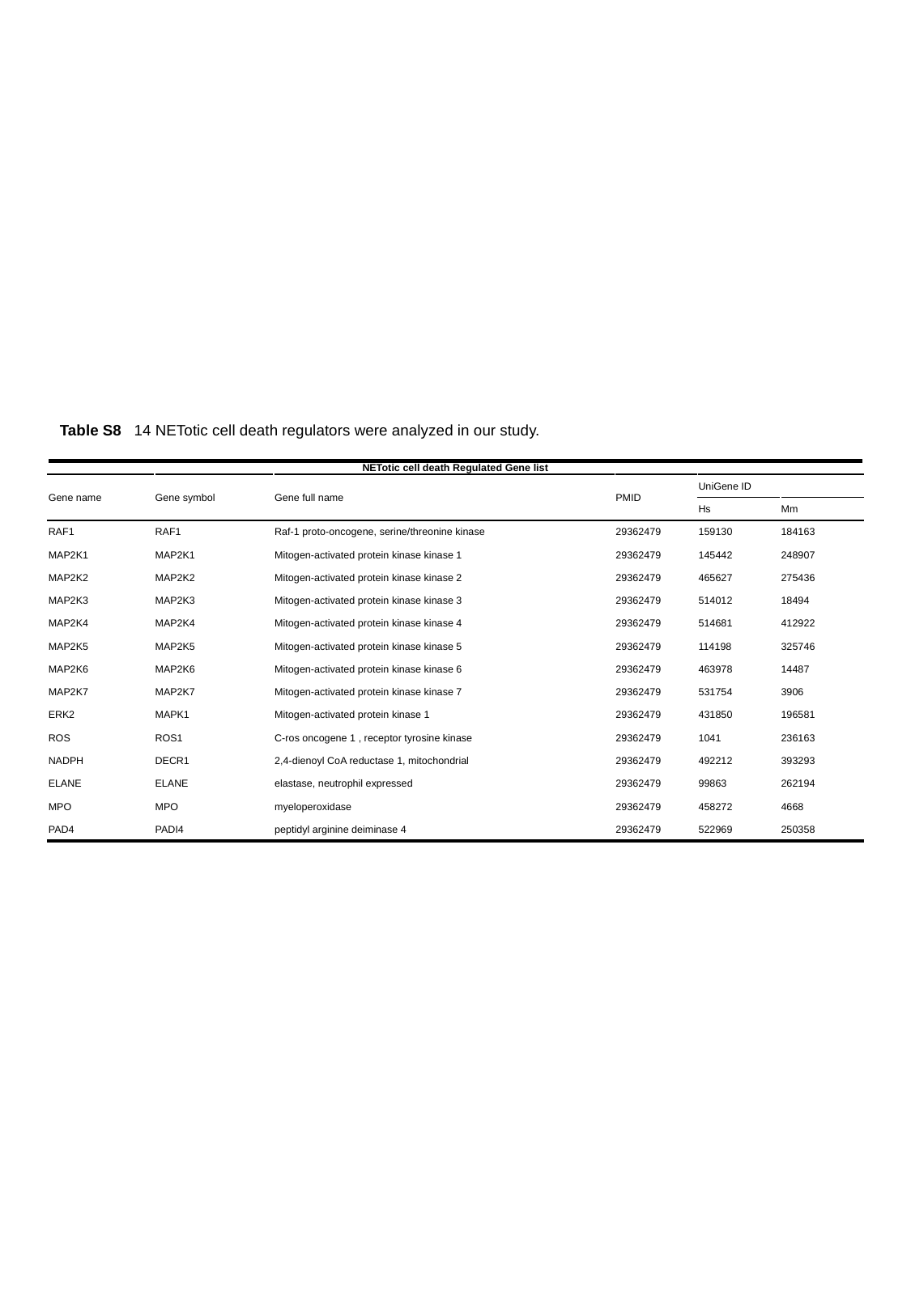

Table S8 14 NETotic cell death regulators were analyzed in our study.
| NETotic cell death Regulated Gene list | | | | | |
| --- | --- | --- | --- | --- | --- |
| Gene name | Gene symbol | Gene full name | PMID | UniGene ID | |
| | | | | Hs | Mm |
| RAF1 | RAF1 | Raf-1 proto-oncogene, serine/threonine kinase | 29362479 | 159130 | 184163 |
| MAP2K1 | MAP2K1 | Mitogen-activated protein kinase kinase 1 | 29362479 | 145442 | 248907 |
| MAP2K2 | MAP2K2 | Mitogen-activated protein kinase kinase 2 | 29362479 | 465627 | 275436 |
| MAP2K3 | MAP2K3 | Mitogen-activated protein kinase kinase 3 | 29362479 | 514012 | 18494 |
| MAP2K4 | MAP2K4 | Mitogen-activated protein kinase kinase 4 | 29362479 | 514681 | 412922 |
| MAP2K5 | MAP2K5 | Mitogen-activated protein kinase kinase 5 | 29362479 | 114198 | 325746 |
| MAP2K6 | MAP2K6 | Mitogen-activated protein kinase kinase 6 | 29362479 | 463978 | 14487 |
| MAP2K7 | MAP2K7 | Mitogen-activated protein kinase kinase 7 | 29362479 | 531754 | 3906 |
| ERK2 | MAPK1 | Mitogen-activated protein kinase 1 | 29362479 | 431850 | 196581 |
| ROS | ROS1 | C-ros oncogene 1 , receptor tyrosine kinase | 29362479 | 1041 | 236163 |
| NADPH | DECR1 | 2,4-dienoyl CoA reductase 1, mitochondrial | 29362479 | 492212 | 393293 |
| ELANE | ELANE | elastase, neutrophil expressed | 29362479 | 99863 | 262194 |
| MPO | MPO | myeloperoxidase | 29362479 | 458272 | 4668 |
| PAD4 | PADI4 | peptidyl arginine deiminase 4 | 29362479 | 522969 | 250358 |

## Slide 10
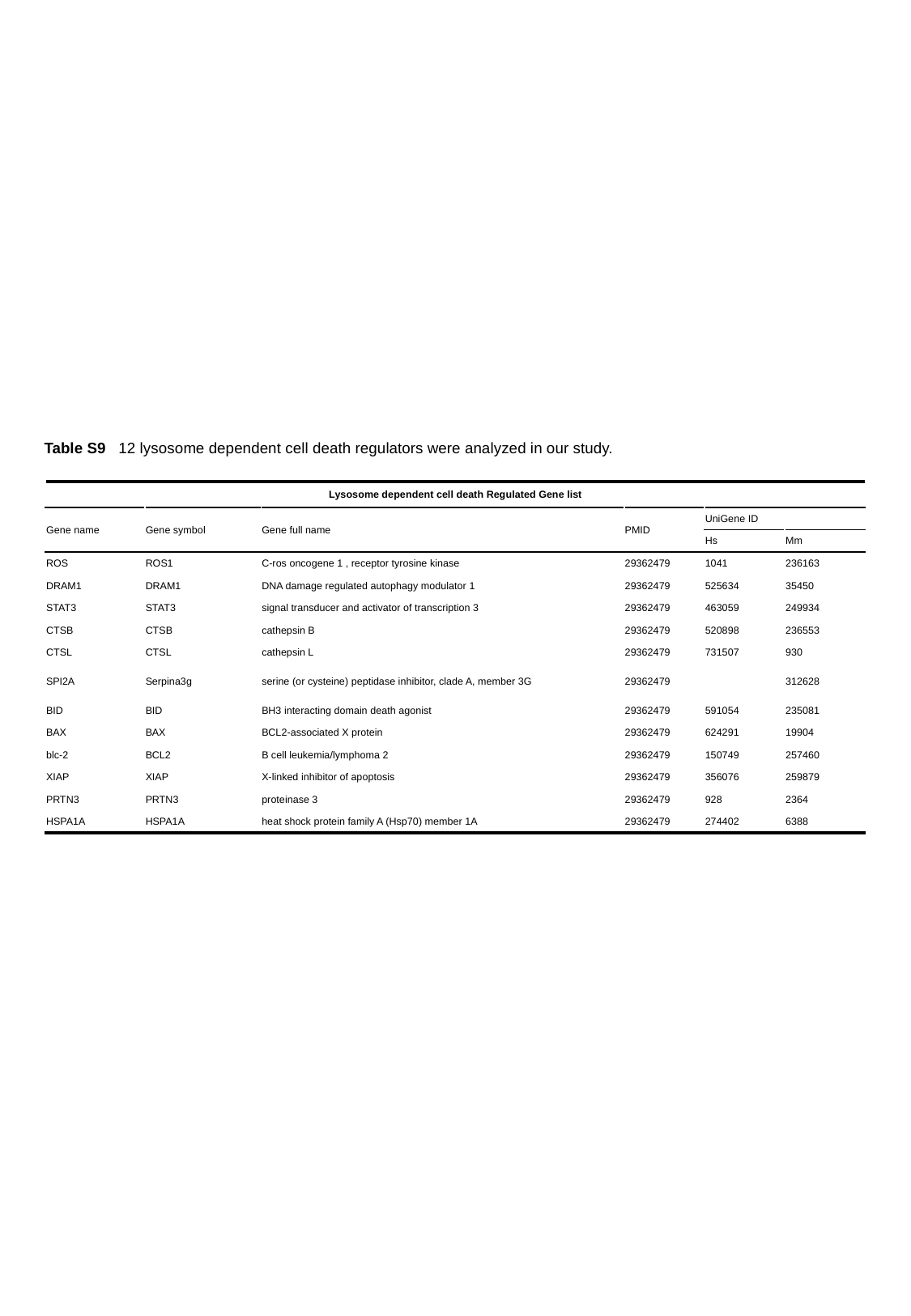

Table S9 12 lysosome dependent cell death regulators were analyzed in our study.
| Lysosome dependent cell death Regulated Gene list | | | | | |
| --- | --- | --- | --- | --- | --- |
| Gene name | Gene symbol | Gene full name | PMID | UniGene ID | |
| | | | | Hs | Mm |
| ROS | ROS1 | C-ros oncogene 1 , receptor tyrosine kinase | 29362479 | 1041 | 236163 |
| DRAM1 | DRAM1 | DNA damage regulated autophagy modulator 1 | 29362479 | 525634 | 35450 |
| STAT3 | STAT3 | signal transducer and activator of transcription 3 | 29362479 | 463059 | 249934 |
| CTSB | CTSB | cathepsin B | 29362479 | 520898 | 236553 |
| CTSL | CTSL | cathepsin L | 29362479 | 731507 | 930 |
| SPI2A | Serpina3g | serine (or cysteine) peptidase inhibitor, clade A, member 3G | 29362479 | | 312628 |
| BID | BID | BH3 interacting domain death agonist | 29362479 | 591054 | 235081 |
| BAX | BAX | BCL2-associated X protein | 29362479 | 624291 | 19904 |
| blc-2 | BCL2 | B cell leukemia/lymphoma 2 | 29362479 | 150749 | 257460 |
| XIAP | XIAP | X-linked inhibitor of apoptosis | 29362479 | 356076 | 259879 |
| PRTN3 | PRTN3 | proteinase 3 | 29362479 | 928 | 2364 |
| HSPA1A | HSPA1A | heat shock protein family A (Hsp70) member 1A | 29362479 | 274402 | 6388 |

## Slide 11
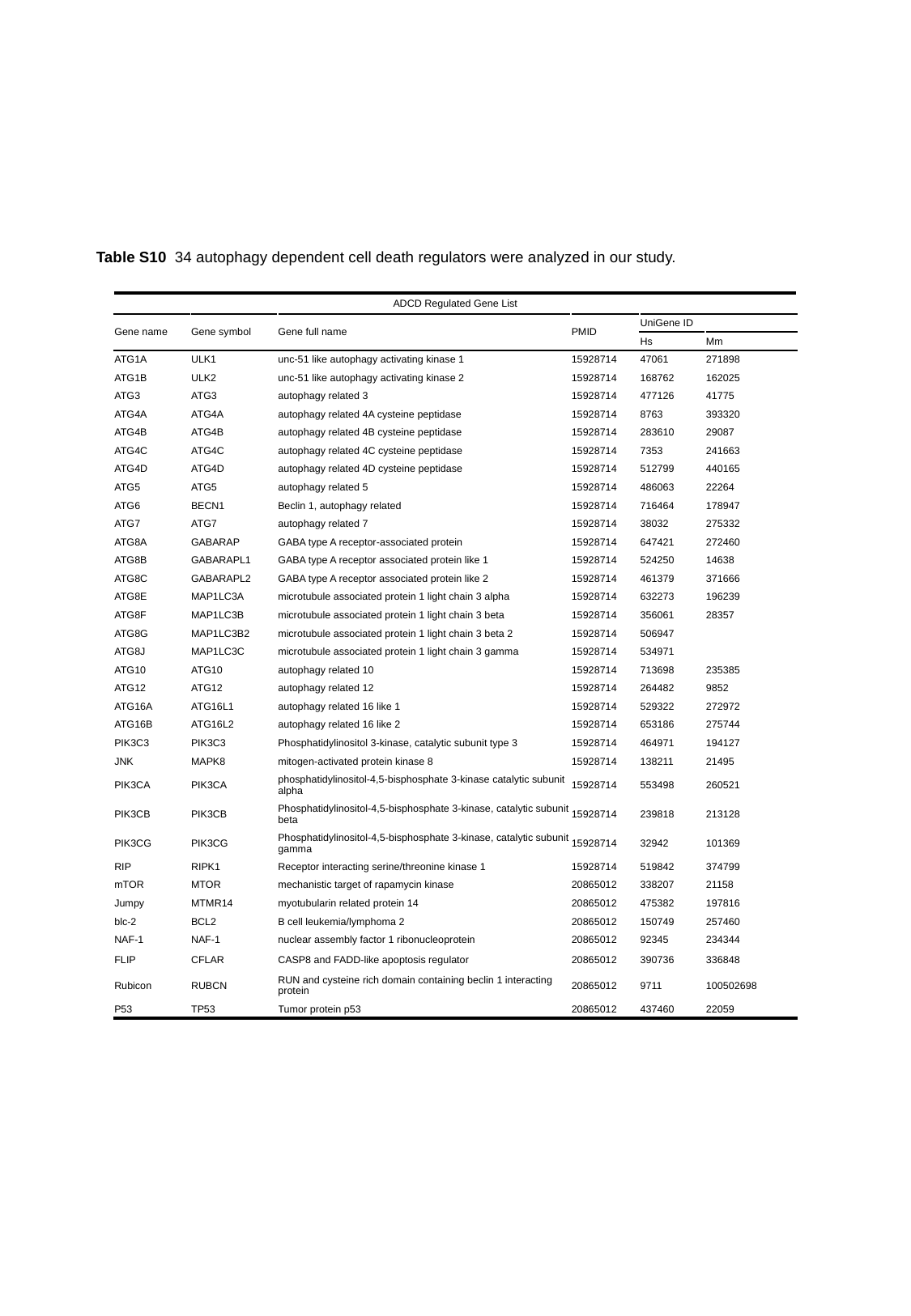

Table S10 34 autophagy dependent cell death regulators were analyzed in our study.
| ADCD Regulated Gene List | | | | | |
| --- | --- | --- | --- | --- | --- |
| Gene name | Gene symbol | Gene full name | PMID | UniGene ID | |
| | | | | Hs | Mm |
| ATG1A | ULK1 | unc-51 like autophagy activating kinase 1 | 15928714 | 47061 | 271898 |
| ATG1B | ULK2 | unc-51 like autophagy activating kinase 2 | 15928714 | 168762 | 162025 |
| ATG3 | ATG3 | autophagy related 3 | 15928714 | 477126 | 41775 |
| ATG4A | ATG4A | autophagy related 4A cysteine peptidase | 15928714 | 8763 | 393320 |
| ATG4B | ATG4B | autophagy related 4B cysteine peptidase | 15928714 | 283610 | 29087 |
| ATG4C | ATG4C | autophagy related 4C cysteine peptidase | 15928714 | 7353 | 241663 |
| ATG4D | ATG4D | autophagy related 4D cysteine peptidase | 15928714 | 512799 | 440165 |
| ATG5 | ATG5 | autophagy related 5 | 15928714 | 486063 | 22264 |
| ATG6 | BECN1 | Beclin 1, autophagy related | 15928714 | 716464 | 178947 |
| ATG7 | ATG7 | autophagy related 7 | 15928714 | 38032 | 275332 |
| ATG8A | GABARAP | GABA type A receptor-associated protein | 15928714 | 647421 | 272460 |
| ATG8B | GABARAPL1 | GABA type A receptor associated protein like 1 | 15928714 | 524250 | 14638 |
| ATG8C | GABARAPL2 | GABA type A receptor associated protein like 2 | 15928714 | 461379 | 371666 |
| ATG8E | MAP1LC3A | microtubule associated protein 1 light chain 3 alpha | 15928714 | 632273 | 196239 |
| ATG8F | MAP1LC3B | microtubule associated protein 1 light chain 3 beta | 15928714 | 356061 | 28357 |
| ATG8G | MAP1LC3B2 | microtubule associated protein 1 light chain 3 beta 2 | 15928714 | 506947 | |
| ATG8J | MAP1LC3C | microtubule associated protein 1 light chain 3 gamma | 15928714 | 534971 | |
| ATG10 | ATG10 | autophagy related 10 | 15928714 | 713698 | 235385 |
| ATG12 | ATG12 | autophagy related 12 | 15928714 | 264482 | 9852 |
| ATG16A | ATG16L1 | autophagy related 16 like 1 | 15928714 | 529322 | 272972 |
| ATG16B | ATG16L2 | autophagy related 16 like 2 | 15928714 | 653186 | 275744 |
| PIK3C3 | PIK3C3 | Phosphatidylinositol 3-kinase, catalytic subunit type 3 | 15928714 | 464971 | 194127 |
| JNK | MAPK8 | mitogen-activated protein kinase 8 | 15928714 | 138211 | 21495 |
| PIK3CA | PIK3CA | phosphatidylinositol-4,5-bisphosphate 3-kinase catalytic subunit alpha | 15928714 | 553498 | 260521 |
| PIK3CB | PIK3CB | Phosphatidylinositol-4,5-bisphosphate 3-kinase, catalytic subunit beta | 15928714 | 239818 | 213128 |
| PIK3CG | PIK3CG | Phosphatidylinositol-4,5-bisphosphate 3-kinase, catalytic subunit gamma | 15928714 | 32942 | 101369 |
| RIP | RIPK1 | Receptor interacting serine/threonine kinase 1 | 15928714 | 519842 | 374799 |
| mTOR | MTOR | mechanistic target of rapamycin kinase | 20865012 | 338207 | 21158 |
| Jumpy | MTMR14 | myotubularin related protein 14 | 20865012 | 475382 | 197816 |
| blc-2 | BCL2 | B cell leukemia/lymphoma 2 | 20865012 | 150749 | 257460 |
| NAF-1 | NAF-1 | nuclear assembly factor 1 ribonucleoprotein | 20865012 | 92345 | 234344 |
| FLIP | CFLAR | CASP8 and FADD-like apoptosis regulator | 20865012 | 390736 | 336848 |
| Rubicon | RUBCN | RUN and cysteine rich domain containing beclin 1 interacting protein | 20865012 | 9711 | 100502698 |
| P53 | TP53 | Tumor protein p53 | 20865012 | 437460 | 22059 |

## Slide 12
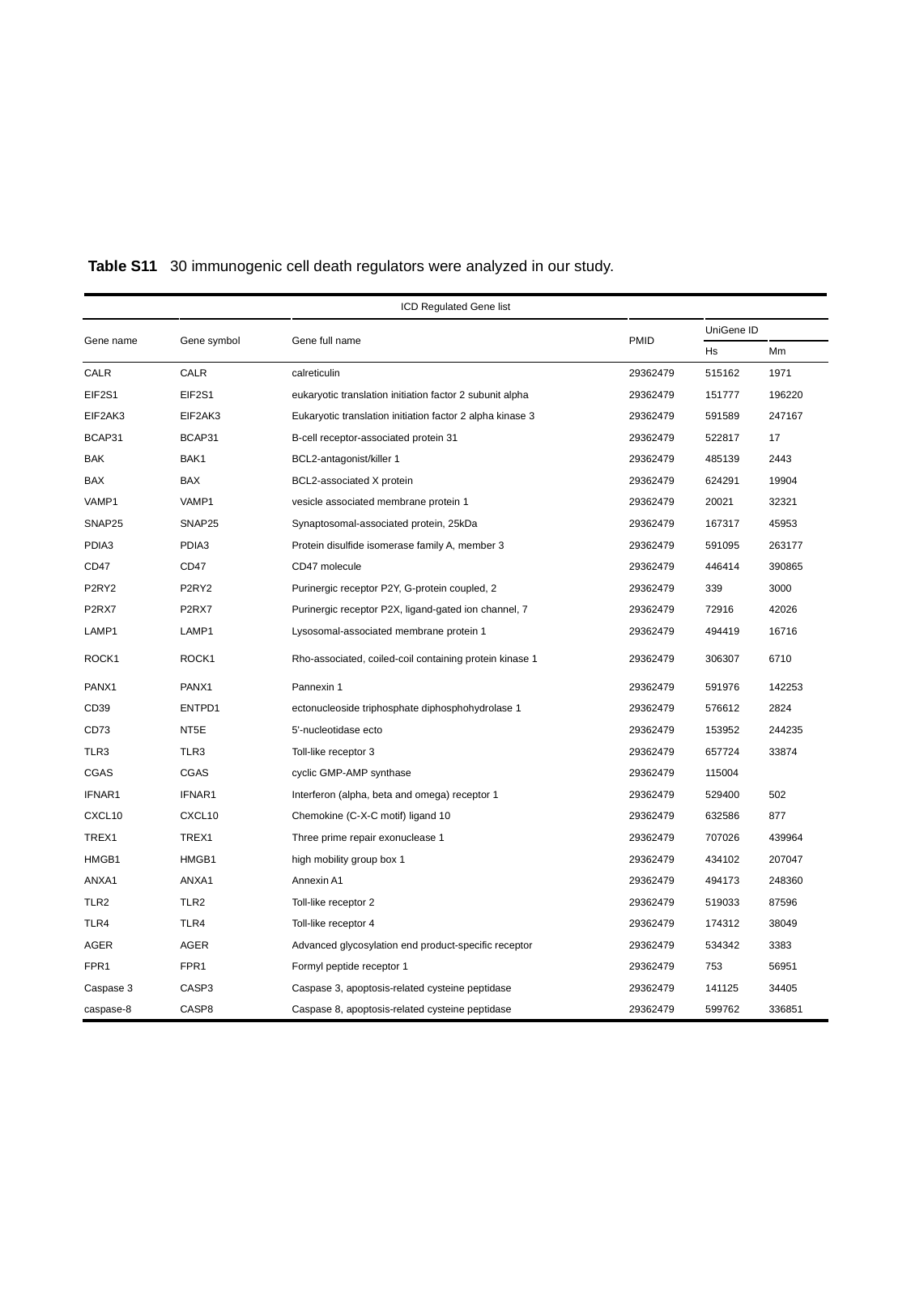

Table S11 30 immunogenic cell death regulators were analyzed in our study.
| ICD Regulated Gene list | | | | | |
| --- | --- | --- | --- | --- | --- |
| Gene name | Gene symbol | Gene full name | PMID | UniGene ID | |
| | | | | Hs | Mm |
| CALR | CALR | calreticulin | 29362479 | 515162 | 1971 |
| EIF2S1 | EIF2S1 | eukaryotic translation initiation factor 2 subunit alpha | 29362479 | 151777 | 196220 |
| EIF2AK3 | EIF2AK3 | Eukaryotic translation initiation factor 2 alpha kinase 3 | 29362479 | 591589 | 247167 |
| BCAP31 | BCAP31 | B-cell receptor-associated protein 31 | 29362479 | 522817 | 17 |
| BAK | BAK1 | BCL2-antagonist/killer 1 | 29362479 | 485139 | 2443 |
| BAX | BAX | BCL2-associated X protein | 29362479 | 624291 | 19904 |
| VAMP1 | VAMP1 | vesicle associated membrane protein 1 | 29362479 | 20021 | 32321 |
| SNAP25 | SNAP25 | Synaptosomal-associated protein, 25kDa | 29362479 | 167317 | 45953 |
| PDIA3 | PDIA3 | Protein disulfide isomerase family A, member 3 | 29362479 | 591095 | 263177 |
| CD47 | CD47 | CD47 molecule | 29362479 | 446414 | 390865 |
| P2RY2 | P2RY2 | Purinergic receptor P2Y, G-protein coupled, 2 | 29362479 | 339 | 3000 |
| P2RX7 | P2RX7 | Purinergic receptor P2X, ligand-gated ion channel, 7 | 29362479 | 72916 | 42026 |
| LAMP1 | LAMP1 | Lysosomal-associated membrane protein 1 | 29362479 | 494419 | 16716 |
| ROCK1 | ROCK1 | Rho-associated, coiled-coil containing protein kinase 1 | 29362479 | 306307 | 6710 |
| PANX1 | PANX1 | Pannexin 1 | 29362479 | 591976 | 142253 |
| CD39 | ENTPD1 | ectonucleoside triphosphate diphosphohydrolase 1 | 29362479 | 576612 | 2824 |
| CD73 | NT5E | 5'-nucleotidase ecto | 29362479 | 153952 | 244235 |
| TLR3 | TLR3 | Toll-like receptor 3 | 29362479 | 657724 | 33874 |
| CGAS | CGAS | cyclic GMP-AMP synthase | 29362479 | 115004 | |
| IFNAR1 | IFNAR1 | Interferon (alpha, beta and omega) receptor 1 | 29362479 | 529400 | 502 |
| CXCL10 | CXCL10 | Chemokine (C-X-C motif) ligand 10 | 29362479 | 632586 | 877 |
| TREX1 | TREX1 | Three prime repair exonuclease 1 | 29362479 | 707026 | 439964 |
| HMGB1 | HMGB1 | high mobility group box 1 | 29362479 | 434102 | 207047 |
| ANXA1 | ANXA1 | Annexin A1 | 29362479 | 494173 | 248360 |
| TLR2 | TLR2 | Toll-like receptor 2 | 29362479 | 519033 | 87596 |
| TLR4 | TLR4 | Toll-like receptor 4 | 29362479 | 174312 | 38049 |
| AGER | AGER | Advanced glycosylation end product-specific receptor | 29362479 | 534342 | 3383 |
| FPR1 | FPR1 | Formyl peptide receptor 1 | 29362479 | 753 | 56951 |
| Caspase 3 | CASP3 | Caspase 3, apoptosis-related cysteine peptidase | 29362479 | 141125 | 34405 |
| caspase-8 | CASP8 | Caspase 8, apoptosis-related cysteine peptidase | 29362479 | 599762 | 336851 |

## Slide 13
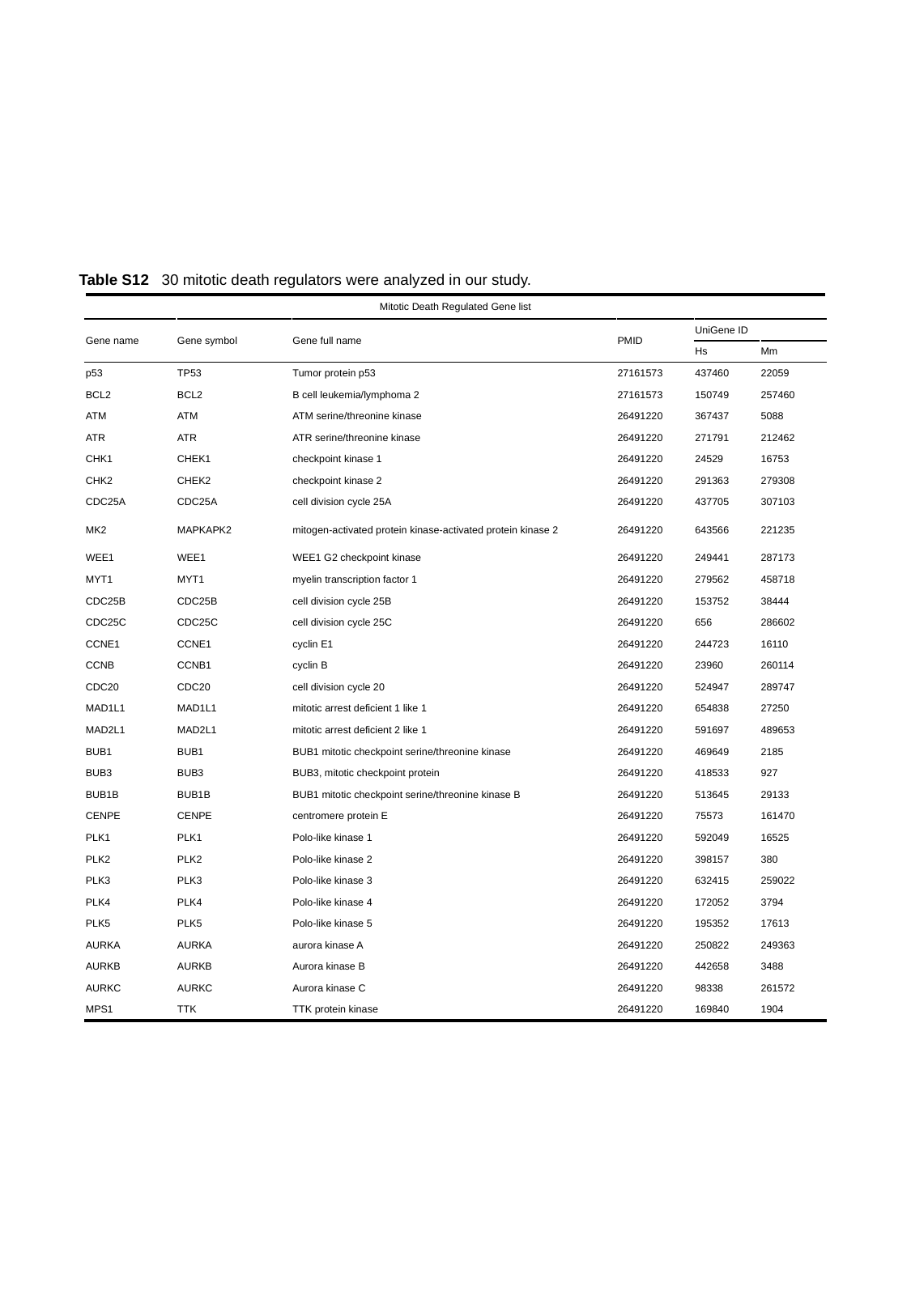

Table S12 30 mitotic death regulators were analyzed in our study.
| Mitotic Death Regulated Gene list | | | | | |
| --- | --- | --- | --- | --- | --- |
| Gene name | Gene symbol | Gene full name | PMID | UniGene ID | |
| | | | | Hs | Mm |
| p53 | TP53 | Tumor protein p53 | 27161573 | 437460 | 22059 |
| BCL2 | BCL2 | B cell leukemia/lymphoma 2 | 27161573 | 150749 | 257460 |
| ATM | ATM | ATM serine/threonine kinase | 26491220 | 367437 | 5088 |
| ATR | ATR | ATR serine/threonine kinase | 26491220 | 271791 | 212462 |
| CHK1 | CHEK1 | checkpoint kinase 1 | 26491220 | 24529 | 16753 |
| CHK2 | CHEK2 | checkpoint kinase 2 | 26491220 | 291363 | 279308 |
| CDC25A | CDC25A | cell division cycle 25A | 26491220 | 437705 | 307103 |
| MK2 | MAPKAPK2 | mitogen-activated protein kinase-activated protein kinase 2 | 26491220 | 643566 | 221235 |
| WEE1 | WEE1 | WEE1 G2 checkpoint kinase | 26491220 | 249441 | 287173 |
| MYT1 | MYT1 | myelin transcription factor 1 | 26491220 | 279562 | 458718 |
| CDC25B | CDC25B | cell division cycle 25B | 26491220 | 153752 | 38444 |
| CDC25C | CDC25C | cell division cycle 25C | 26491220 | 656 | 286602 |
| CCNE1 | CCNE1 | cyclin E1 | 26491220 | 244723 | 16110 |
| CCNB | CCNB1 | cyclin B | 26491220 | 23960 | 260114 |
| CDC20 | CDC20 | cell division cycle 20 | 26491220 | 524947 | 289747 |
| MAD1L1 | MAD1L1 | mitotic arrest deficient 1 like 1 | 26491220 | 654838 | 27250 |
| MAD2L1 | MAD2L1 | mitotic arrest deficient 2 like 1 | 26491220 | 591697 | 489653 |
| BUB1 | BUB1 | BUB1 mitotic checkpoint serine/threonine kinase | 26491220 | 469649 | 2185 |
| BUB3 | BUB3 | BUB3, mitotic checkpoint protein | 26491220 | 418533 | 927 |
| BUB1B | BUB1B | BUB1 mitotic checkpoint serine/threonine kinase B | 26491220 | 513645 | 29133 |
| CENPE | CENPE | centromere protein E | 26491220 | 75573 | 161470 |
| PLK1 | PLK1 | Polo-like kinase 1 | 26491220 | 592049 | 16525 |
| PLK2 | PLK2 | Polo-like kinase 2 | 26491220 | 398157 | 380 |
| PLK3 | PLK3 | Polo-like kinase 3 | 26491220 | 632415 | 259022 |
| PLK4 | PLK4 | Polo-like kinase 4 | 26491220 | 172052 | 3794 |
| PLK5 | PLK5 | Polo-like kinase 5 | 26491220 | 195352 | 17613 |
| AURKA | AURKA | aurora kinase A | 26491220 | 250822 | 249363 |
| AURKB | AURKB | Aurora kinase B | 26491220 | 442658 | 3488 |
| AURKC | AURKC | Aurora kinase C | 26491220 | 98338 | 261572 |
| MPS1 | TTK | TTK protein kinase | 26491220 | 169840 | 1904 |

## Slide 14
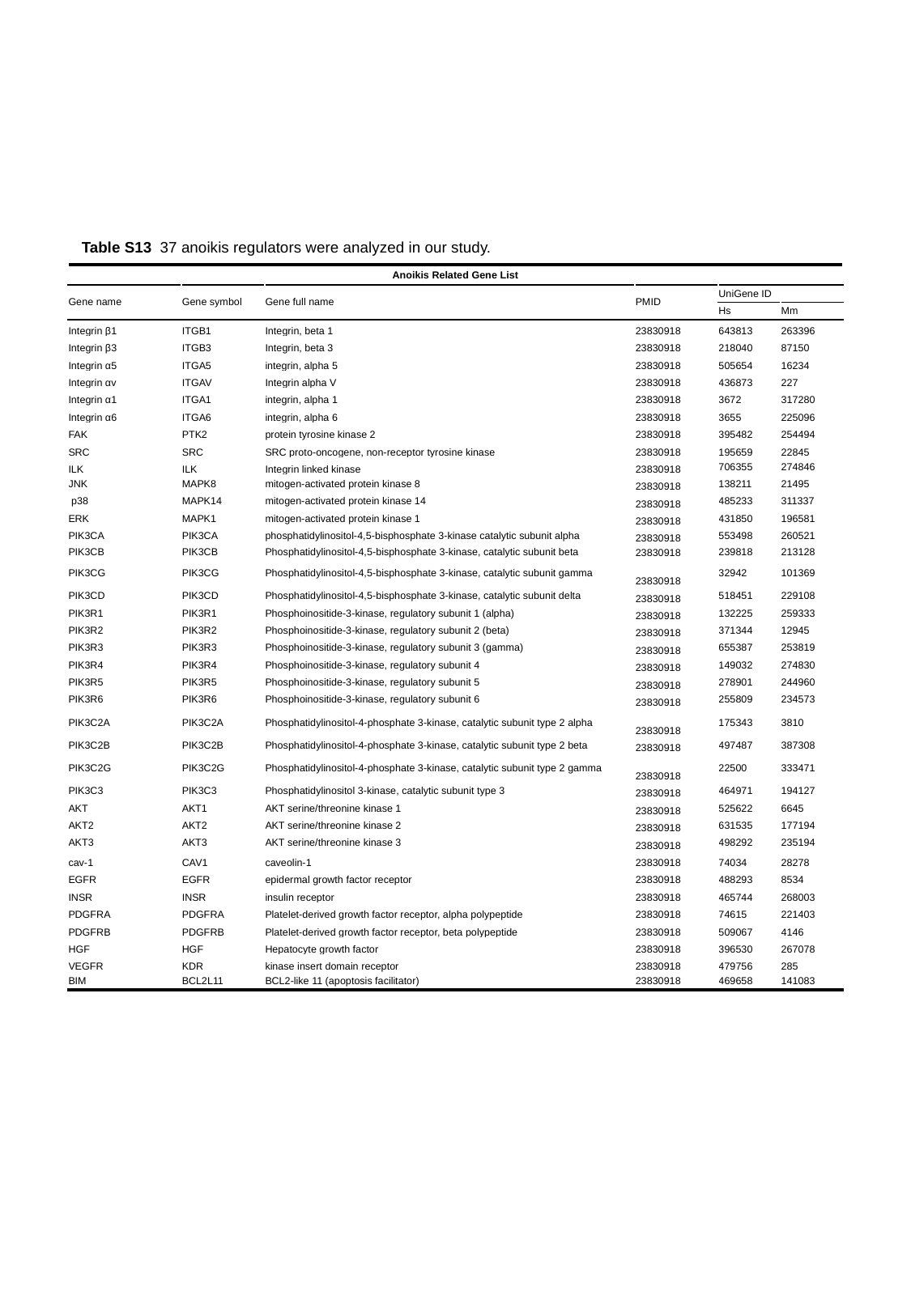

Table S13 37 anoikis regulators were analyzed in our study.
| Anoikis Related Gene List | | | | | |
| --- | --- | --- | --- | --- | --- |
| Gene name | Gene symbol | Gene full name | PMID | UniGene ID | |
| | | | | Hs | Mm |
| Integrin β1 | ITGB1 | Integrin, beta 1 | 23830918 | 643813 | 263396 |
| Integrin β3 | ITGB3 | Integrin, beta 3 | 23830918 | 218040 | 87150 |
| Integrin α5 | ITGA5 | integrin, alpha 5 | 23830918 | 505654 | 16234 |
| Integrin αv | ITGAV | Integrin alpha V | 23830918 | 436873 | 227 |
| Integrin α1 | ITGA1 | integrin, alpha 1 | 23830918 | 3672 | 317280 |
| Integrin α6 | ITGA6 | integrin, alpha 6 | 23830918 | 3655 | 225096 |
| FAK | PTK2 | protein tyrosine kinase 2 | 23830918 | 395482 | 254494 |
| SRC | SRC | SRC proto-oncogene, non-receptor tyrosine kinase | 23830918 | 195659 | 22845 |
| ILK | ILK | Integrin linked kinase | 23830918 | 706355 | 274846 |
| JNK | MAPK8 | mitogen-activated protein kinase 8 | 23830918 | 138211 | 21495 |
| p38 | MAPK14 | mitogen-activated protein kinase 14 | 23830918 | 485233 | 311337 |
| ERK | MAPK1 | mitogen-activated protein kinase 1 | 23830918 | 431850 | 196581 |
| PIK3CA | PIK3CA | phosphatidylinositol-4,5-bisphosphate 3-kinase catalytic subunit alpha | 23830918 | 553498 | 260521 |
| PIK3CB | PIK3CB | Phosphatidylinositol-4,5-bisphosphate 3-kinase, catalytic subunit beta | 23830918 | 239818 | 213128 |
| PIK3CG | PIK3CG | Phosphatidylinositol-4,5-bisphosphate 3-kinase, catalytic subunit gamma | 23830918 | 32942 | 101369 |
| PIK3CD | PIK3CD | Phosphatidylinositol-4,5-bisphosphate 3-kinase, catalytic subunit delta | 23830918 | 518451 | 229108 |
| PIK3R1 | PIK3R1 | Phosphoinositide-3-kinase, regulatory subunit 1 (alpha) | 23830918 | 132225 | 259333 |
| PIK3R2 | PIK3R2 | Phosphoinositide-3-kinase, regulatory subunit 2 (beta) | 23830918 | 371344 | 12945 |
| PIK3R3 | PIK3R3 | Phosphoinositide-3-kinase, regulatory subunit 3 (gamma) | 23830918 | 655387 | 253819 |
| PIK3R4 | PIK3R4 | Phosphoinositide-3-kinase, regulatory subunit 4 | 23830918 | 149032 | 274830 |
| PIK3R5 | PIK3R5 | Phosphoinositide-3-kinase, regulatory subunit 5 | 23830918 | 278901 | 244960 |
| PIK3R6 | PIK3R6 | Phosphoinositide-3-kinase, regulatory subunit 6 | 23830918 | 255809 | 234573 |
| PIK3C2A | PIK3C2A | Phosphatidylinositol-4-phosphate 3-kinase, catalytic subunit type 2 alpha | 23830918 | 175343 | 3810 |
| PIK3C2B | PIK3C2B | Phosphatidylinositol-4-phosphate 3-kinase, catalytic subunit type 2 beta | 23830918 | 497487 | 387308 |
| PIK3C2G | PIK3C2G | Phosphatidylinositol-4-phosphate 3-kinase, catalytic subunit type 2 gamma | 23830918 | 22500 | 333471 |
| PIK3C3 | PIK3C3 | Phosphatidylinositol 3-kinase, catalytic subunit type 3 | 23830918 | 464971 | 194127 |
| AKT | AKT1 | AKT serine/threonine kinase 1 | 23830918 | 525622 | 6645 |
| AKT2 | AKT2 | AKT serine/threonine kinase 2 | 23830918 | 631535 | 177194 |
| AKT3 | AKT3 | AKT serine/threonine kinase 3 | 23830918 | 498292 | 235194 |
| cav-1 | CAV1 | caveolin-1 | 23830918 | 74034 | 28278 |
| EGFR | EGFR | epidermal growth factor receptor | 23830918 | 488293 | 8534 |
| INSR | INSR | insulin receptor | 23830918 | 465744 | 268003 |
| PDGFRA | PDGFRA | Platelet-derived growth factor receptor, alpha polypeptide | 23830918 | 74615 | 221403 |
| PDGFRB | PDGFRB | Platelet-derived growth factor receptor, beta polypeptide | 23830918 | 509067 | 4146 |
| HGF | HGF | Hepatocyte growth factor | 23830918 | 396530 | 267078 |
| VEGFR | KDR | kinase insert domain receptor | 23830918 | 479756 | 285 |
| BIM | BCL2L11 | BCL2-like 11 (apoptosis facilitator) | 23830918 | 469658 | 141083 |

## Slide 15
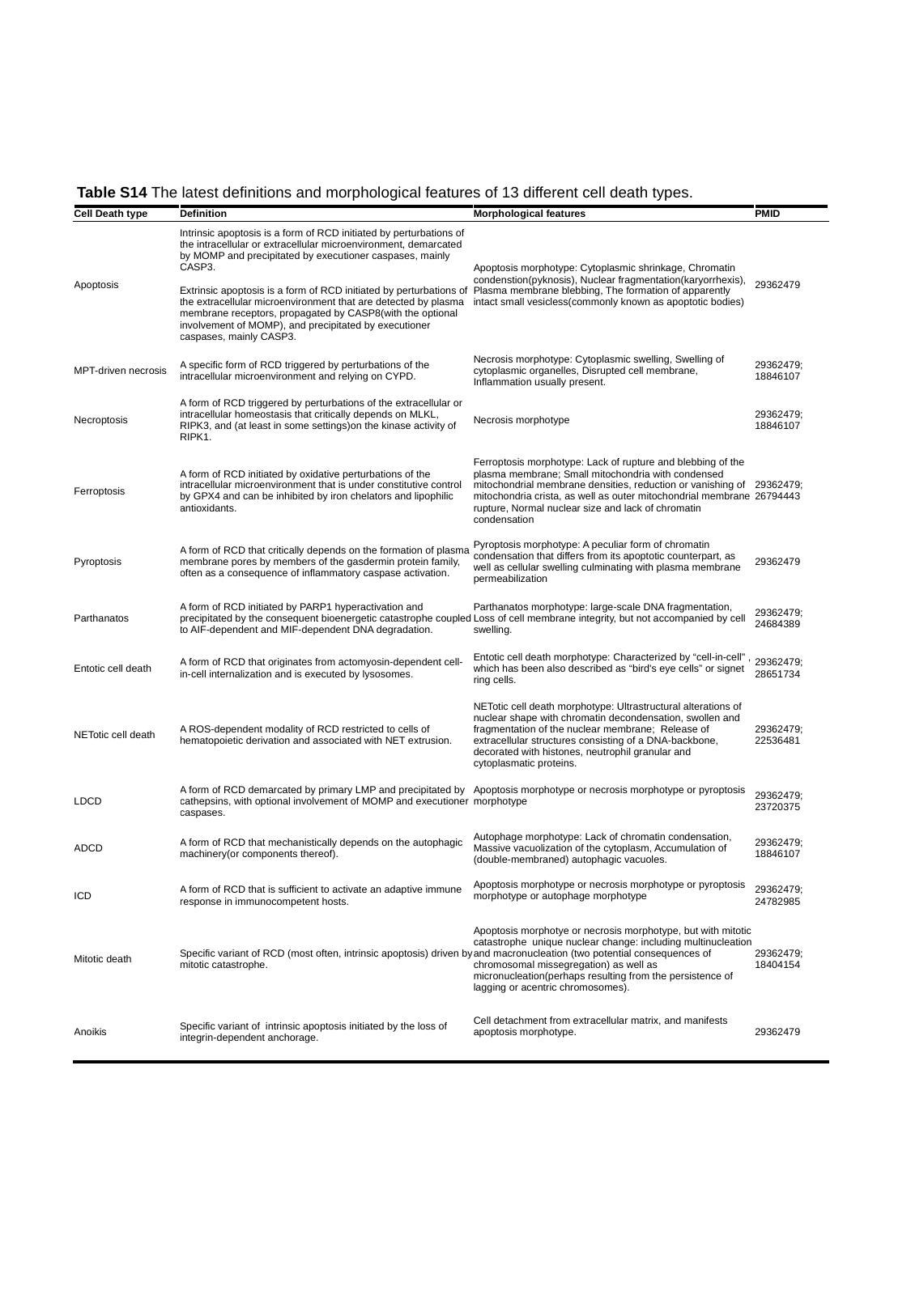

Table S14 The latest definitions and morphological features of 13 different cell death types.
| Cell Death type | Definition | Morphological features | PMID |
| --- | --- | --- | --- |
| Apoptosis | Intrinsic apoptosis is a form of RCD initiated by perturbations of the intracellular or extracellular microenvironment, demarcated by MOMP and precipitated by executioner caspases, mainly CASP3. | Apoptosis morphotype: Cytoplasmic shrinkage, Chromatin condenstion(pyknosis), Nuclear fragmentation(karyorrhexis), Plasma membrane blebbing, The formation of apparently intact small vesicless(commonly known as apoptotic bodies) | 29362479 |
| | Extrinsic apoptosis is a form of RCD initiated by perturbations of the extracellular microenvironment that are detected by plasma membrane receptors, propagated by CASP8(with the optional involvement of MOMP), and precipitated by executioner caspases, mainly CASP3. | | |
| MPT-driven necrosis | A specific form of RCD triggered by perturbations of the intracellular microenvironment and relying on CYPD. | Necrosis morphotype: Cytoplasmic swelling, Swelling of cytoplasmic organelles, Disrupted cell membrane, Inflammation usually present. | 29362479; 18846107 |
| Necroptosis | A form of RCD triggered by perturbations of the extracellular or intracellular homeostasis that critically depends on MLKL, RIPK3, and (at least in some settings)on the kinase activity of RIPK1. | Necrosis morphotype | 29362479; 18846107 |
| Ferroptosis | A form of RCD initiated by oxidative perturbations of the intracellular microenvironment that is under constitutive control by GPX4 and can be inhibited by iron chelators and lipophilic antioxidants. | Ferroptosis morphotype: Lack of rupture and blebbing of the plasma membrane; Small mitochondria with condensed mitochondrial membrane densities, reduction or vanishing of mitochondria crista, as well as outer mitochondrial membrane rupture, Normal nuclear size and lack of chromatin condensation | 29362479; 26794443 |
| Pyroptosis | A form of RCD that critically depends on the formation of plasma membrane pores by members of the gasdermin protein family, often as a consequence of inflammatory caspase activation. | Pyroptosis morphotype: A peculiar form of chromatin condensation that differs from its apoptotic counterpart, as well as cellular swelling culminating with plasma membrane permeabilization | 29362479 |
| Parthanatos | A form of RCD initiated by PARP1 hyperactivation and precipitated by the consequent bioenergetic catastrophe coupled to AIF-dependent and MIF-dependent DNA degradation. | Parthanatos morphotype: large-scale DNA fragmentation, Loss of cell membrane integrity, but not accompanied by cell swelling. | 29362479; 24684389 |
| Entotic cell death | A form of RCD that originates from actomyosin-dependent cell-in-cell internalization and is executed by lysosomes. | Entotic cell death morphotype: Characterized by “cell-in-cell" , which has been also described as “bird's eye cells” or signet ring cells. | 29362479; 28651734 |
| NETotic cell death | A ROS-dependent modality of RCD restricted to cells of hematopoietic derivation and associated with NET extrusion. | NETotic cell death morphotype: Ultrastructural alterations of nuclear shape with chromatin decondensation, swollen and fragmentation of the nuclear membrane; Release of extracellular structures consisting of a DNA-backbone, decorated with histones, neutrophil granular and cytoplasmatic proteins. | 29362479; 22536481 |
| LDCD | A form of RCD demarcated by primary LMP and precipitated by cathepsins, with optional involvement of MOMP and executioner caspases. | Apoptosis morphotype or necrosis morphotype or pyroptosis morphotype | 29362479; 23720375 |
| ADCD | A form of RCD that mechanistically depends on the autophagic machinery(or components thereof). | Autophage morphotype: Lack of chromatin condensation, Massive vacuolization of the cytoplasm, Accumulation of (double-membraned) autophagic vacuoles. | 29362479; 18846107 |
| ICD | A form of RCD that is sufficient to activate an adaptive immune response in immunocompetent hosts. | Apoptosis morphotype or necrosis morphotype or pyroptosis morphotype or autophage morphotype | 29362479; 24782985 |
| Mitotic death | Specific variant of RCD (most often, intrinsic apoptosis) driven by mitotic catastrophe. | Apoptosis morphotye or necrosis morphotype, but with mitotic catastrophe unique nuclear change: including multinucleation and macronucleation (two potential consequences of chromosomal missegregation) as well as micronucleation(perhaps resulting from the persistence of lagging or acentric chromosomes). | 29362479; 18404154 |
| Anoikis | Specific variant of intrinsic apoptosis initiated by the loss of integrin-dependent anchorage. | Cell detachment from extracellular matrix, and manifests apoptosis morphotype. | 29362479 |

## Slide 16
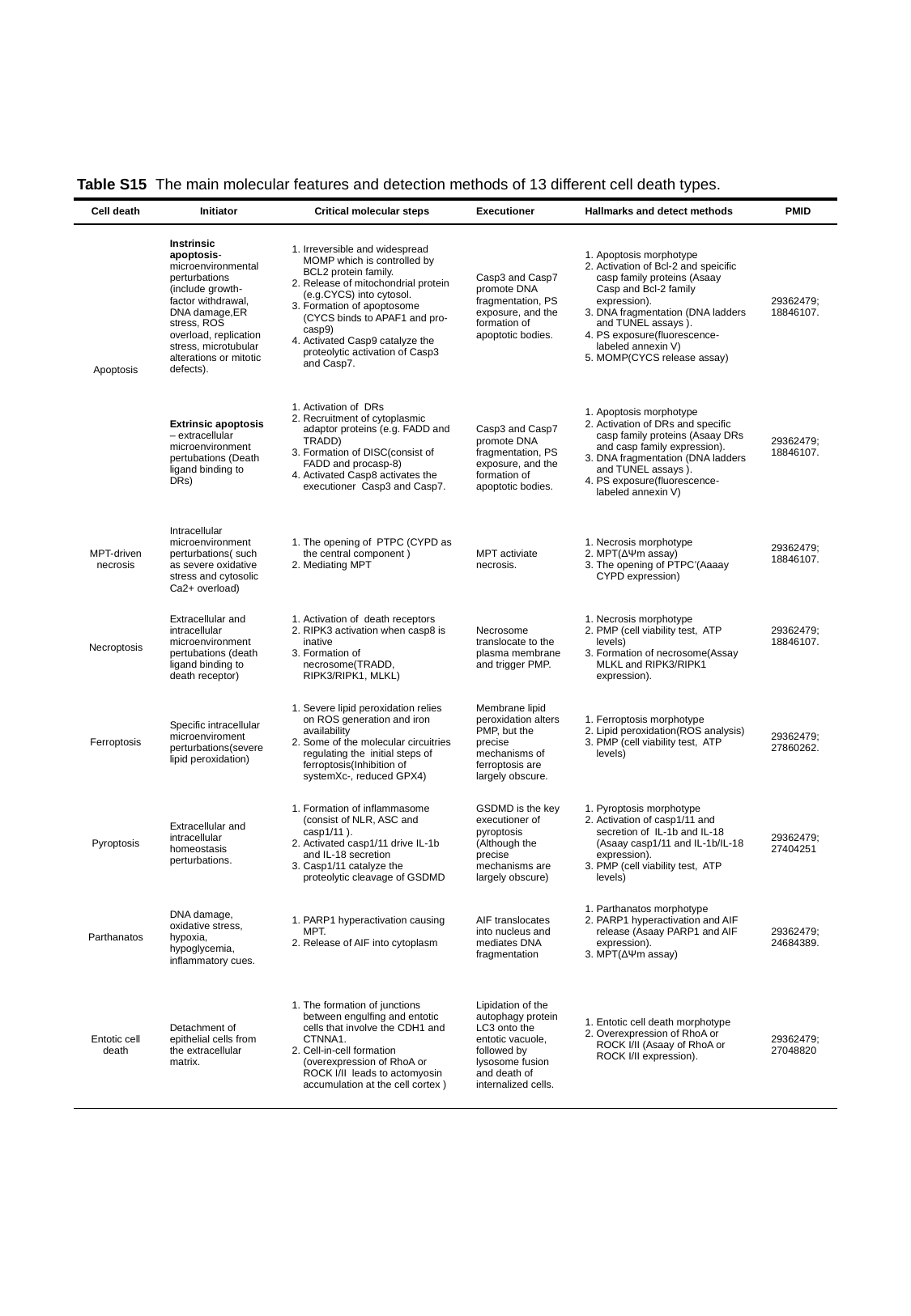

Table S15 The main molecular features and detection methods of 13 different cell death types.
| Cell death | Initiator | Critical molecular steps | Executioner | Hallmarks and detect methods | PMID |
| --- | --- | --- | --- | --- | --- |
| Apoptosis | Instrinsic apoptosis-microenvironmental perturbations (include growth-factor withdrawal, DNA damage,ER stress, ROS overload, replication stress, microtubular alterations or mitotic defects). | Irreversible and widespread MOMP which is controlled by BCL2 protein family. Release of mitochondrial protein (e.g.CYCS) into cytosol. Formation of apoptosome (CYCS binds to APAF1 and pro-casp9) Activated Casp9 catalyze the proteolytic activation of Casp3 and Casp7. | Casp3 and Casp7 promote DNA fragmentation, PS exposure, and the formation of apoptotic bodies. | Apoptosis morphotype Activation of Bcl-2 and speicific casp family proteins (Asaay Casp and Bcl-2 family expression). DNA fragmentation (DNA ladders and TUNEL assays ). PS exposure(fluorescence-labeled annexin V) MOMP(CYCS release assay) | 29362479; 18846107. |
| | Extrinsic apoptosis – extracellular microenvironment pertubations (Death ligand binding to DRs) | Activation of DRs Recruitment of cytoplasmic adaptor proteins (e.g. FADD and TRADD) Formation of DISC(consist of FADD and procasp-8) Activated Casp8 activates the executioner Casp3 and Casp7. | Casp3 and Casp7 promote DNA fragmentation, PS exposure, and the formation of apoptotic bodies. | Apoptosis morphotype Activation of DRs and specific casp family proteins (Asaay DRs and casp family expression). DNA fragmentation (DNA ladders and TUNEL assays ). PS exposure(fluorescence-labeled annexin V) | 29362479; 18846107. |
| MPT-driven necrosis | Intracellular microenvironment perturbations( such as severe oxidative stress and cytosolic Ca2+ overload) | The opening of PTPC (CYPD as the central component ) Mediating MPT | MPT activiate necrosis. | Necrosis morphotype MPT(ΔΨm assay) The opening of PTPC’(Aaaay CYPD expression) | 29362479; 18846107. |
| Necroptosis | Extracellular and intracellular microenvironment pertubations (death ligand binding to death receptor) | Activation of death receptors RIPK3 activation when casp8 is inative Formation of necrosome(TRADD, RIPK3/RIPK1, MLKL) | Necrosome translocate to the plasma membrane and trigger PMP. | Necrosis morphotype PMP (cell viability test, ATP levels) Formation of necrosome(Assay MLKL and RIPK3/RIPK1 expression). | 29362479; 18846107. |
| Ferroptosis | Specific intracellular microenviroment perturbations(severe lipid peroxidation) | Severe lipid peroxidation relies on ROS generation and iron availability Some of the molecular circuitries regulating the initial steps of ferroptosis(Inhibition of systemXc-, reduced GPX4) | Membrane lipid peroxidation alters PMP, but the precise mechanisms of ferroptosis are largely obscure. | Ferroptosis morphotype Lipid peroxidation(ROS analysis) PMP (cell viability test, ATP levels) | 29362479; 27860262. |
| Pyroptosis | Extracellular and intracellular homeostasis perturbations. | Formation of inflammasome (consist of NLR, ASC and casp1/11 ). Activated casp1/11 drive IL-1b and IL-18 secretion Casp1/11 catalyze the proteolytic cleavage of GSDMD | GSDMD is the key executioner of pyroptosis (Although the precise mechanisms are largely obscure) | Pyroptosis morphotype Activation of casp1/11 and secretion of IL-1b and IL-18 (Asaay casp1/11 and IL-1b/IL-18 expression). PMP (cell viability test, ATP levels) | 29362479; 27404251 |
| Parthanatos | DNA damage, oxidative stress, hypoxia, hypoglycemia, inflammatory cues. | PARP1 hyperactivation causing MPT. Release of AIF into cytoplasm | AIF translocates into nucleus and mediates DNA fragmentation | Parthanatos morphotype PARP1 hyperactivation and AIF release (Asaay PARP1 and AIF expression). MPT(ΔΨm assay) | 29362479; 24684389. |
| Entotic cell death | Detachment of epithelial cells from the extracellular matrix. | The formation of junctions between engulfing and entotic cells that involve the CDH1 and CTNNA1. Cell-in-cell formation (overexpression of RhoA or ROCK I/II leads to actomyosin accumulation at the cell cortex ) | Lipidation of the autophagy protein LC3 onto the entotic vacuole, followed by lysosome fusion and death of internalized cells. | Entotic cell death morphotype Overexpression of RhoA or ROCK I/II (Asaay of RhoA or ROCK I/II expression). | 29362479; 27048820 |

## Slide 17
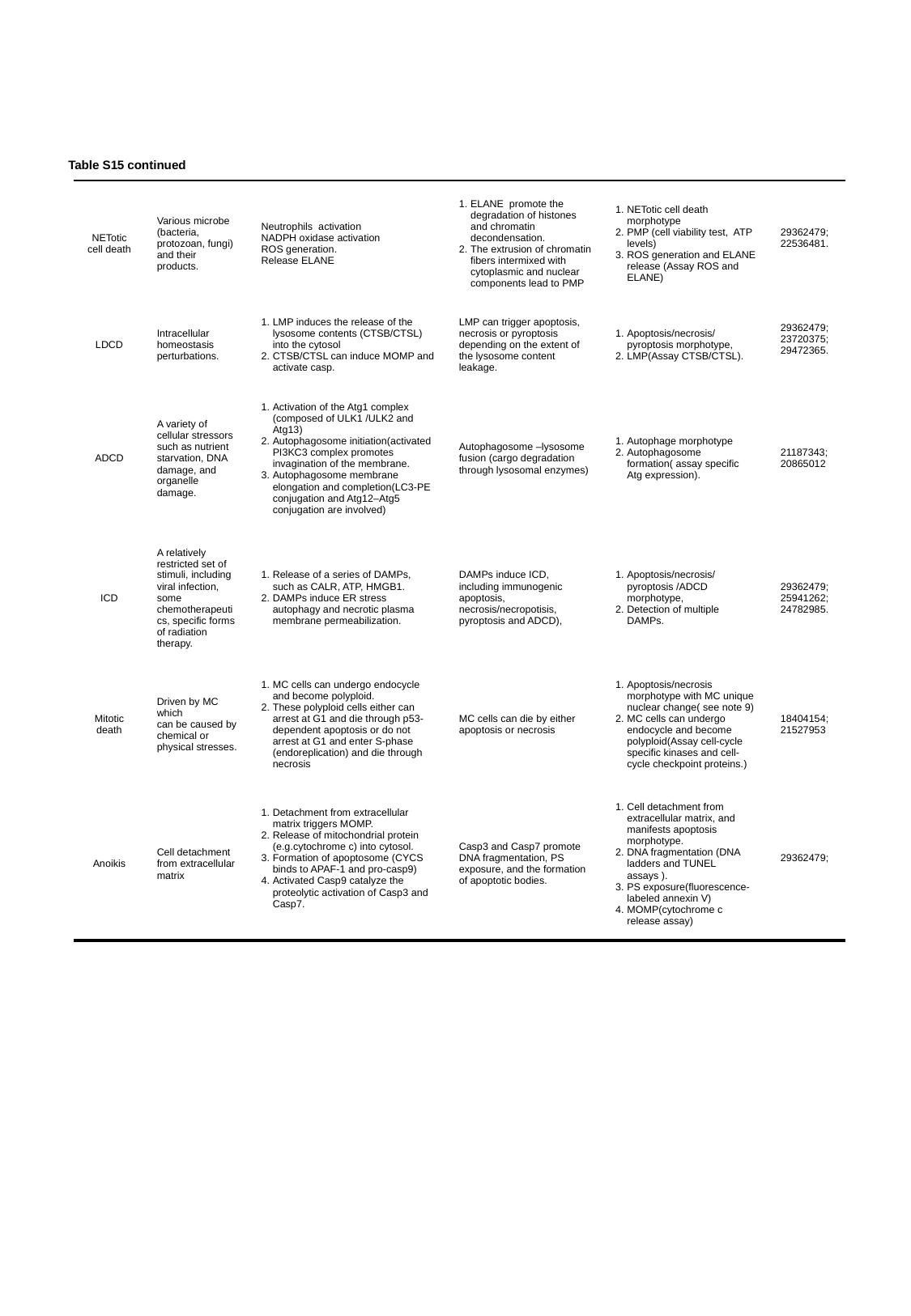

Table S15 continued
| NETotic cell death | Various microbe (bacteria, protozoan, fungi) and their products. | Neutrophils activation NADPH oxidase activation ROS generation. Release ELANE | ELANE promote the degradation of histones and chromatin decondensation. The extrusion of chromatin fibers intermixed with cytoplasmic and nuclear components lead to PMP | NETotic cell death morphotype PMP (cell viability test, ATP levels) ROS generation and ELANE release (Assay ROS and ELANE) | 29362479; 22536481. |
| --- | --- | --- | --- | --- | --- |
| LDCD | Intracellular homeostasis perturbations. | LMP induces the release of the lysosome contents (CTSB/CTSL) into the cytosol CTSB/CTSL can induce MOMP and activate casp. | LMP can trigger apoptosis, necrosis or pyroptosis depending on the extent of the lysosome content leakage. | Apoptosis/necrosis/ pyroptosis morphotype, LMP(Assay CTSB/CTSL). | 29362479; 23720375; 29472365. |
| ADCD | A variety of cellular stressors such as nutrient starvation, DNA damage, and organelle damage. | Activation of the Atg1 complex (composed of ULK1 /ULK2 and Atg13) Autophagosome initiation(activated PI3KC3 complex promotes invagination of the membrane. Autophagosome membrane elongation and completion(LC3-PE conjugation and Atg12–Atg5 conjugation are involved) | Autophagosome –lysosome fusion (cargo degradation through lysosomal enzymes) | Autophage morphotype Autophagosome formation( assay specific Atg expression). | 21187343; 20865012 |
| ICD | A relatively restricted set of stimuli, including viral infection, some chemotherapeutics, specific forms of radiation therapy. | Release of a series of DAMPs, such as CALR, ATP, HMGB1. DAMPs induce ER stress autophagy and necrotic plasma membrane permeabilization. | DAMPs induce ICD, including immunogenic apoptosis, necrosis/necropotisis, pyroptosis and ADCD), | Apoptosis/necrosis/ pyroptosis /ADCD morphotype, Detection of multiple DAMPs. | 29362479; 25941262; 24782985. |
| Mitotic death | Driven by MC which can be caused by chemical or physical stresses. | MC cells can undergo endocycle and become polyploid. These polyploid cells either can arrest at G1 and die through p53-dependent apoptosis or do not arrest at G1 and enter S-phase (endoreplication) and die through necrosis | MC cells can die by either apoptosis or necrosis | Apoptosis/necrosis morphotype with MC unique nuclear change( see note 9) MC cells can undergo endocycle and become polyploid(Assay cell-cycle specific kinases and cell-cycle checkpoint proteins.) | 18404154; 21527953 |
| Anoikis | Cell detachment from extracellular matrix | Detachment from extracellular matrix triggers MOMP. Release of mitochondrial protein (e.g.cytochrome c) into cytosol. Formation of apoptosome (CYCS binds to APAF-1 and pro-casp9) Activated Casp9 catalyze the proteolytic activation of Casp3 and Casp7. | Casp3 and Casp7 promote DNA fragmentation, PS exposure, and the formation of apoptotic bodies. | Cell detachment from extracellular matrix, and manifests apoptosis morphotype. DNA fragmentation (DNA ladders and TUNEL assays ). PS exposure(fluorescence-labeled annexin V) MOMP(cytochrome c release assay) | 29362479; |

## Slide 18
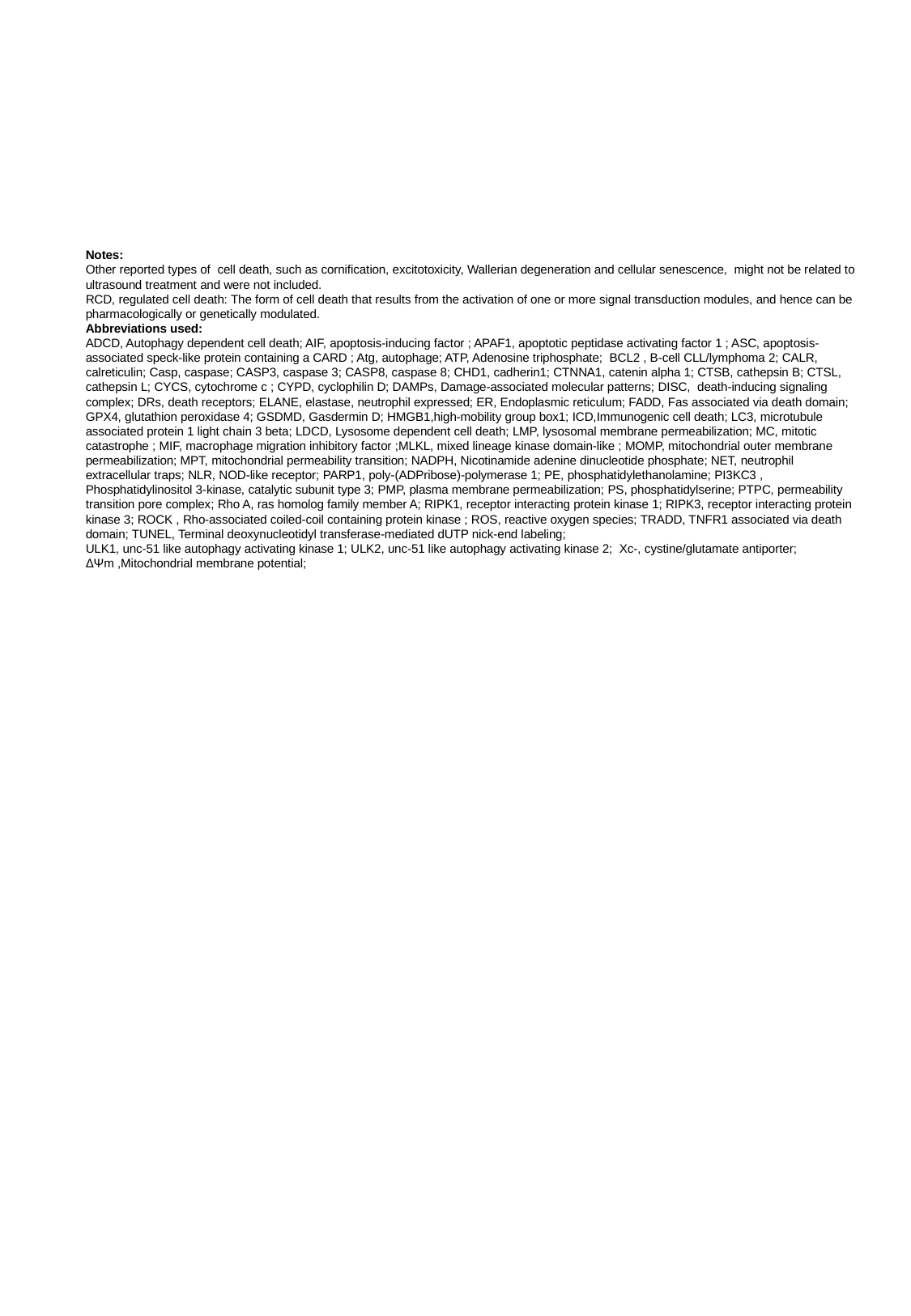

Notes:
Other reported types of cell death, such as cornification, excitotoxicity, Wallerian degeneration and cellular senescence, might not be related to ultrasound treatment and were not included.
RCD, regulated cell death: The form of cell death that results from the activation of one or more signal transduction modules, and hence can be pharmacologically or genetically modulated.
Abbreviations used:
ADCD, Autophagy dependent cell death; AIF, apoptosis-inducing factor ; APAF1, apoptotic peptidase activating factor 1 ; ASC, apoptosis-associated speck-like protein containing a CARD ; Atg, autophage; ATP, Adenosine triphosphate;  BCL2 , B-cell CLL/lymphoma 2; CALR, calreticulin; Casp, caspase; CASP3, caspase 3; CASP8, caspase 8; CHD1, cadherin1; CTNNA1, catenin alpha 1; CTSB, cathepsin B; CTSL, cathepsin L; CYCS, cytochrome c ; CYPD, cyclophilin D; DAMPs, Damage-associated molecular patterns; DISC, death-inducing signaling complex; DRs, death receptors; ELANE, elastase, neutrophil expressed; ER, Endoplasmic reticulum; FADD, Fas associated via death domain; GPX4, glutathion peroxidase 4; GSDMD, Gasdermin D; HMGB1,high-mobility group box1; ICD,Immunogenic cell death; LC3, microtubule associated protein 1 light chain 3 beta; LDCD, Lysosome dependent cell death; LMP, lysosomal membrane permeabilization; MC, mitotic catastrophe ; MIF, macrophage migration inhibitory factor ;MLKL, mixed lineage kinase domain-like ; MOMP, mitochondrial outer membrane permeabilization; MPT, mitochondrial permeability transition; NADPH, Nicotinamide adenine dinucleotide phosphate; NET, neutrophil extracellular traps; NLR, NOD-like receptor; PARP1, poly-(ADPribose)-polymerase 1; PE, phosphatidylethanolamine; PI3KC3 , Phosphatidylinositol 3-kinase, catalytic subunit type 3; PMP, plasma membrane permeabilization; PS, phosphatidylserine; PTPC, permeability transition pore complex; Rho A, ras homolog family member A; RIPK1, receptor interacting protein kinase 1; RIPK3, receptor interacting protein kinase 3; ROCK , Rho-associated coiled-coil containing protein kinase ; ROS, reactive oxygen species; TRADD, TNFR1 associated via death domain; TUNEL, Terminal deoxynucleotidyl transferase-mediated dUTP nick-end labeling;
ULK1, unc-51 like autophagy activating kinase 1; ULK2, unc-51 like autophagy activating kinase 2; Xc-, cystine/glutamate antiporter; ΔΨm ,Mitochondrial membrane potential;

## Slide 19
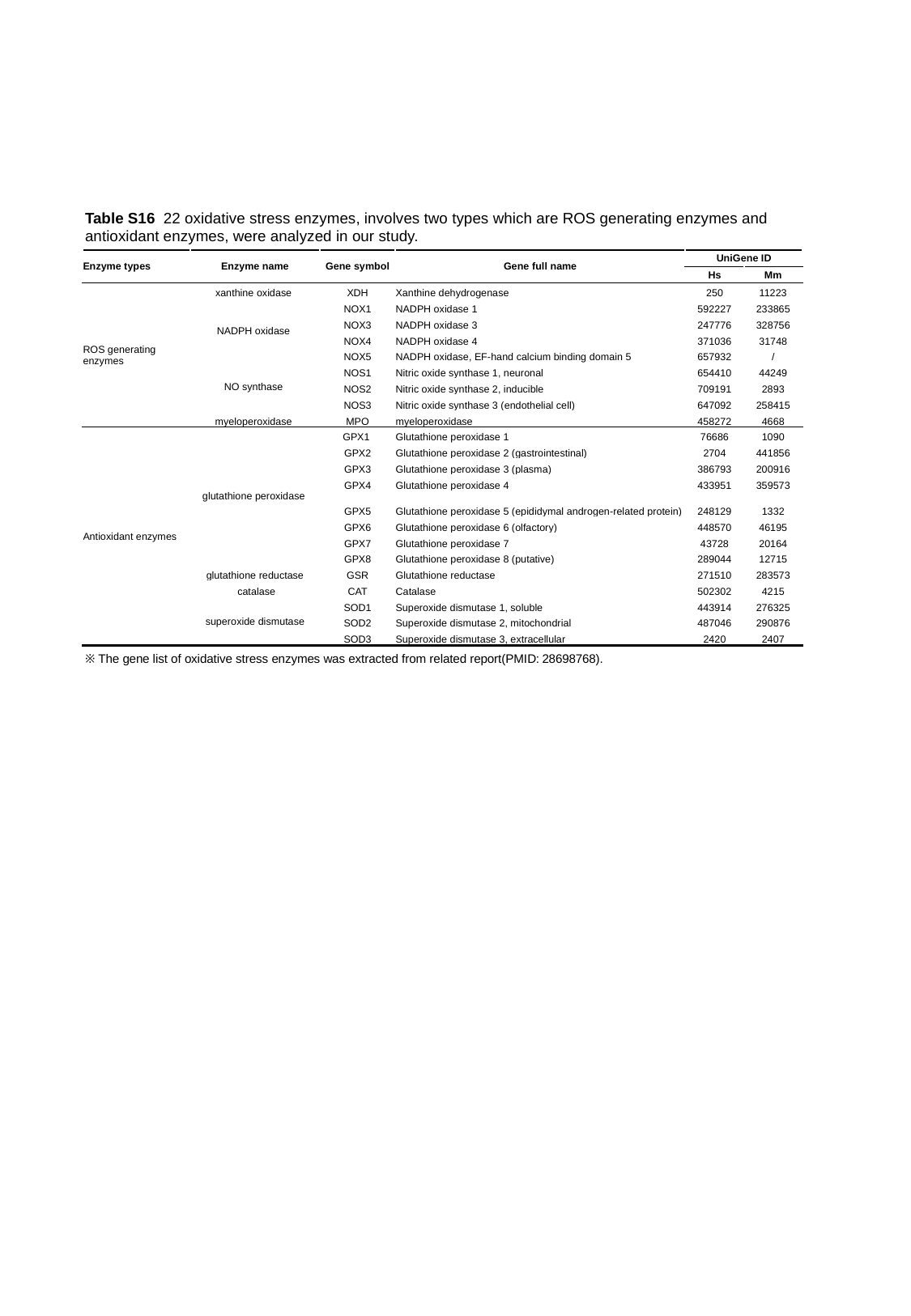

Table S16 22 oxidative stress enzymes, involves two types which are ROS generating enzymes and antioxidant enzymes, were analyzed in our study.
| Enzyme types | Enzyme name | Gene symbol | Gene full name | UniGene ID | |
| --- | --- | --- | --- | --- | --- |
| | | | | Hs | Mm |
| ROS generating enzymes | xanthine oxidase | XDH | Xanthine dehydrogenase | 250 | 11223 |
| | NADPH oxidase | NOX1 | NADPH oxidase 1 | 592227 | 233865 |
| | | NOX3 | NADPH oxidase 3 | 247776 | 328756 |
| | | NOX4 | NADPH oxidase 4 | 371036 | 31748 |
| | | NOX5 | NADPH oxidase, EF-hand calcium binding domain 5 | 657932 | / |
| | NO synthase | NOS1 | Nitric oxide synthase 1, neuronal | 654410 | 44249 |
| | | NOS2 | Nitric oxide synthase 2, inducible | 709191 | 2893 |
| | | NOS3 | Nitric oxide synthase 3 (endothelial cell) | 647092 | 258415 |
| | myeloperoxidase | MPO | myeloperoxidase | 458272 | 4668 |
| Antioxidant enzymes | glutathione peroxidase | GPX1 | Glutathione peroxidase 1 | 76686 | 1090 |
| | | GPX2 | Glutathione peroxidase 2 (gastrointestinal) | 2704 | 441856 |
| | | GPX3 | Glutathione peroxidase 3 (plasma) | 386793 | 200916 |
| | | GPX4 | Glutathione peroxidase 4 | 433951 | 359573 |
| | | GPX5 | Glutathione peroxidase 5 (epididymal androgen-related protein) | 248129 | 1332 |
| | | GPX6 | Glutathione peroxidase 6 (olfactory) | 448570 | 46195 |
| | | GPX7 | Glutathione peroxidase 7 | 43728 | 20164 |
| | | GPX8 | Glutathione peroxidase 8 (putative) | 289044 | 12715 |
| | glutathione reductase | GSR | Glutathione reductase | 271510 | 283573 |
| | catalase | CAT | Catalase | 502302 | 4215 |
| | superoxide dismutase | SOD1 | Superoxide dismutase 1, soluble | 443914 | 276325 |
| | | SOD2 | Superoxide dismutase 2, mitochondrial | 487046 | 290876 |
| | | SOD3 | Superoxide dismutase 3, extracellular | 2420 | 2407 |
※ The gene list of oxidative stress enzymes was extracted from related report(PMID: 28698768).

## Slide 20
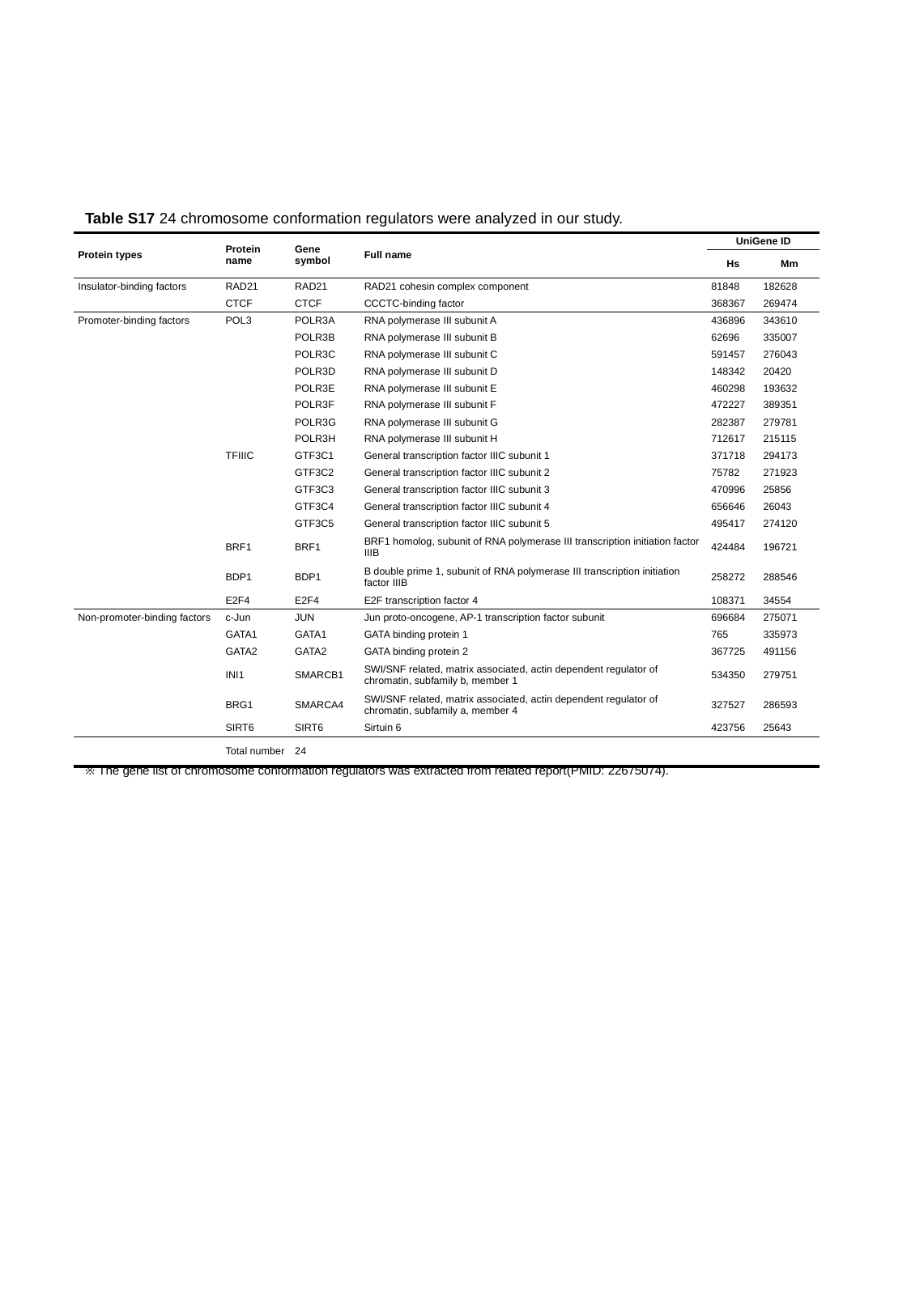

Table S17 24 chromosome conformation regulators were analyzed in our study.
| Protein types | Protein name | Gene symbol | Full name | UniGene ID | |
| --- | --- | --- | --- | --- | --- |
| | | | | Hs | Mm |
| Insulator-binding factors | RAD21 | RAD21 | RAD21 cohesin complex component | 81848 | 182628 |
| | CTCF | CTCF | CCCTC-binding factor | 368367 | 269474 |
| Promoter-binding factors | POL3 | POLR3A | RNA polymerase III subunit A | 436896 | 343610 |
| | | POLR3B | RNA polymerase III subunit B | 62696 | 335007 |
| | | POLR3C | RNA polymerase III subunit C | 591457 | 276043 |
| | | POLR3D | RNA polymerase III subunit D | 148342 | 20420 |
| | | POLR3E | RNA polymerase III subunit E | 460298 | 193632 |
| | | POLR3F | RNA polymerase III subunit F | 472227 | 389351 |
| | | POLR3G | RNA polymerase III subunit G | 282387 | 279781 |
| | | POLR3H | RNA polymerase III subunit H | 712617 | 215115 |
| | TFIIIC | GTF3C1 | General transcription factor IIIC subunit 1 | 371718 | 294173 |
| | | GTF3C2 | General transcription factor IIIC subunit 2 | 75782 | 271923 |
| | | GTF3C3 | General transcription factor IIIC subunit 3 | 470996 | 25856 |
| | | GTF3C4 | General transcription factor IIIC subunit 4 | 656646 | 26043 |
| | | GTF3C5 | General transcription factor IIIC subunit 5 | 495417 | 274120 |
| | BRF1 | BRF1 | BRF1 homolog, subunit of RNA polymerase III transcription initiation factor IIIB | 424484 | 196721 |
| | BDP1 | BDP1 | B double prime 1, subunit of RNA polymerase III transcription initiation factor IIIB | 258272 | 288546 |
| | E2F4 | E2F4 | E2F transcription factor 4 | 108371 | 34554 |
| Non-promoter-binding factors | c-Jun | JUN | Jun proto-oncogene, AP-1 transcription factor subunit | 696684 | 275071 |
| | GATA1 | GATA1 | GATA binding protein 1 | 765 | 335973 |
| | GATA2 | GATA2 | GATA binding protein 2 | 367725 | 491156 |
| | INI1 | SMARCB1 | SWI/SNF related, matrix associated, actin dependent regulator of chromatin, subfamily b, member 1 | 534350 | 279751 |
| | BRG1 | SMARCA4 | SWI/SNF related, matrix associated, actin dependent regulator of chromatin, subfamily a, member 4 | 327527 | 286593 |
| | SIRT6 | SIRT6 | Sirtuin 6 | 423756 | 25643 |
| | Total number | 24 | | | |
※ The gene list of chromosome conformation regulators was extracted from related report(PMID: 22675074).

## Slide 21
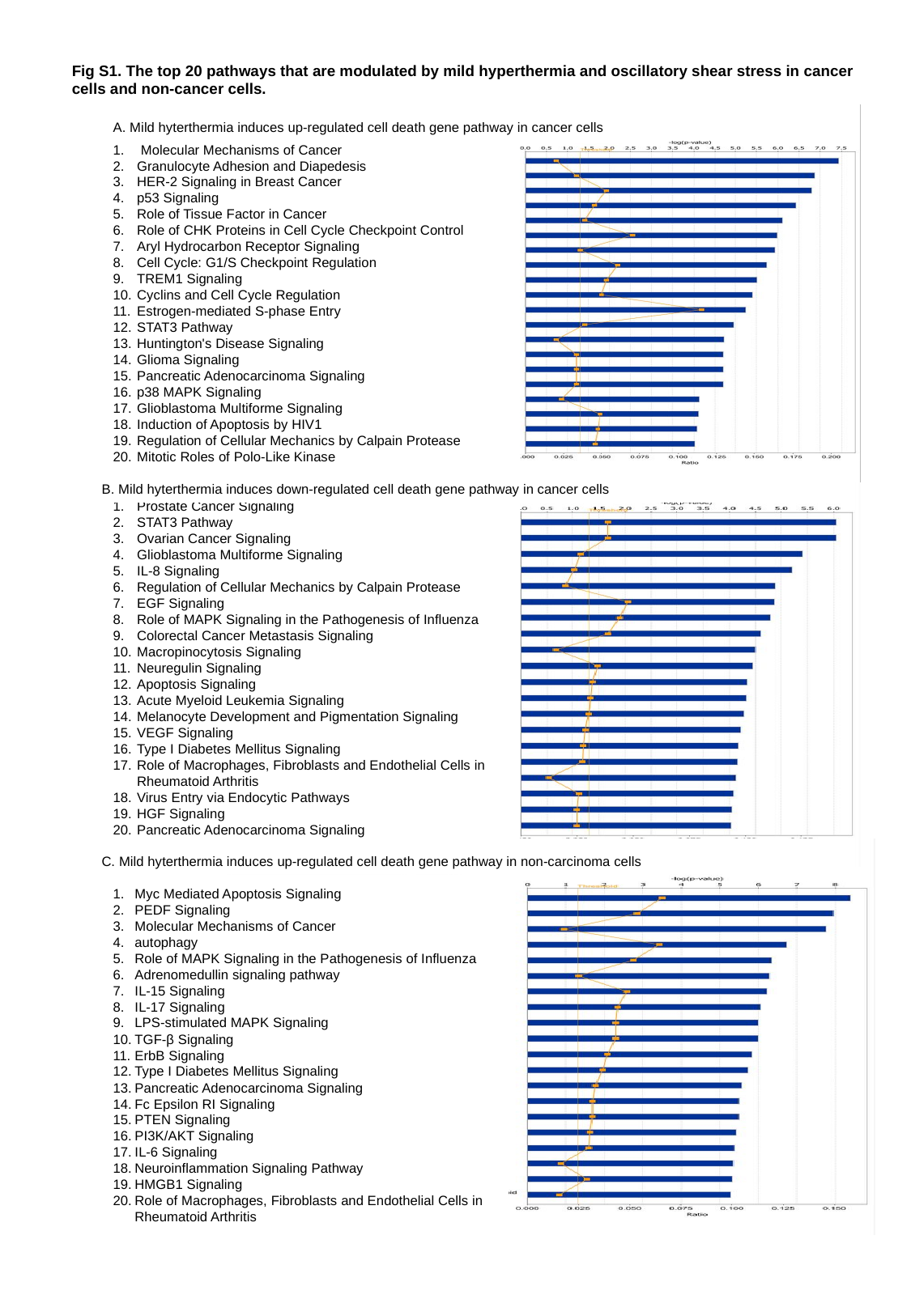

# Fig S1. The top 20 pathways that are modulated by mild hyperthermia and oscillatory shear stress in cancer cells and non-cancer cells.
A. Mild hyterthermia induces up-regulated cell death gene pathway in cancer cells
 Molecular Mechanisms of Cancer
Granulocyte Adhesion and Diapedesis
HER-2 Signaling in Breast Cancer
p53 Signaling
Role of Tissue Factor in Cancer
Role of CHK Proteins in Cell Cycle Checkpoint Control
Aryl Hydrocarbon Receptor Signaling
Cell Cycle: G1/S Checkpoint Regulation
TREM1 Signaling
Cyclins and Cell Cycle Regulation
Estrogen-mediated S-phase Entry
STAT3 Pathway
Huntington's Disease Signaling
Glioma Signaling
Pancreatic Adenocarcinoma Signaling
p38 MAPK Signaling
Glioblastoma Multiforme Signaling
Induction of Apoptosis by HIV1
Regulation of Cellular Mechanics by Calpain Protease
Mitotic Roles of Polo-Like Kinase
B. Mild hyterthermia induces down-regulated cell death gene pathway in cancer cells
Prostate Cancer Signaling
STAT3 Pathway
Ovarian Cancer Signaling
Glioblastoma Multiforme Signaling
IL-8 Signaling
Regulation of Cellular Mechanics by Calpain Protease
EGF Signaling
Role of MAPK Signaling in the Pathogenesis of Influenza
Colorectal Cancer Metastasis Signaling
Macropinocytosis Signaling
Neuregulin Signaling
Apoptosis Signaling
Acute Myeloid Leukemia Signaling
Melanocyte Development and Pigmentation Signaling
VEGF Signaling
Type I Diabetes Mellitus Signaling
Role of Macrophages, Fibroblasts and Endothelial Cells in Rheumatoid Arthritis
Virus Entry via Endocytic Pathways
HGF Signaling
Pancreatic Adenocarcinoma Signaling
C. Mild hyterthermia induces up-regulated cell death gene pathway in non-carcinoma cells
Myc Mediated Apoptosis Signaling
PEDF Signaling
Molecular Mechanisms of Cancer
autophagy
Role of MAPK Signaling in the Pathogenesis of Influenza
Adrenomedullin signaling pathway
IL-15 Signaling
IL-17 Signaling
LPS-stimulated MAPK Signaling
TGF-β Signaling
ErbB Signaling
Type I Diabetes Mellitus Signaling
Pancreatic Adenocarcinoma Signaling
Fc Epsilon RI Signaling
PTEN Signaling
PI3K/AKT Signaling
IL-6 Signaling
Neuroinflammation Signaling Pathway
HMGB1 Signaling
Role of Macrophages, Fibroblasts and Endothelial Cells in Rheumatoid Arthritis

## Slide 22
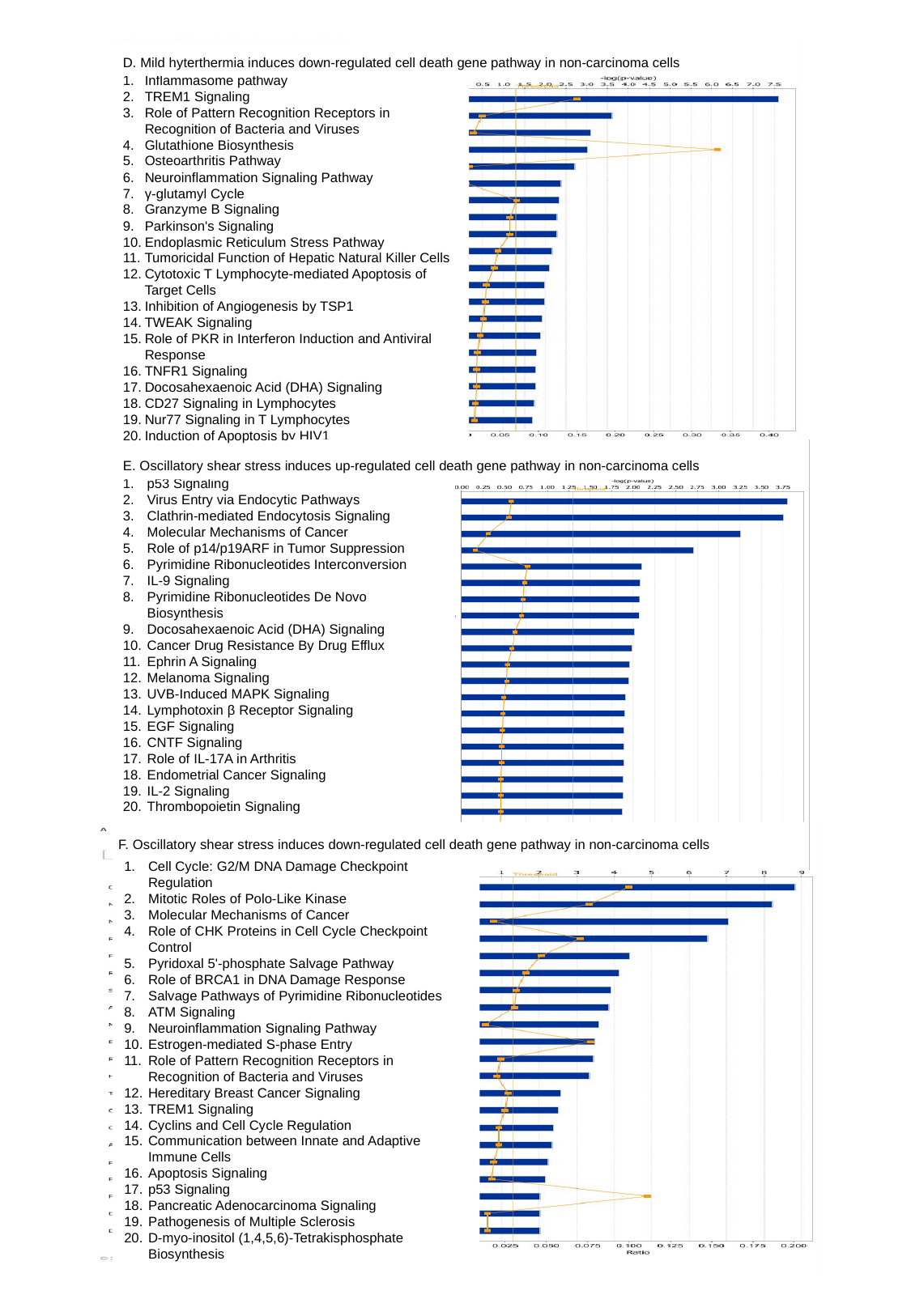

D. Mild hyterthermia induces down-regulated cell death gene pathway in non-carcinoma cells
Inflammasome pathway
TREM1 Signaling
Role of Pattern Recognition Receptors in Recognition of Bacteria and Viruses
Glutathione Biosynthesis
Osteoarthritis Pathway
Neuroinflammation Signaling Pathway
γ-glutamyl Cycle
Granzyme B Signaling
Parkinson's Signaling
Endoplasmic Reticulum Stress Pathway
Tumoricidal Function of Hepatic Natural Killer Cells
Cytotoxic T Lymphocyte-mediated Apoptosis of Target Cells
Inhibition of Angiogenesis by TSP1
TWEAK Signaling
Role of PKR in Interferon Induction and Antiviral Response
TNFR1 Signaling
Docosahexaenoic Acid (DHA) Signaling
CD27 Signaling in Lymphocytes
Nur77 Signaling in T Lymphocytes
Induction of Apoptosis by HIV1
E. Oscillatory shear stress induces up-regulated cell death gene pathway in non-carcinoma cells
p53 Signaling
Virus Entry via Endocytic Pathways
Clathrin-mediated Endocytosis Signaling
Molecular Mechanisms of Cancer
Role of p14/p19ARF in Tumor Suppression
Pyrimidine Ribonucleotides Interconversion
IL-9 Signaling
Pyrimidine Ribonucleotides De Novo Biosynthesis
Docosahexaenoic Acid (DHA) Signaling
Cancer Drug Resistance By Drug Efflux
Ephrin A Signaling
Melanoma Signaling
UVB-Induced MAPK Signaling
Lymphotoxin β Receptor Signaling
EGF Signaling
CNTF Signaling
Role of IL-17A in Arthritis
Endometrial Cancer Signaling
IL-2 Signaling
Thrombopoietin Signaling
F. Oscillatory shear stress induces down-regulated cell death gene pathway in non-carcinoma cells
Cell Cycle: G2/M DNA Damage Checkpoint Regulation
Mitotic Roles of Polo-Like Kinase
Molecular Mechanisms of Cancer
Role of CHK Proteins in Cell Cycle Checkpoint Control
Pyridoxal 5'-phosphate Salvage Pathway
Role of BRCA1 in DNA Damage Response
Salvage Pathways of Pyrimidine Ribonucleotides
ATM Signaling
Neuroinflammation Signaling Pathway
Estrogen-mediated S-phase Entry
Role of Pattern Recognition Receptors in Recognition of Bacteria and Viruses
Hereditary Breast Cancer Signaling
TREM1 Signaling
Cyclins and Cell Cycle Regulation
Communication between Innate and Adaptive Immune Cells
Apoptosis Signaling
p53 Signaling
Pancreatic Adenocarcinoma Signaling
Pathogenesis of Multiple Sclerosis
D-myo-inositol (1,4,5,6)-Tetrakisphosphate Biosynthesis

## Slide 23
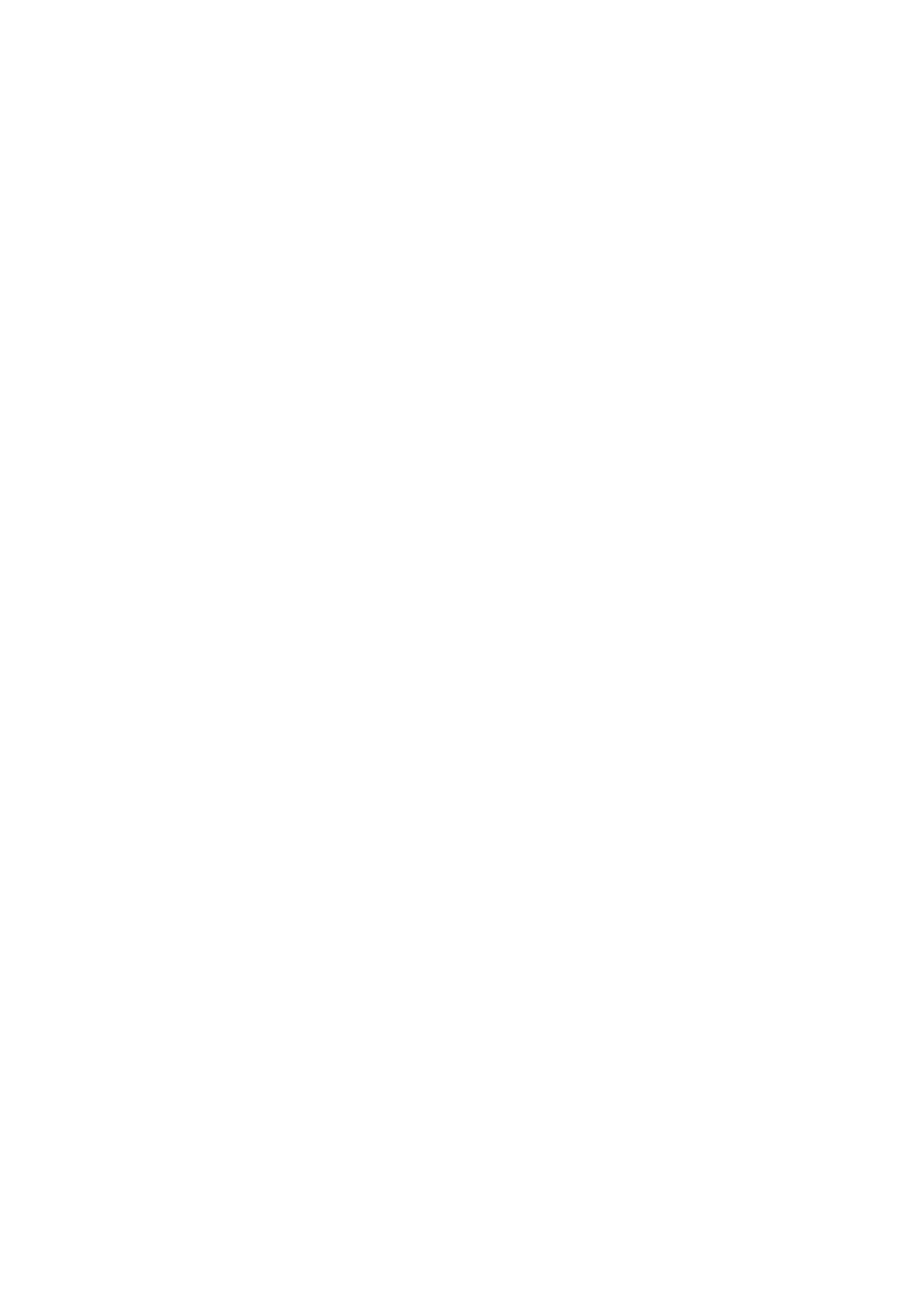

Supplement: Supplementary file 1 [file Presentation_1.PPTX]
